# Identification and expression profile analysis of chemosensory genes in pine needle gall midge, *Thecodiplosis japonensis* (Diptera: Cecidomyiidae)

**DOI:** 10.3389/fphys.2023.1123479

**Published:** 2023-02-16

**Authors:** Jipeng Jiao, Rui Zhu, Lili Ren, Jing Tao, Youqing Luo

**Affiliations:** ^1^ Key Laboratory for Forest Pest Control, College of Forestry, Beijing Forestry University, Beijing, China; ^2^ Sino-French Joint Laboratory for Invasive Forest Pests in Eurasia, Beijing Forestry University/French National Research Institute for Agriculture, Food and Environment (INRAE), Beijing, China

**Keywords:** *Thecodiplosis japonensis*, chemosensory proteins, odorant-binding proteins, odorant receptors, expression profiles

## Abstract

Insects have highly specialized and sensitive olfactory systems involving several chemosensory genes to locate their mates and hosts or escape from predators. Pine needle gall midge, *Thecodiplosis japonensis* (Diptera: Cecidomyiidae), has invaded China since 2016 and caused serious damage. Till now, there is no environmentally friendly measure to control this gall midge. Screening molecules with high affinity to target odorant-binding protein to develop highly efficient attractants is a potential pest management method. However, the chemosensory genes in *T. japonensis* are still unclear. We identified 67 chemosensory-related genes in the transcriptomes of antennae, including 26 OBPs, 2 CSPs, 17 ORs, 3 SNMPs, 6 GRs, and 13 IRs, using high throughput sequencing. Phylogenetic analysis of these six chemosensory gene families among Dipteran was performed to classify and predict the functions. The expression profiles of OBPs, CSPs and ORs were validated by quantitative real-time PCR. 16 of the 26 OBPs were biased expressed in antennae. *TjapORco* and *TjapOR5* were highly expressed in the antenna of unmated male and female adults. The functions of related OBPs and ORs genes were also discussed. These results provide a basis for the functional research on chemosensory genes at the molecular level.

## 1 Introduction

Insects have sensitive olfactory systems to perceive odorant compounds and guide their important behaviors, including host location, feeding, mating, oviposition, and escape from harm (Zhang et al., 2017; Turlings and Erb, 2018; Guo and Wang, 2019). As the main olfactory organ of insects, antennae have a variety of olfactory sensilla on the surface (Zacharuk, 1980; Steinbrecht, 1997; Binyameen et al., 2012). The odorant molecules enter the cavity of the olfactory sensilla through the micropores on the wall of the olfactory sensilla, pass through the lymphatic fluid of the sensilla, reach the Olfactory receptor neurons (ORNs) dendrites, activate the receptor proteins on the dendrites, and finally cause different behavioral responses of insects (Pelosi, 1996; Krieger and Breer, 1999; Wilson and Mainen, 2006; Hansson and Stensmyr, 2011). The study of insects’ olfactory mechanisms is progressing owing to advancements in biochemistry, molecular biology, insect behavior, and electrophysiology (Vogt and Riddiford, 1981; Wetzel et al., 2001; Leal, 2005; Forstner et al., 2008; Allen and Wanner, 2011; Hua et al., 2013; Leal, 2013; Wang et al., 2020). There are several olfactory proteins involved in the odorant’s perception (Pelosi et al., 2006). The odorant molecules are delivered by odorant-binding proteins (OBPs) or chemosensory proteins (CSPs) in sensilla lymph fluid (Vogt and Riddiford, 1981; Vogt et al., 1991; Pelosi, 1994; Pelosi et al., 2005; Pelosi et al., 2014; Liu et al., 2018). Then, molecules are detected by odorant receptors (ORs) expressed on the olfactory neuron membrane. Meanwhile, the chemical signal is converted to electrical signals (Clyne et al., 1999; Gao and Chess, 1999; Vosshall et al., 1999; Wicher et al., 2008; Butterwick et al., 2018). Additionally, gustatory receptors (GRs), ionotropic receptors (IRs) and sensory neuron membrane proteins (SNMPs) are also involved (Jones et al., 2007; Benton et al., 2009; Xu et al., 2015; Ning et al., 2016). GRs could detect carbon dioxide, sugars, pheromones, or bitter flavors (Kent et al., 2008; Robertson and Kent, 2009). IRs are essential for odor-evoked neuronal responses and for detecting environmental volatile chemicals and tastes (Croset et al., 2010; Rytz et al., 2013). Sensory neuron membrane proteins (SNMPs) are hypothesized to act in concert with odorant receptors to detect pheromone molecules in a population of olfactory sensory neurons (OSNs) (Benton et al., 2007; Jin et al., 2008; Liu et al., 2013). The signal molecules are degraded by odorant degrading enzymes in lymphatic or sensorial cells (Vogt and Riddiford, 1981; Leal, 2005; Leal, 2013).

Pine needle gall midge, *T. japonensis* (Uchida and Inouye), a microscopic forest pest belonging to the Cecidomyiidae family, which contains more than 6,600 known species (Gagné et al., 2021). First reported in Japan in 1901, the pest was discovered in South Korea in 1924, causing serious damage to pine trees such as *P. thunbergii* Parlatore and *P. densiflora* Sieb. et Zucc, resulting in huge ecological and economic losses. *T. japonensis* has become an important pest to pine trees in South Korea (Choi et al., 2019). *T. japonensis* has invaded China in 2016 and caused serious damages to local *Pinus thunbergii*, *P. densiflora*, and *P. tabulaeformis* (Duan et al., 2021).

Adult pine needle gall midges mate soon after emerging from pupation sites in the surface soil in June. The females lay eggs on needle pairs of shoots. Like other Cecidomyiids, adults of *T. japonensis* have a lifespan of 1-2 days and do not feed (Harris et al., 2003). Galls are formed when larvae grow in the base needle pairs, causing the stopping of needle growth (Skuhravá and Roques, 2000). Currently, there are no environmentally friendly control measures except traditional chemical pesticides.

Effectively interfering with the olfactory perception process is a potential way to achieve sustainable pest management. One of the future approaches to pest management is the use of attractants and repellents to disrupt behavior to achieve green control and reduce the use of conventional insecticides. For example, the sex pheromone of the *Mayetiola destructor* (Hessian Fly), *Sitodiplosis mosellana* (Orange Wheat Blossom Midge), and *Contarinia nasturtii* (Swede Midge) have been made into a commercial lure and is widely used for trapping and monitoring pests (Witzgall et al., 2008). The oviposition attractants, targeting olfactory proteins, have been successfully screened for the prevention and control of *Culex quinquefasciatus* (Leal et al., 2008).

Identifying chemosensory genes and studies into the olfactory mechanism is essential for pest control because they interfere with olfactory perception. The chemosensory gene families have been extensively studied in two model organisms, namely, *Drosophila melanogaster* and *Anopheles gambiae* for years (Hill et al., 2002; Robertson et al., 2003; Rinker et al., 2013a; Rinker et al., 2013b). Since then, several chemosensory-related genes have been identified in Diptera insects, including *Aedes aegypti*, *C. quinquefasciatus* (Pelletier and Leal, 2011; Manoharan et al., 2013), *Anopheles sinensis* (He et al., 2016), *Delia platura* (Ohta et al., 2015), *Bactrocera cucurbitae* (Elfekih et al., 2016), *B. dorsalis* (Liu et al., 2016), and so on. However, the chemosensory gene families in herbivorous Cecidomyiids species were poorly studied.

This study aims to identify the chemosensory genes and characterize the chemosensation of *T. japonensis*. We performed transcriptome analysis of the antennae of *T. japonensis*, and 67 chemosensory genes were identified. The real-time quantitative polymerase chain reaction was performed to study the transcription levels of OBPs, CSPs, and ORs in different tissues of both female and male gall midges. The results may provide in-depth information about chemosensory genes at the molecular level.

## 2 Results

### 2.1 Transcriptome sequencing and assembly

The antennal RNA-seq raw reads (PE150) were generated by Illumina Novaseq. A total of 286,459,200 Raw reads were obtained, and 278,011,612 Clean reads were obtained after quality control. The GC content of all samples was greater than 35%, the Q20 bases were greater than 97%, and the Q30 bases were greater than 93%. After *de novo* assembly, 83,080 transcripts were obtained with an average length of 922 bp. The N50 length was up to 1966 bp. All transcripts ranged from 201 bp—35,601 bp in length. The result of BUSCO assessment showed that the complete BUSCOs accounted for 93.0%, with 85.9% single copy and 7.1% multiple copies.

### 2.2 Homology analysis and gene ontology annotation

All transcripts from *T. japonensis* were annotated in six databases (NR, Pfam, GO, KEGG, COG, and Swiss-Prot). Among the 83,080 transcripts, 16,837 (20.27%) transcripts were annotated to the GO database, 13,489 (16.24%) to the KEGG database, 19,798 (23.83%) to the COG database, 20,088 (24.18%) to the NR database, 16,840 (20.27%) to the Swiss-Prot database, and 17,898 (21.54%) to the Pfam database. NR database homology searches showed that the *T. japonensis* antennal transcriptome shared the greatest homology with sequences from *A. aegypti* (6.97%), followed by *A. albopictus* (6.43%) and *C. quinquefasciatus* (3.07%). Among the transcripts annotated to the GO database, 35% were annotated to the biological process, 29% to molecular function, and 36% to the cellular component. In the biological process category, the frequency of the cellular process is the highest, followed by the metabolic process. In the cell component category, the frequency of the cell part is the highest. The most annotated functional proteins in the molecular function category include binding and catalytic activity (Supplementary Figure S1).

### 2.3 Identification and analysis of chemosensory-related genes

#### 2.3.1 Odorant-binding proteins (OBPs)

26 OBPs were identified in the *T. japonensis* antennal transcriptomes. Except for *TjapOBP26*, 25 OBPs have complete Open Reading Frames (ORFs) ranging from 115 to 169 amino acids in length and 18 OBPs have signal peptides. All 26 TjapOBPs hit the Insect pheromone/odorant binding protein domains (smart00708) and pheromone/general odorant binding protein family (pfam01395). The FPKM values of 24 *TjapOBPs* were greater than 1. Of these, *TjapOBP6* and *TjapOBP20* were highly expressed, with FPKM values of 75,423.09 and 24245.72, respectively. We divided 26 OBPs into Classic, Plus-C and Minus-C subfamilies according to the position and number of conserved cysteines (Supplementary Table S1). Most TjapOBPs belong to classical subfamily, which presents the pattern of six conserved cysteines of C1-X26-37-C2-X3-C3-X32-41-C4-X7-10-C5-X8-C6. We also identified one plus-C subfamily *TjapOBP22*, which presented two additional conserved cysteines behind conserved C6 and a conserved proline after the conserved C6 of C1-X37-C2-X3-C3-X42-C4-X24-C5-X8-C6-P-X10-C7-X9-C8. *TjapOBP13* (C1-X32-C3-X38-C4-X17-C6) and *TjapOBP18* (C1-X25-C2-X3-C3-X46-C4-X18-C6) were identified as Minus-C subfamily, with only 4 or 5 conserved cysteines.

**FIGURE 1:**
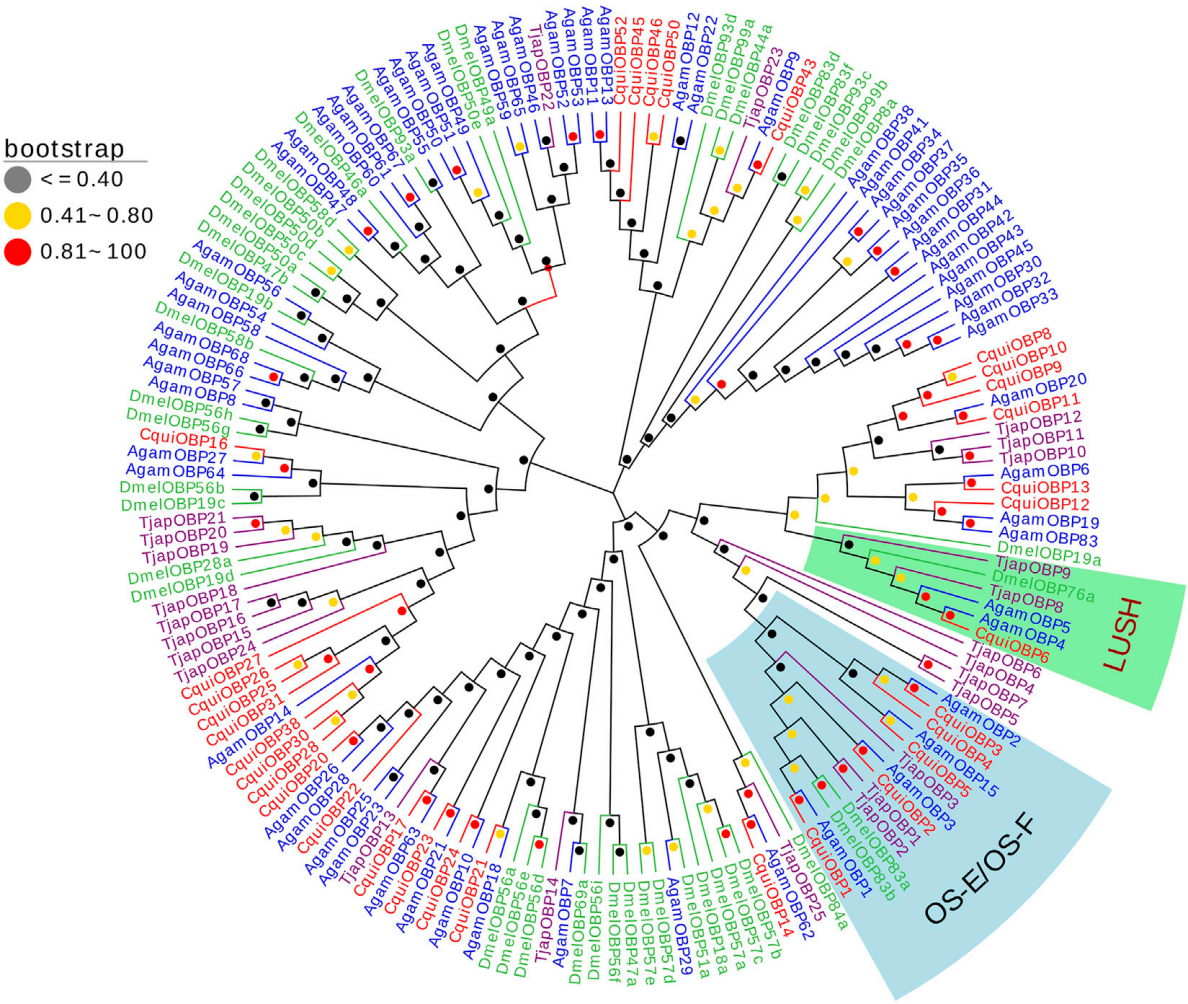
The NJ phylogenetic analysis of OBPs in *T. japonensis*, *C. quinquefasciatus*, *A. gambiae*, and *D. melanogaster*. The LUSH group and OS-E/OS-F group are shown.

We constructed a phylogenetic Neighbor-joining tree of OBPs of *T. japonensis* with OBPs from other Dipteran insects. In the phylogenetic tree, most TjapOBPs were clustered with other Dipteran OBPs. Notably, *TjapOBP8/9* were clustered with *DmelOBP76a* (pheromone-binding protein, LUSH) of *D. melanogaster*, and *TjapOBP1/2/3* were clustered with *DmelOBP83a/83b* (OS-E/OS-F, an OBP group co-expressed with LUSH and involved in pheromone detection) of *D. melanogaster* (Shanbhag et al., 2001; Ha and Smith, 2006) (Figure 1).

#### 2.3.2 Chemosensory proteins (CSPs)

**FIGURE 2:**
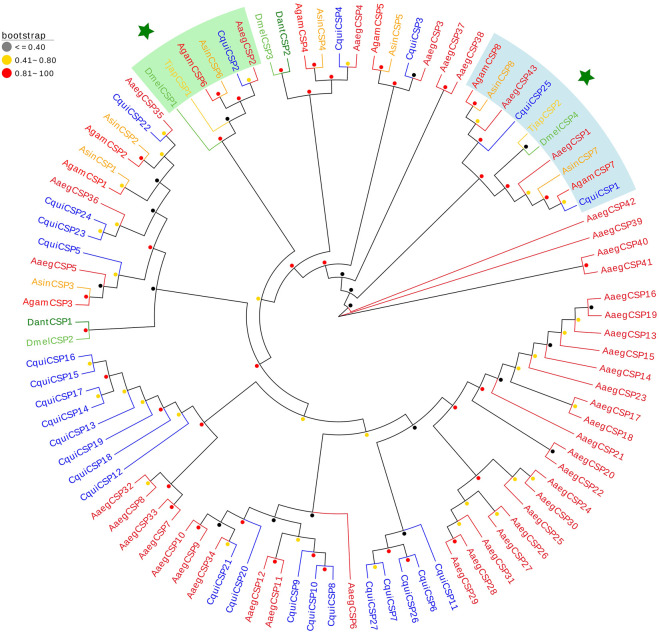
The NJ phylogenetic analysis of CSPs in *T. japonensis*, *C. quinquefasciatus*, *D. antiqua*, *A. aegypti*, *A. gambiae*, *A. sinensis* and *D. melanogaster*. Two TjapCSPs clustered into two groups are shown. The *TjapCSP1* and *TjapCSP2* were marked with star.

We identified two TjapCSPs in the antennal transcriptomes of *T. japonensis* that encode proteins full ORF, which length was 122 aa and 112 aa, and have signal peptides (Supplementary Table S2). The two TjapCSPs have four conserved cysteine residues (C1-X6-C2-X18-C3-X2-C4). The FPKM values of the two CSPs were 245 and 17, respectively. A phylogenetic tree was established using the CSPs sequences to analyze the relationships of the CSPs in *T. japonensis* with other Dipteran insects, including *A. sinensis, A. aegypti, C. quinquefasciatus, A. gambiae, Delia antiqua* and *D. melanogaster* (Figure 1). In the phylogenetic tree, two TjapCSPs were clustered into two groups with the CSPs of *D. melanogaster*, respectively (Figure 2).

#### 2.3.3 Odorant receptors (ORs)

**FIGURE 3:**
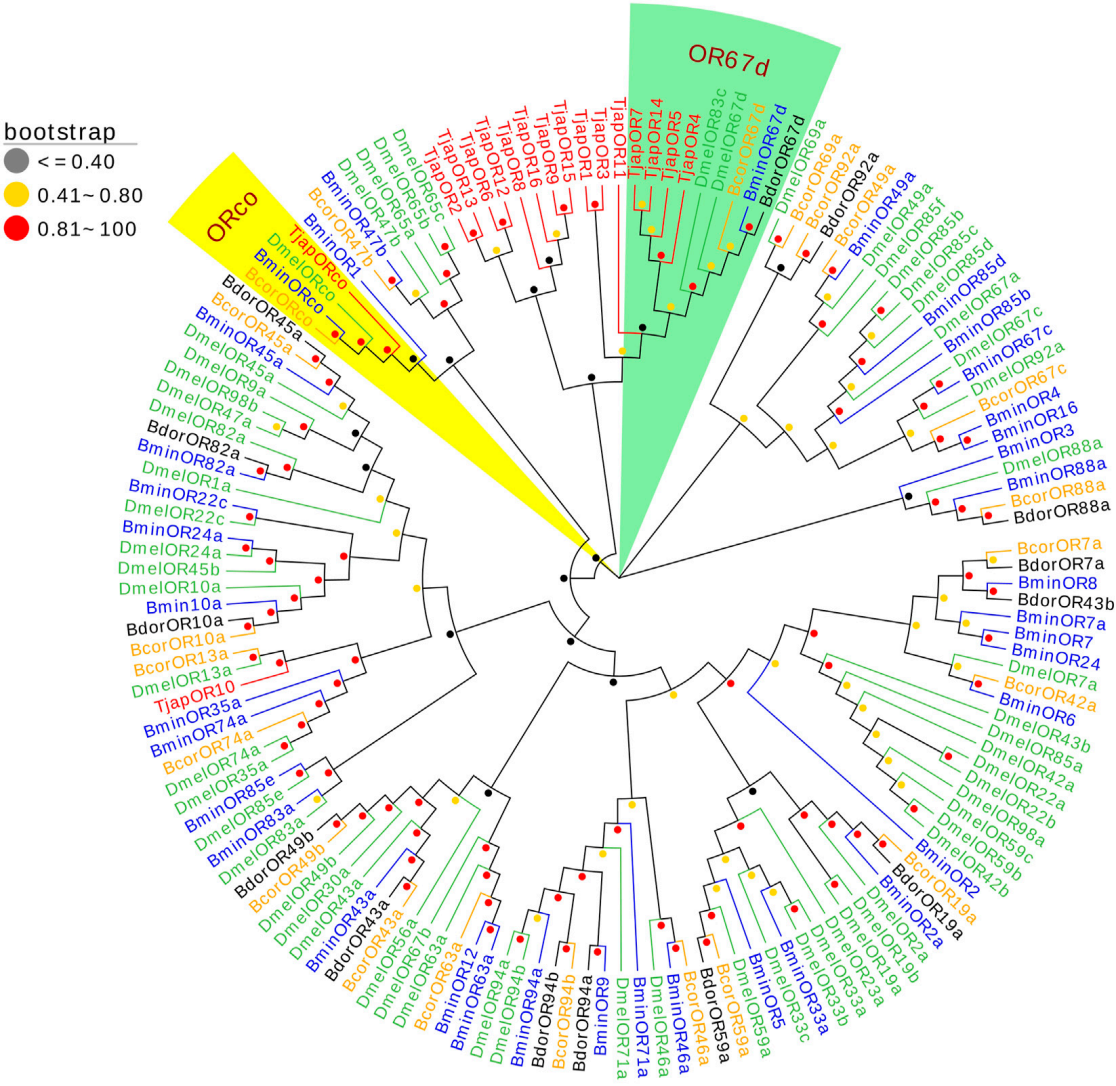
The NJ phylogenetic analysis of ORs in *T. japonensis*, *B. minax*, *B. correcta*, *B. dorsalis*, and *D. melanogaster*. The ORco group and OR67d group are shown.

We identified 17 ORs in the antennal transcriptomes of *T. japonensis*. 11 ORs have complete ORFs ranging from 317 aa to 467 aa in length with 4-8 predicted transmembrane domains, and six genes had partial ORFs in the 5′or 3′regions (Supplementary Table S3). *TjapOR15* and *TjapORco* were highly expressed, with FPKM values of 388.78 and 106.96, respectively. A phylogenetic tree was established using the ORs sequences to analyze the relationship of the ORs in *T. japonensis* with other Dipteran insects, including *Bactrocera minax*, *B. correcta*, *B. dorsalis* and *D. melanogaster*. *TjapORco* was clustered in a highly conserved clade with other Dipteran insects. Eight ORs of *T. japonensis* cluster into a relative “species expansion” group, and *TjapOR10* is clustered with *DmelOR13* of *D. melanogaster*. In addition, four ORs of *T. japonensis* (*TjapOR4/5/7/14*) clustered with the pheromone receptor *DmelOR67d* of *D. melanogaster*, *BcorOR67d* of *B. correcta*, *BminOR67d* of *B. minax*, and *BdorOR67d* of *B. dorsalis*, to form the *OR67d* branch with high bootstrap support value (Figure 3).

#### 2.3.4 Gustatory receptors (GRs)

**FIGURE 4:**
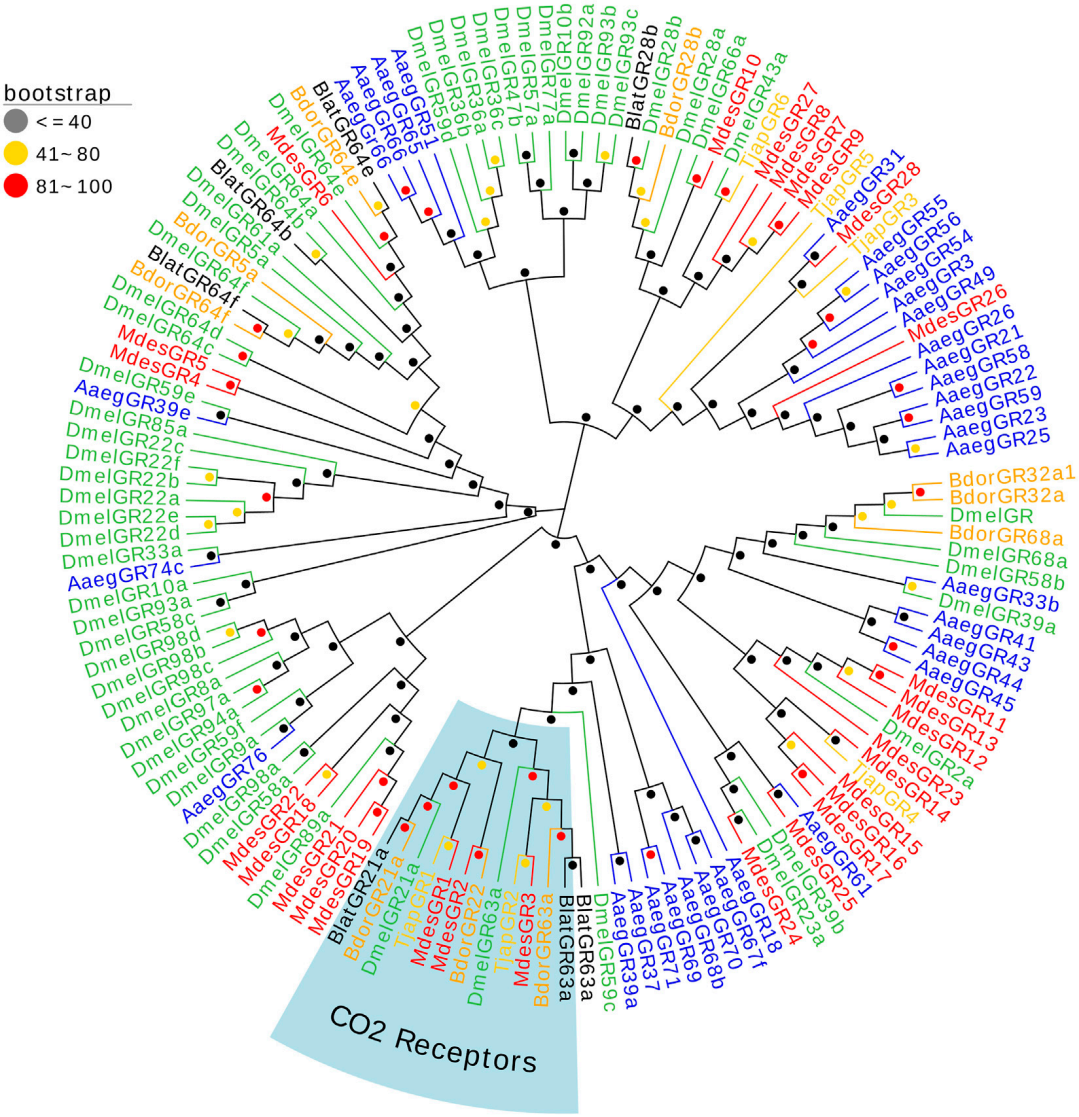
The NJ phylogenetic analysis of GRs in *T. japonensis*, *B. dorsalis*, *A. aegypti*, *M. de-structor*, *B. latifrons*, and *D. melanogaster*. The CO_2_ receptors group is shown.

Six Gustatory receptors of *T. japonensis* in the antennal transcriptomes were identified. Two TjapGRs (*TjapGR1/2*) have complete ORFs containing 7 TMDs, and four genes have partial ORFs in the 5′or 3′regions (Supplementary Table S4). Except that the FPKM value of *TjapGR1* was 401, the FPKM values of other TjapGRs were less than 10. Phylogenetic tree was constructed from GR sequences of five species of dipteran insects (*B. dorsalis*, *A. aegypti*, *M. destructor*, *B. latifrons*, and *D. melanogaster*) (Figure 4). The *TjapGR1* and *TjapGR2* gathered with CO_2_ receptors of other dipteran insects to form CO_2_ receptor branch.

#### 2.3.5 Ionotropic receptors (IRs)

**FIGURE 5:**
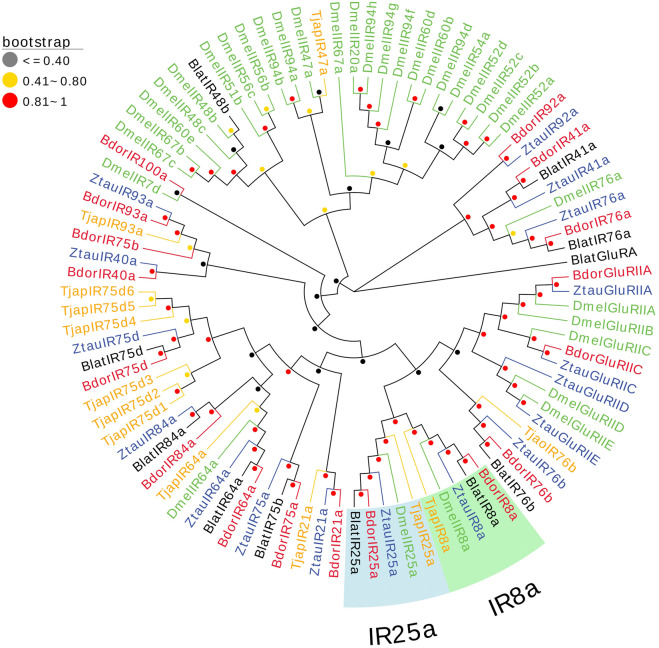
The NJ phylogenetic analysis of IRs in *T. japonensis*, *B. dorsalis*, *Zeugodacus tau*, *B. latifrons*, and *D. melanogaster*. The *TjapIR8a* group and TjapIR25a group are shown.

13 IRs in the antennal transcriptomes of *T. japonensis* were identified, and six IRs have complete ORFs ranging from 457 aa to 935 aa in length. Seven genes had partial ORFs in the 5′or 3′regions (Supplementary Table S5). The FPKM values of all IRs were greater than 1. *TjapIR75d5* and *TjapIR75d2* were highly expressed, with FPKM values of 46.44 and 78.13, respectively. A phylogenetic tree was established using the TjapIRs of *T. japonensis* with IRs gene sequences from other Dipteran insects, including *B. dorsalis*, *Zeugodacus tau*, *B. latifrons*, and *D. melanogaster*. *TjapIR8a* and *TjapIR25a* were clustered into conserved branches of IR8a and IR25a, respectively. Besides, *TjapIR21a* and *TjapIR76b* also formed IR21a and IR76b branches with homologous genes of other insects (Figure 5).

#### 2.3.6 Sensory neuron membrane proteins (SNMPs)

**FIGURE 6:**
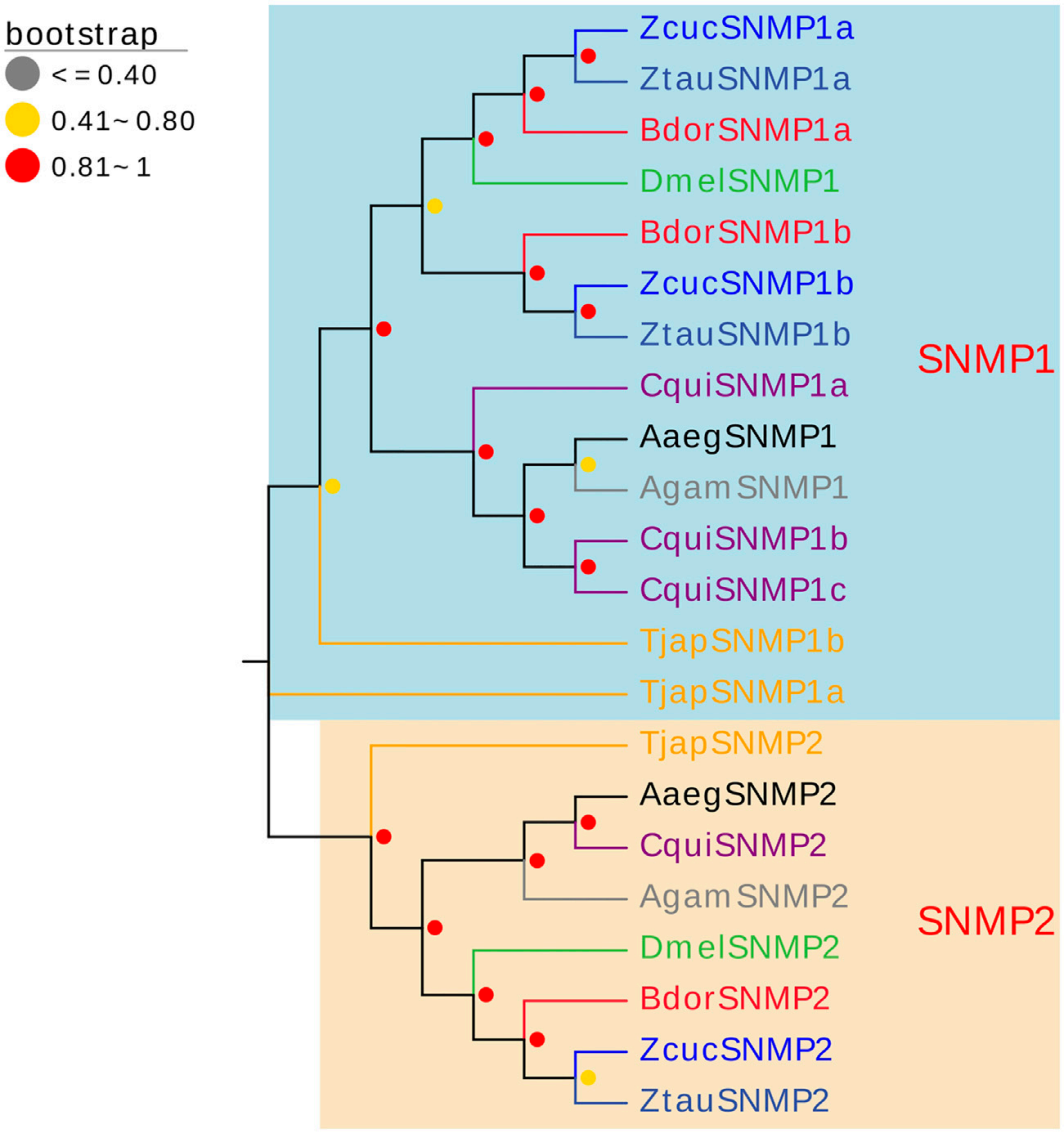
The NJ phylogenetic analysis of SNMPs in *T. japonensis*, *A. aegypti*, *A. gambiae*, *B. dorsalis*, *C. quinquefasciatus*, *Zeugodacus tau*, *Z. cucurbitae*, and *D. melanogaster*. The SNMP1 group and SNMP2 group are shown.

Three SNMPs of *T. japonensis* in the antennal transcriptomes were identified, encoding proteins ranging from 228 aa to 493 aa in length. Two TjapSNMPs (*TjapSNMP1a/TjapSNMP1b*) have complete ORFs, and *TjapSNMP2* have partial ORFs in the 3′regions (Supplementary Table S6). The FPKM values of all SNMPs were greater than 1. *TjapSNMP1b* was highly expressed, with FPKM values of 30.72. In the phylogenetic analysis of TjapSNMPs with proteins from other dipteran species, including *A. aegypti*, *A. gambiae*, *B. dorsalis*, *C. quinquefasciatus*, *Zeugodacus tau*, *Z. cucurbitae* and *D. melanogaster*, all SNMPs were separated into SNMP1 and SNMP2 groups with a high bootstrap support value (Figure 6).

### 2.4 Tissue expression profile analysis

**FIGURE 7:**
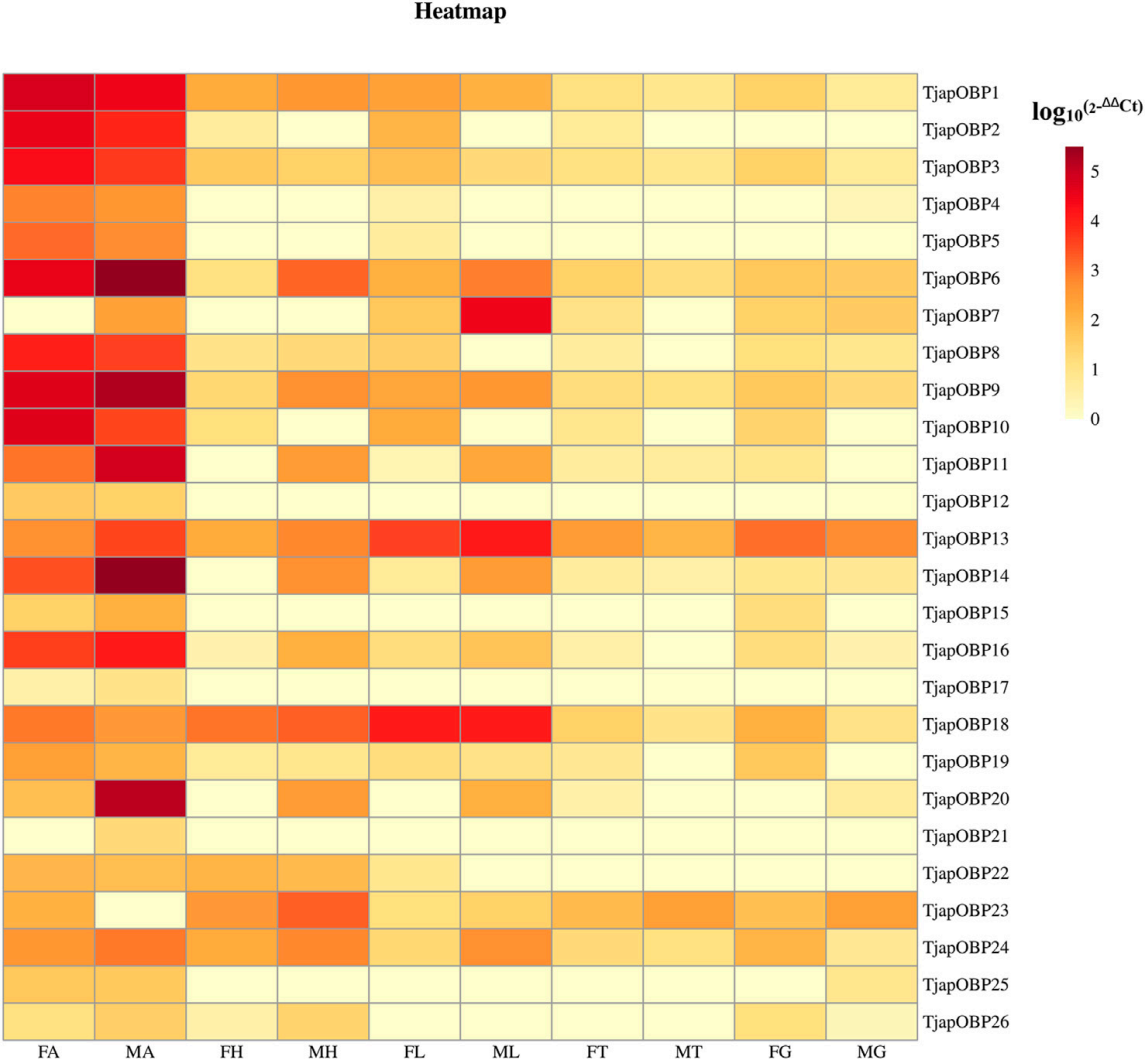
Relative expression levels of all TjapOBPs in different tissues of females and males. FA, female antennae, MA, male antennae, FH, female heads (without antennae), MH, male heads (without antennae), FT, female thoraxes, MT, male thoraxes, FG, female external genitals and MG, male external genitals. Note: the heatplot figure was built using the data set of RT-qPCR.

**FIGURE 8:**
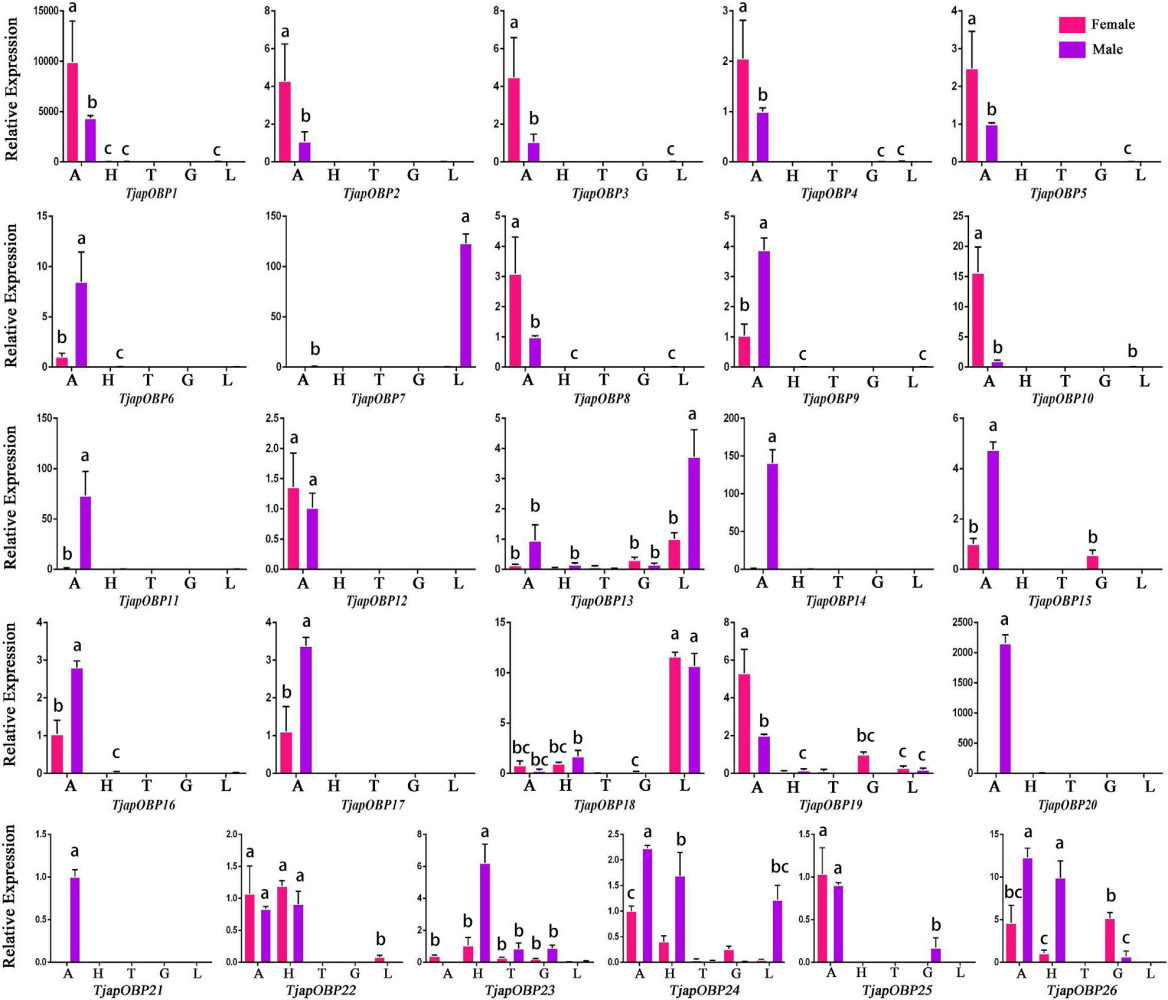
Expression profiles of *T. japonensis* OBPs in different tissues of female and male. A, antennae; H, heads (without antennae); T, thoraxes; G, external genitals; L, legs. The letters above the error bar denote significant differences (*p* < 0.05).

**FIGURE 9:**
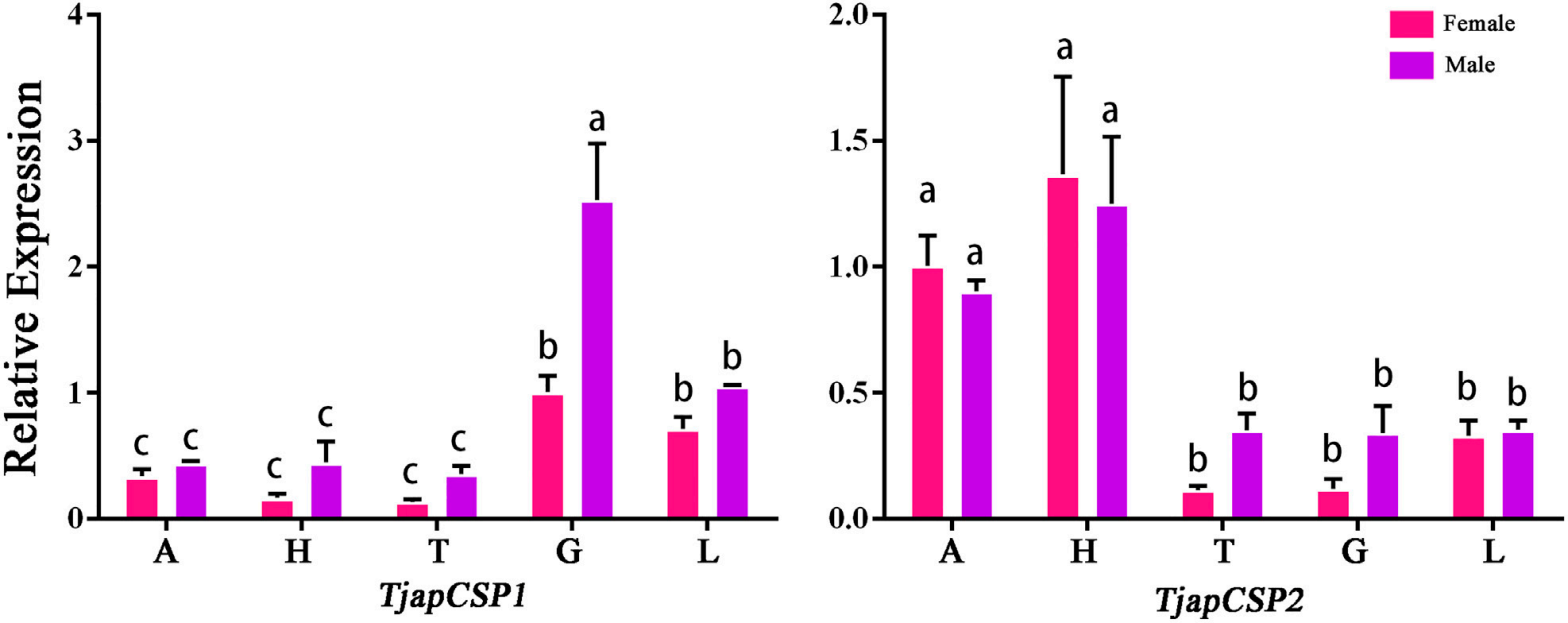
Expression profiles of *T. japonensis* CSPs in different tissues of female and male. A: antennae; H: heads (without antennae); T: thoraxes; G: external genitals; L: legs. The letters above the error bar denote significant differences (*p* < 0.05).

**FIGURE 10:**
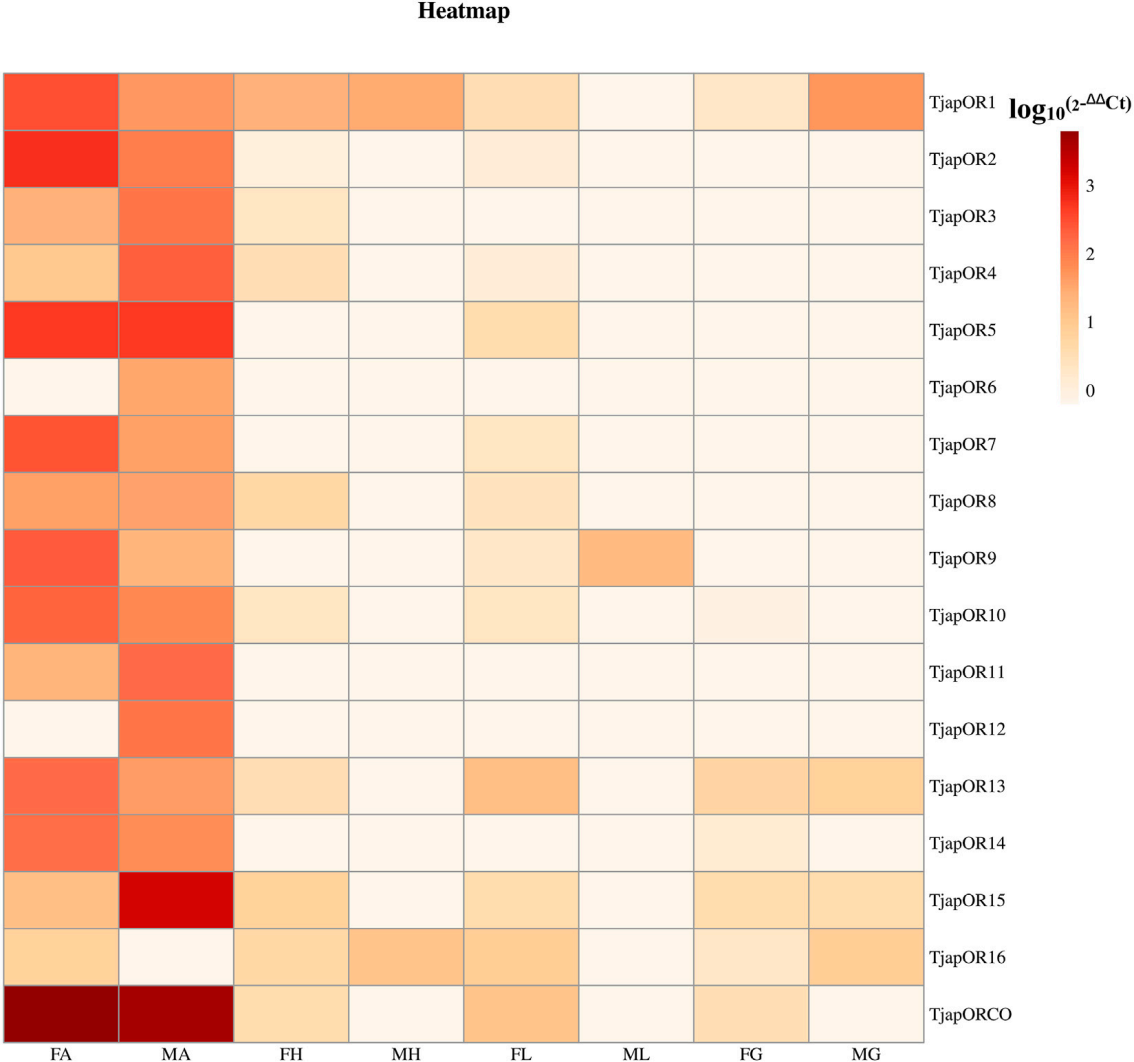
Relative expression levels of all TjapORs in different tissues of females and males. FA: female antennae, MA: male antennae, FH: female heads (without antennae), MH: male heads (without antennae), FT: female thoraxes, MT: male thoraxes, FG: female external genitals and MG: male external genitals. Note: the heatplot figure was built using the data set of RT-qPCR.

**FIGURE 11:**
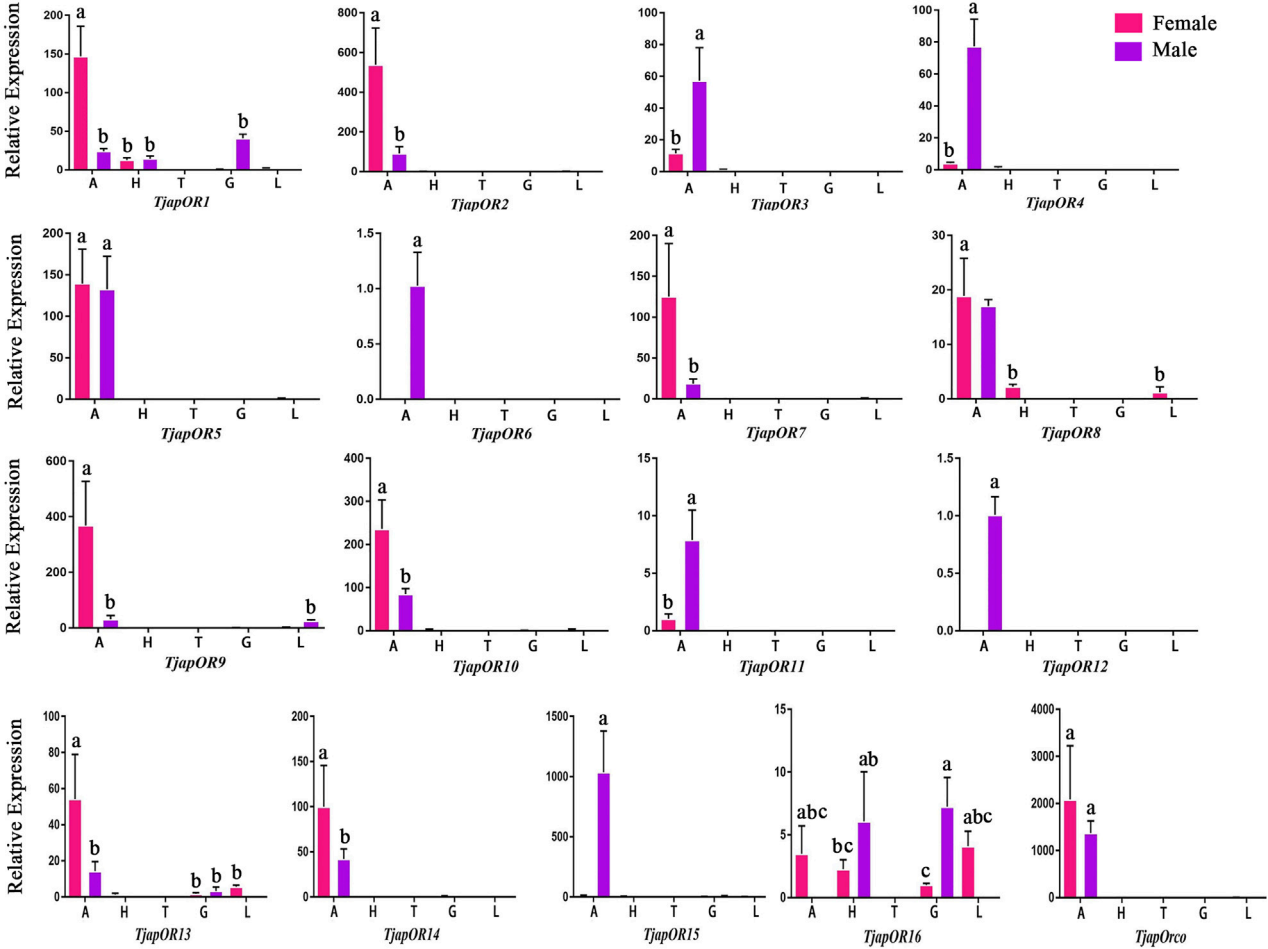
Expression profiles of *T. japonensis* ORs in different tissues of female and male. A: antennae; H: heads (without antennae); T: thoraxes; G: external genitals; L: legs. The letters above the error bar denote significant differences (*p* < 0.05).

A total of 26 OBPs, 2 CSPs and 17 ORs were used to explore the expression level in female antennae (FA), male antennae (MA), female heads (without antennae, FH), male heads (without antennae, MH), female thoraxes (FT), male thoraxes (MT), female external genitals (FG) and male external genitals (MG) by RT-qPCR. Genorm and NormFinder results showed that GAPDH and RPL3 have the same stability. Therefore, these two genes alone, or in combination, could be used for RT-qPCR analysis of gene expression in *T. japonensis* under our experimental conditions. We chose GAPDH as reference gene in the study. RT-qPCR results showed that 7 OBPs (*TjapOBP1/2/3/6/8/9/10*) were highly expressed in the antennae of females, and 6 OBPs (*TjapOBP1/6/9/11/14/20*) were highly expressed in male antennae (Figure 7). Most TjapOBPs (*TjapOBP1/2/3/4/5/6/8/9/10/11/12/14/16/17/20/21*) were biased expressed in the antennae of females and males. Among them, *TjapOBP6*, *TjapOBP11*, *TjapOBP14*, *TjapOBP15*, *TjapOBP20* and *TjapOBP21* were only expressed in male antennae (Figure 8). Besides, *TjapOBP18* was highly expressed in the legs of both females and males, while *TjapOBP7* and *TjapOBP13* displayed male-biased expression in the legs. *TjapOBP22* was highly expressed in female and male heads (Figure 8). In addition, *TjapCSP1* was highly expressed in the external genitalia of female and male adults, and *TjapCSP2* was highly expressed in the antennae of both female and male adults (Figure 9). RT-qPCR results showed that most ORs were highly expressed in the antennae of both females and males (Figure 10). *TjapORco* was the highest expressed ORs in antennae of male and female adults. *TjapOR5* was relatively highly expressed in the antennae of both female and male adults, while *TjapOR15* displayed male-biased high expression in the antennae (Figure 11).

## 3 Discussion

In the present study, we sequenced and analyzed the transcriptomes of antennae of adult *T. japonensis* (female and male), and searched for chemosensory-related genes. In total, we identified 26 OBPs, 2 CSPs, 17 ORs, 3 SNMPs, 6 GRs, and 13 IRs in the transcriptomes of *T. japonensis* antennae. As the tissue expression profile of chemosensory genes indicates its potential biological function, which could help to reveal the olfaction mechanism of insects (He et al., 2011; Gu et al., 2015; Yuan et al., 2015). We performed RT-qPCR to analyze the tissue expression profiles of candidate OBPs, CSPs and ORs. Most OBPs are highly expressed in insect antennae. Antenna-specific OBPs play an important role in recognizing host volatiles and pheromone (Gong et al., 2014; Brito et al., 2016). In this study, 16 of the 26 OBPs were biased expressed in antennae (Figure 8), indicating that they may be involved in recognizing odorant molecules. Phylogenetic analysis showed that *TjapOBP8* and *TjapOBP9* were clustered with *DmelOBP76a*/LUSH, a pheromone-binding protein bound to the sex pheromone of *D. melanogaster* (Ha and Smith, 2006). In addition, *TjapOBP1*, *TjapOBP2* and *TjapOBP3* were clustered with *DmelOBP83a/83b*, which is involved in the recognition of sex pheromone of *D. melanogaster* (Shanbhag et al., 2001; Siciliano et al., 2014). Therefore, we speculate that these proteins (*TjapOBP1/2/3/8/9*) might be involved in the sex pheromone recognition process of *T. japonensis*. These putative sex pheromone recognition-related OBPs were expressed in both unmated female and male antennae. The *TjapOBP1/2* were expressed more in female than male antennae (Figure 7). Amarawardana found that more sex pheromones were collected from fewer virgin females than much virgin females, when collecting sex pheromones of pear leaf midge (*Dasineura pyri*) and blackcurrant midge (*D. tetensi*) using an air entrainment method in a glass tube (Amarawardana, 2009). Therefore, it is hypothesized that a virgin female gall midge may sense the content of sex pheromone in the surrounding environment using its antennae to regulate its own release of sex pheromone. Laboratory tests with *T. japonensis* demonstrated the existence of sex pheromone in the virgin adult female, to which males responded with the typical sexual behaviour of raising the antennae and vibrating the wings (Lee and Lee, 1985). The life span of adults *T. japonensis* is very short, sometimes only 1–2 days, and do not feed. Adult males emerge, take flight, and copulate with females. Females emerge with a full complement of mature eggs, and search for host plants on which to oviposit after mating (Duan et al., 2021). Thus, efficient mechanism for finding mates is required. In this process, pheromone-binding proteins and pheromone detection related ORs are required to function.

Although most OBPs are biased and expressed in antennae, OBPs expressed in non-antennae also play an important role in the gustatory or olfactory sense (Shanbhag et al., 2001; Siciliano et al., 2014). For example, the *HarmOBP10*, expressed in the gland of the *Helicoverpa armigera*, was related to the synthesis, storage and release of pheromones (Sun et al., 2012). Two OBPs (*DmelOBP57d* and *DmelOBP57e*) expressed in the legs of *D. melanogaster* played a crucial role in host plant localization (Yasukawa et al., 2010). *AcerOBP15* was specifically expressed in the legs of *Apis cerana cerana* and was involved in taste recognition while collecting nectar and pollen (Du et al., 2019). The *PxyOBP13*, expressed in the head of the diamondback moth, *Plutella xylostella*, contributed to enhancing its resistance to pyrethroids (Bautista et al., 2015). In this study, RT-qPCR results showed that three OBPs (*TjapOBP7/13/18*) were highly expressed in the leg of *T. japonensis*, we speculate they may play a role in recognition of volatile or non-volatile compounds of host plants. The *TjapOBP22* was highly expressed in the head of *T. japonensis*, which may be related to taste function. The *TjapOBP26* was highly expressed in the female external genitalia of *T. japonensis*, we speculate it may be related to the synthesis and transport of sex pheromone.

Compared to OBPs, CSPs were more conserved, with 40%–50% homology between different insect species (Pelosi et al., 2006). The CSPs were not only expressed in antennae but also widely expressed in other parts of the body, such as heads, thoraxes, wings, ovaries, testes, legs and abdomens (Jacquin-Joly et al., 2001; Gong et al., 2012; Wang et al., 2017; Pelosi et al., 2018). Its functional diversity corresponds to the wide distribution of CSPs in insects. For example, the *SexiCSP3* of female *Spodoptera exigua* can significantly affect egg hatching (Gong et al., 2012). The *SinfCSP19* of *Sesa-mia inferens* displayed high binding affinities to both host plant volatiles and female sex pheromones (Zhang et al., 2014). In this study, we found that *TjapCSP1* was highly expressed in the external genitalia of females and males, and *TjapCSP2* was highly expressed in both female and male antennae. We speculate that the two CSPs may have other physiological functions.

Previous studies have shown that most ORs genes of insects were highly expressed in antennae, which played a crucial role in recognizing odorant molecules in the peripheral olfactory system of antennae (Montagne et al., 2015; Fleischer et al., 2018). For example, *DmelOR67d* has been proven to be the receptor of the *D. melanogaster* sex pheromone (Laughlin et al., 2008). For *H.armigera*, *HarmOR42* has been shown to specifically recognize phenylacetaldehyde, the main volatile of angiosperm floral scent, and was involved in finding host plants (Guo et al., 2021). The *LmigOR35* was a specific receptor for 4-vinylanisole, a polypheromone of locusts (Guo et al., 2020). We identified 17 ORs in the antenna transcriptomes of *T. japonensis*. RT-qPCR results showed that most ORs were highly expressed in the antennae of both unmated females and males. Phylogenetic analysis of ORs showed that *TjapORco* was clustered in a highly conserved clade with other Dipteran insects. The Orco is a widely expressed and highly conserved odorant receptor co-receptor that interacts with general odorant receptors to detect external odorants in insects (Benton et al., 2006; Yan et al., 2017; Fandino et al., 2019). RT-qPCR results showed that *TjapORco* was highly expressed in the antenna of unmated male and female adults. The olfactory function of *TjapORco* can be further confirmed using the EAG response assays and RNAi technology. *TjapOR4/5/7/14* were clustered with *DmelOR67d* of *D. melanogaster*, and *TjapOR5* was relatively highly expressed in the antennae of both female and male adults, we speculate that these ORs may have the same function as *DmelOR67d* in the process of sex pheromone recognition. The aforementioned research can provide a reference for the functional verification of TjapORs.

Many studies revealed that the IRs genes of insects are highly expressed in their antennae and play an important role in chemical molecule sensing, auditory function, temperature and humidity sensing (Benton et al., 2009; Senthilan et al., 2012; Zhang et al., 2013; Knecht et al., 2017; Liu et al., 2018; Greppi et al., 2020). IR8a and IR25a are the two most conserved IRs, considered co-receptors in IRs (Abuin et al., 2011). The phylogenetic tree of Diptera IRs showed that *TjapIR8a* and *TjapIR25a* clustered on the branches of IR8a and IR25a, respectively. Phylogenetic analysis of GRs showed that *TjapGR1* and *TjapGR2* were clustered together with *DmelGR21a* and *DmelGR63a* of *D. melanogaster*, respectively. Previous studies have shown that the co-expression of *DmelGR21a* and *DmelGR63a* regulates CO_2_ detection in *D. melanogaster* (Jones et al., 2007). SNMP1 is supposed to be related to the perception of pheromones in insects (Jin et al., 2008; Vogt et al., 2009; Li et al., 2014). The functions of three SNMPs identified from the antennal transcriptome of *T. japonensis* need further study.

In conclusion, this is the first report on the identification, classification and functional analysis of chemosensory-related genes in the antennal transcriptome of the economically important forest pest *T. japonensis*. A total of 67 chemosensory-related genes were identified, including 26 OBPs, 2 CSPs, 17 ORs, 3 SNMPs, 6 GRs, and 13 IRs. They contribute a significant contribution to the Diptera insect genomic database. In addition, tissue expression profile and phylogenetic analysis showed that some of these genes might be involved in physiological processes such as host recognition, mate location and spawning site. This work provides a theoretical basis for the study of the binding and sensing mechanism of the key olfactory genes *via* assessing protein expression, molecular docking, fluorescence binding competition, and RNA interference, which would facilitate the development of highly effective attractants and key gene silencing to control and monitor *T. japonensis*.

## 4 Materials and methods

### 4.1 Sample collection

Adult *T. japonensis* were collected in Qingdao, Shandong Province of China. During the emergence period in the middle of June, adults for transcriptome sequencing were collected in the infested *P. thunbergii* forest using a net. Besides, soil containing the pupae was put in cages (30 cm × 30 cm × 30 cm) under room temperature to collect unmated adults. Calling females and unmated males were collected in cages. The collected adults were immediately placed in liquid nitrogen, then taken back to the laboratory and placed in the −80°C. Antennae were quickly separated using high-precision tweezers (ideal-tek 5.SA, Switzerland) under the microscope and immediately put into a 1.5 mL centrifuge tube immersed in liquid nitrogen. Nearly 600 adult antennae were collected as a biological replicate for transcriptome sequencing. Two biological repeats were assessed for adult antennae.

### 4.2 RNA extraction

Total RNA was extracted from antennae for transcriptome sequencing by M5 HiPer Insects RNeasy Mini Kit (Mei5 Biotechnology, China) following the manufacturer’s instruction. The Qubit 2.0 fluorometer (United States, Thermo Fisher) was used to detect the concentration of RNA. The purity of RNA was detected by a nanodrop spectrophotometer (United States, Thermo Fisher). The integrity of RNA was evaluated with Agilent 2100 Bioanalyzer (United States, Agilent Technologies) and 1% agarose gel electrophoresis.

### 4.3 Transcriptome sequencing, assembly and function annotation

The cDNA library construction and transcriptome sequencing were conducted at a commercial company (China, Majorbio). An Illumina TruseqTM RNA sample prep Kit (United States, San Diego) was used to construct a cDNA sequencing library. The cDNA library was sequenced on Illumina Novaseq 6000 (United States, Illumina, San Diego) with double-ended reads of 150 bp in length. There were two biological replicates, and each biological replicate was sequenced three times. Fastp v0.19.5 was used to prune and control the quality of raw double-ended reads to produce clean and high-quality reads. Trinity v2.8.5 (https://github.com/trinityrnaseq/trinityrnaseq) was used for *de novo* transcriptome assembly. TransRate v1.0.3 (http://hibberdlab.com/transrate/) and CD-HIT v4.5.7 (http://weizhongli-lab.org/cd-hit/) were used to further filter sequences (Li and Godzik, 2006; Smith-Unna et al., 2016). BUSCO program v3.0.2 was used for assembly assessment (Simao et al., 2015). The assembled transcripts were annotated in six databases (NR, Pfam, GO, KEGG, COG, and Swiss-Prot) with an E-value threshold < 1e^−5^.

### 4.4 Identification of chemosensory-related genes

Putative OBPs, CSPs, ORs, IRs, GRs, and SNMPs were identified by tBLASTn program from the antennae transcriptome of *T. japonensis*. The candidate gene sequences were further confirmed by BLASTX in NCBI (https://www.ncbi.nlm.nih.gov). The open reading frame (ORF) and conserved domain were predicted in NCBI (https://www.ncbi.nlm.nih.gov/orffinder/). Expression levels were displayed as FPKM values (fragments per kilobase per million reads) by RSEM (Li and Dewey, 2011). After performed amino acid sequence alignment using the Muscle method (Edgar, 2004), the phylogenetic trees of all chemosensory-related protein sequences were constructed by the Neighbor-Joining method (Bootstrap 1000) in MEGA 7.0 software (Kumar et al., 2016). The sequences of olfactory genes used to build phylogenetic trees were listed in Supplementary Material S1. The phylogenetic trees were visualized by EvolView v2 (https://www.evolgenius.info/evolview-v2/).

### 4.5 Tissue expression profile analysis

The tissue expression profiles of candidate OBPs, CSPs and ORs were performed by RT-qPCR with CFX96 thermocycler (United States, BIO-RAD). The antennae, heads (without antennae), legs, thoraxes, and external genitals were collected from unmated gall midges and every 25 female or male adults for each biological replicate. And three biological replicates were used for each tissue. Total RNA was extracted from different tissues using Trizol reagent (Invitrogen, United States) following the manufacturer’s instruction. A Maxima First Strand cDNA Synthesis Kit (United States, Thermo Fisher) was used to synthesize the first strand of cDNA before a RapidOut DNA Kit (United States, Thermo Fisher) was used to remove the gDNA. Primers for RT-qPCR were designed by Primer3 (https://bioinfo.ut.ee/primer3-0.4.0/). The amplification efficiency of primers was calculated by the following equation: E = [10ˆ (−1/slope) −1] × 100%, in which the slope was derived by plotting the cycle threshold (Ct) value against five 3-fold serial dilutions. Only primers with 90%–110% amplification efficiency were used for RT-qPCR (Primers used for RT-qPCR were listed in Supplementary Material S1). The RT-qPCR reactions were executed in a 20 µL reaction mixtures, containing 0.4 µL of each forward and reverse primer (10 µM), 1 µL of cDNA, 10 μL of 2× ChamQ Universal SYBR qPCR Master Mix (Vazyme Biotech, China), and 8.2 µL of Nuclease-free water (USA, Thermo Fisher). The RT-qPCR condition was as follows: denaturation at 95°C for 30 s, then 95°C for 10 s and 60°C for 30 s (40 cycles). Melting curve analysis (65°C–95°C) was used to validate the specificity of primers. GAPDH and RPL3 (ribosomal protein L3) were selected as candidate reference genes from the antennal transcriptome. Using RT-qPCR pre-experiments, the genes with the most stable expression were tested as internal reference genes in http://blooge.cn/RefFinder/. Relative expression was performed by the 2^−ΔΔCt^ method with the reference gene. The expression differences between tissues were analyzed by Tukey’s honest significant difference test using SPSS software. GraphPad Prism 6 was used to construct figures. Heatplot figures of TjapOBPs and TjapORs were built in https://www.omicstudio.cn/tool/4.

## Data Availability

The datasets presented in this study can be found in online repositories. The data presented in the study are deposited in the repository: https://www.ncbi.nlm.nih.gov/, accession number: SAMN33231029-SAMN33231034.
