# Supplementary material for: Identification and expression profile analysis of chemosensory genes in pine needle gall midge, Thecodiplosis japonensis (Diptera: Cecidomyiidae)
Source: Front Physiol. 2023 Feb 16;14:1123479. doi: 10.3389/fphys.2023.1123479 (PMC9978445; doi:10.3389/fphys.2023.1123479)
Supplement: Supplementary file 1 [file DataSheet1.ZIP › Supplementary Figure 1.docx]

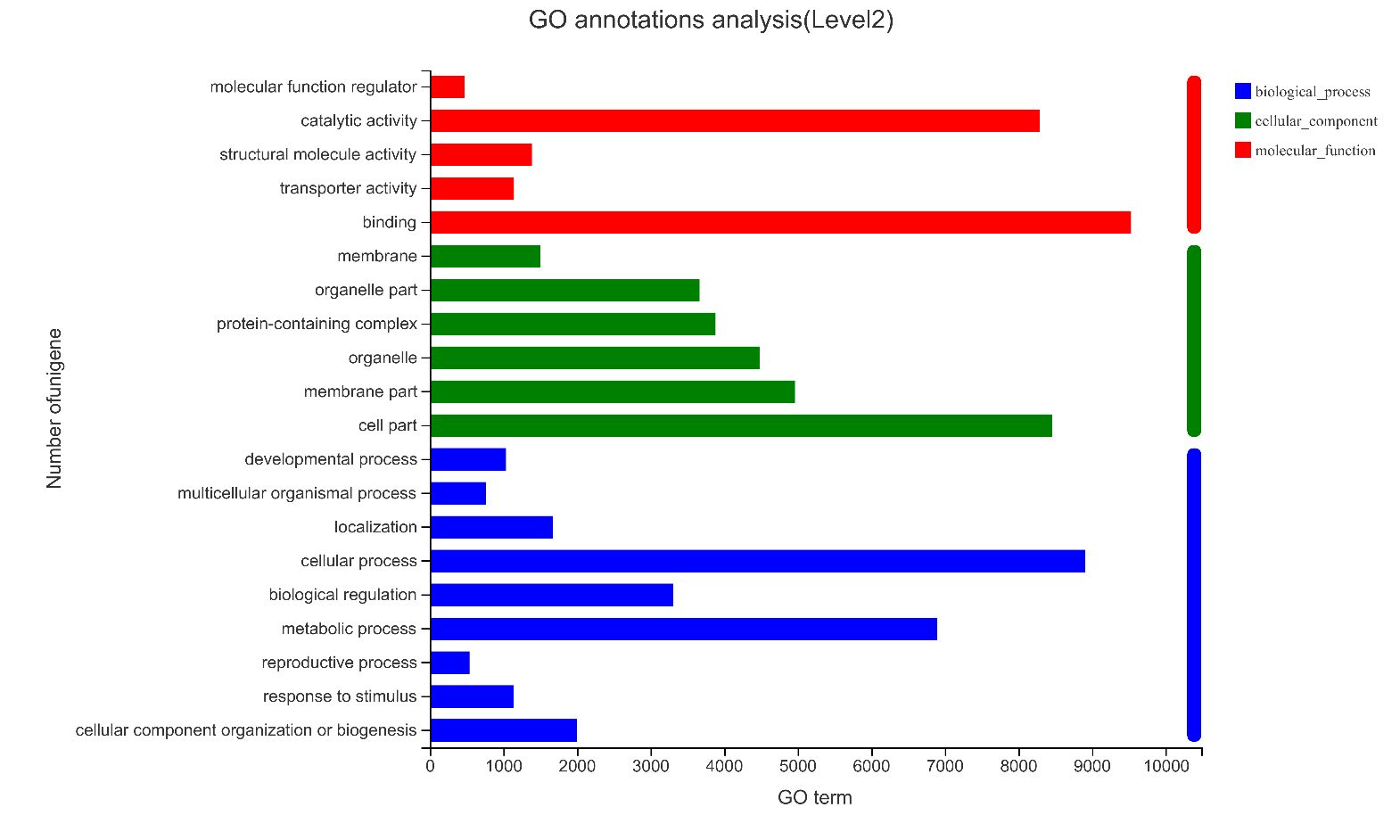


Supplementary Figure 1. Gene ontology (GO) classification showed by the quantity of *T. japonensis* transcripts
