# Supplementary material for: Identification and expression profile analysis of chemosensory genes in pine needle gall midge, Thecodiplosis japonensis (Diptera: Cecidomyiidae)
Source: Front Physiol. 2023 Feb 16;14:1123479. doi: 10.3389/fphys.2023.1123479 (PMC9978445; doi:10.3389/fphys.2023.1123479)
Supplement: Supplementary file 1 [file DataSheet1.ZIP › Supplementary File 2.docx]

Supplementary File 2

| TjapOBP1  TTCATGATGATCCTGCCTTG  TTCTCCGACGGGATACAAAC | TjapOBP2  GAGGCAGGGGTTGTTGACGA  ATACCAGAAGGCGCGCTCAC |
| --- | --- |
| TjapOBP3  ATCAGTATCCTCCGCCACAA  TGAAAGATTCATGCCAACCA | TjapOBP4  TTAACAAAACCGATGCACGA  GAAGATCCGCAACTCTGTCC |
| TjapOBP5  TGAAATGCCAAGCCGATATT  ACATCATCAAAGTGCGATGC | TjapOBP6  GGCCAATTCCACGTAGCTGGG  GCACGGTATAGGCAGCTTCACA |
| TjapOBP7  TGCACGCGTATTGAGCCGTGA  TTCACCAATCGCACGGGCTTCC | TjapOBP8  GGTTGACTCGGCTCTTATCG  AATGCACCGAGAGCAAATTC |
| TjapOBP9  TCGTCGAAAACAACAGTCCA  ATGCCATCAACCAATTCCTC | TjapOBP10  ATGGGTATGATGCGGAAAAG  AGCAGCTTCACATGGGTTCT |
| TjapOBP11  TGCATTGCGTCGCAGAAAACATGC  CGCCATTCGCCGCATCCTTACA | TjapOBP12  GCTATGCTCAGTGCGTTTTG  GAATGCAGCATCACAAGCAT |
| TjapOBP13  ATTCGTGCCAATCCAAAGAG  TGACCGATCTGCATGATTTC | TjapOBP14  CCAGATCACTTGCGAGCACCGA  TGCTTGCCTCGATATGTCGCTCC |
| TjapOBP15  ACTGCGAGCGAAAAATGTCT  CACAACGTTCAGGATGGCTA | TjapOBP16  AGCGATTGCAAAGAAAAGGA  GCTCTCCTATCCGCACTGAA |
| TjapOBP17  AAGCTCAGGAAAAAGCCACA  ACATCCGCTTTCAAACCATC | TjapOBP18  TGCAATATGGATTCGCTCAA  AACCGGCTCTTTCTTCATCA |
| TjapOBP19  AACAGTTGGCTTGGTTTCCA  TTTACAGCCAGCTCACAACG | TjapOBP20  TCGCGCGCAAAGTTCCAAGC  GCCAACTCAACTGCTCTGTCCACG |
| TjapOBP21  TGATGTTGAAGGCACCGTTA  TTTTGCATTACTGCGACCAC | TjapOBP22  TCGATCATAAAGCGTGTGGA  TGATATGGTATCGGGGCAAT |
| TjapOBP23  CTTCCCCAATGACGAGAAAA  CCGTTGGTCTTTTGTGGATT | TjapOBP24  TGGAAAATTCTCGGTCGAAG  ACAGCAGCATTAAGCAAGCA |
| TjapOBP25  TGAGCAACAACCAGCATCTC  CATTGCGATGCTTAAATGGA | TjapOBP26  TAAGGCAAGCATTGATGGTG  CCACGAGCTTTATGTGCTTG |
| GAPDH  TCACTTGGCTGGCGGTGCAA  TGGTGCCAAGCAGTTGGTGGTG | TjapORco  AGTTCATCGGTCATGGAAGC  TTGAGTTGCACCAACACCAT |
| TjapCSP1  TGGGAATTGTATGCATGGTG  AGCTCACGACCTTCACGAGT | TjapCSP2  TCGTCAGTCGGCAAATAATG  GGGATATCGCGTCTGTAGGA |
| TjapOR1  CGAGGTGGCTATGGCTTTAT  ATCGGTTTCAAGGGTCCTCT | TjapOR2  TGCGGCCTCTTTGTTATTCT  AAGCCATACCCATGCTGAAC |
| TjapOR3  CTCGAAAACGAATCGGAAAC  CAGCCGGATAGTCCATCACT | TjapOR4  AGCGCGAGCAGAAAAAGTAT  TACTTCAGCGAATGCAGCAG |
| TjapOR5  GGATAGATCGCTTTTGCAG  AATCGCATTGAACCCTGAAC | TjapOR6  GTGTACGCCGGAGTTTTGTT  AATCGAAACCGTTGTTCCAG |
| TjapOR7  CACGTAAAGTGCGAATCACG  GCACATTCCATTTCATGCAG | TjapOR8  CAATCGTTCGGAAGTGTGTG  TGTGTTTCGAACGCTTCAAC |
| TjapOR9  CAGGCAATGAATCGTTTGTG  ACGCCAACATGAACTCACAG | TjapOR10  TTGCGCATTTGATGAGAATC  TTGCGTTTCTGTCGTACCTG |
| TjapOR11  GCCGCTCTATTGGGATTGTAT  ACAGAATCCGAAACCAGCAC | TjapOR12  GGTGTCGCCGCTAGTACAGT  TCAGCCAACAGAACATCGAG |
| TjapOR13  GCATTTGGTGTGTTCATGAGCG  CCATCCACAGAGAGATTCAGTGC | TjapOR14  TTCACTTGATATGGCGCAAC  TAACAGCGCAAACAGCAAAC |
| TjapOR15  ATGGGGTGGTGCTACTGTGT  GCACCGCTTACTTCTTCACC | TjapOR16  GTTATTTGGCGGCAATCTGT  ACCAAACGCAACGAGAAATC |
