# Supplementary material for: Identification and expression profile analysis of chemosensory genes in pine needle gall midge, Thecodiplosis japonensis (Diptera: Cecidomyiidae)
Source: Front Physiol. 2023 Feb 16;14:1123479. doi: 10.3389/fphys.2023.1123479 (PMC9978445; doi:10.3389/fphys.2023.1123479)
Supplement: Supplementary file 1 [file DataSheet1.ZIP › Supplementary File1.docx]

| Supplementary File1 | | | |
| --- | --- | --- | --- |
| The sequences used to build phylogenetic trees of olfactory genes | | | |
|  | | | |
| IRs | | | |
| Accession number | Name | Accession number | Name |
| Q9VCM0 | DmelIR94h | A1ZAY9 | DmelIR 54a |
| Q9VCM1 | DmelIR 94g | A1ZA17 | DmelIR 52d |
| Q8IMY8 | DmelIR 94f | A1ZA16 | DmelIR 52c |
| B7Z0P2 | DmelIR 94d | A1ZA15 | DmelIR 52b |
| Q8IN09 | DmelIR 94b | A1ZA14 | DmelIR 52a |
| Q8IN10 | DmelIR 94a | A1Z9Y5 | DmelIR 51b |
| B7Z0Y1 | DmelIR 7d | A1Z8P2 | DmelIR 48c |
| Q9VW39 | DmelIR 76a | A1Z8N9 | DmelIR 48b |
| Q8IQE2 | DmelIR 67c | A1Z882 | DmelIR 47a |
| Q9VT95 | DmelIR 67b | Q9VRI8 | DmelIR 20a |
| Q9VT09 | DmelIR 67a | Q9VCM0 | DmelIR 94h |
| Q9W155 | DmelIR 60e | Q9VCM1 | DmelIR 94g |
| B7YZQ6 | DmelIR 60d | Q9VT09 | DmelIR 67a |
| B7YZQ4 | DmelIR 60b | Q9W365 | DmelIR 8a |
| A1ZBM8 | DmelIR 56c | Q9VRL4 | DmelIR 64a |
| A1ZBM7 | DmelIR 56b | M9PC56 | DmelIR 25a |
| A0A0G2UKD7 | BdorIR76b | A0A0G2UEM7 | BdorIR 100a |
| A0A348AZW9 | BdorIR 25a | A0A0G2UES7 | BdorIR 84a |
| A0A6M9TYZ3 | BdorIR 40a | A0A0G2UGG3 | BdorIR 92a |
| A0A6M9TYZ4 | BdorIR 8a | A0A0G2UMS0 | BdorIR 75d |
| A0A6M9TZC8 | BdorIR 64a | A0A348AZY5 | BdorIR 75b |
| A0A0G2UEM3 | BdorIR 41a | A0A6M9TZA0 | BdorIR 21a |
| A0A6M9TZA1 | BdorIR 76a | A0A5H2WZ74 | BlatIR76b |
| A0A6M9TZB8 | BdorIR 75a | A0A5H2X049 | BlatIR 8a |
| A0A6M9TZI6 | BdorIR 93a | A0A5H2X5R2 | BlatIR 25a |
| A0A5H2WZ47 | BlatIR 41a | A0A5H2WVZ7 | BlatIR 75d |
| A0A5H2X081 | BlatIR 64a | A0A5H2WWJ3 | BlatIR 76a |
| A0A5H2X4Q1 | BlatIR 48b | A0A6M9TZK7 | ZtauIR64a |
| A0A5H2X5S7 | BlatIR 75b | A0A6M9TZL7 | ZtauIR 40a |
| A0A5H2XA15 | BlatIR 84a | A0A6M9TZU0 | ZtauIR 8a |
| A0A6M9TZX6 | ZtauIR 75d | A0A6M9TZU2 | ZtauIR 76b |
| A0A6M9TZM1 | ZtauIR 41a | A0A6M9TZV6 | ZtauIR 25a |
| A0A6M9TZM7 | ZtauIR 93a | A0A6M9TZR6 | ZtauIR 92a |
| A0A6M9TZN0 | ZtauIR 76a | A0A6M9TZV9 | ZtauIR 84a |
| A0A6M9TZQ6 | ZtauIR 21a | A0A6M9U083 | ZtauIR 75a |
| A0A6M9U096 | ZtauGluRIID | A0A0K8VB57 | BlatGluRA |
| A0A6M9U0J9 | ZtauGluRIIE | A0A6M9TZS6 | BdorGluRIIC |
| A0A6M9TZM8 | ZtauGluRIIA | A0A6M9TZL1 | BdorGluRIIA |
| A0A6M9TZM2 | ZtauGluRIIC | Q0KI42 | DmelGluRIIE |
| Q9VPV3 | DmelGluRIIC | Q9TVI0 | DmelGluRIID |
| Q24351 | DmelGluRIIA | O45028 | DmelGluRIIB |
|  |  |  |  |
| SNMPs | | | |
| Accession number | Name | Accession number | Name |
| A0A6M9TYN0 | ZcucSNMP2 | E1JI63 | DmelSNMP2 |
| A0A6M9TYP7 | ZcucSNMP1a | Q9VDD3 | DmelSNMP1 |
| A0A6M9TYQ5 | ZcucSNMP1b | C3U0S3 | AaegSNMP2 |
| A0A6M9TZQ5 | ZtauSNMP1a | Q17A88 | AaegSNMP1 |
| A0A6M9TZW7 | ZtauSNMP1b | Q7Q6R1 | AgamSNMP2 |
| A0A6M9TZZ2 | ZtauSNMP2 | Q7QC49 | AgamSNMP1 |
| A0A6M9TZD8 | Bdor SNMP1a | G0YYB0 | CquiSNMP1a |
| A0A6M9TZL3 | Bdor SNMP1b | G0YYB1 | CquiSNMP 1b |
| A0A6M9TZN2 | Bdor SNMP2 | G0YYB3 | CquiSNMP 2 |

| GRs | | | |
| --- | --- | --- | --- |
| Accession number | Name | Accession number | Name |
| P83293 | DmelGR64a | P58962 | DmelGR 58a |
| Q9V4K2 | DmelGR 43a | P58985 | DmelGR 59d |
| Q9VKA5 | DmelGR 33a | P83292 | DmelGR 23a |
| Q9VM08 | DmelGR 28b | P83294 | DmelGR 64b |
| Q9VPT1 | DmelGR 21a | P83295 | DmelGR 64c |
| Q9VSH2 | DmelGR 66a | P83296 | DmelGR 64e |
| Q9VZL7 | DmelGR 63a | P84180 | DmelGR 22b |
| Q9W497 | DmelGR 5a | P84181 | DmelGR 22d |
| P58958 | DmelGR 39a | Q8IMN5 | DmelGR 98d |
| P83297 | DmelGR 64f | Q8IMN6 | DmelGR 98c |
| Q9VD76 | DmelGR 93a | Q8IMQ6 | DmelGR 97a |
| Q9VKJ7 | DmelGR GR | Q8IMZ5 | DmelGR 94a |
| Q9VTN0 | DmelGR 68a | Q8IN23 | DmelGR 93b |
| Q9VZJ6 | DmelGR 64d | Q8IN58 | DmelGR 92a |
| Q9W1U5 | DmelGR 59c | Q8INZ2 | DmelGR 36c |
| Q9W367 | DmelGR 8a | Q8IPU5 | DmelGR 77a |
| P58950 | DmelGR 10a | Q8IRL8 | DmelGR 9a |
| P58951 | DmelGR 22a | Q9V969 | DmelGR 57a |
| P58952 | DmelGR 22c | Q9VB26 | DmelGR 98b |
| P58953 | DmelGR 22e | Q9VB26 | DmelGR 98a |
| P58954 | DmelGR 22f | Q9VD74 | DmelGR 93c |
| P58955 | DmelGR 36a | Q9VJF2 | DmelGR 36b |
| P58960 | DmelGR 39b | Q9VM09 | DmelGR 28a |
| P58961 | DmelGR 47b | Q9W0M2 | DmelGR 61a |
| Q9VEU0 | DmelGR 89a | Q9W1N5 | DmelGR 59f |
| Q9VEU0 | DmelGR 10b | Q9W1N6 | DmelGR 59e |
| Q9W2B2 | DmelGR 58c | Q9W2B1 | DmelGR 58b |
| Q8INM9 | DmelGR 85a | Q9W594 | DmelGR 2a |
| A0A0K8U1J7 | BlatGR63a | A0A5H2X5P1 | BlatGR 64b |
| A0A0K8WM41 | BlatGR 28b | A0A5H2WZ23 | BlatGR 64e |
| A0A5H2WXV9 | BlatGR 63a | A0A5H2X4M7 | BlatGR 64f |
| A0A5H2WZ00 | BlatGR 21a | A0A0G2UMR5 | BdorGR 32a |
| A0A0G2UEL7 | BdorGR28b | A0A6M9TZ21 | BdorGR 64e |
| A0A0G2UES1 | BdorGR 68a | A0A6M9TZC4 | BdorGR 5a |
| A0A0G2UGF3 | BdorGR 21a | A0A6M9TZL5 | BdorGR 22 |
| A0A0G2UKD2 | BdorGR 63a | A0A348AZW4 | BdorGR 64f |
| J9HIQ5 | AaegGR66 | J9E9W6 | Aaeg44 |
| J9HIX7 | Aaeg67f | J9EA20 | Aaeg37 |
| J9HSS4 | Aaeg39e | J9EA67 | Aaeg61 |
| J9HT02 | Aaeg18 | J9EAD7 | Aaeg51 |
| J9EAQ2 | Aaeg54 | J9EAL2 | Aaeg68b |
| J9HZB8 | Aaeg69 | J9EBE2 | Aaeg58 |
| J9HIA2 | Aaeg33b | J9EBH6 | Aaeg74c |
| J9HZE7 | Aaeg49 | J9EBL7 | Aaeg70 |
| J9HIE0 | Aaeg43 | J9HFQ6 | Aaeg39a |
| J9I030 | Aaeg71 | A0A1S4G5W6 | Aaeg66 |
| Q16JW6 | Aaeg59 | A0A6I8TQX6 | Aaeg65 |
| Q16JW7 | Aaeg21 | Q16TD0 | Aaeg56 |
| Q16JW8 | Aaeg22 | Q16TD1 | Aaeg55 |
| Q16JW9 | Aaeg26 | Q16TD2 | Aaeg3 |
| Q16JX0 | Aaeg25 | Q16VJ1 | Aaeg76 |
| Q16JX1 | Aaeg23 | Q170E8 | Aaeg31 |
| Q175X6 | Aaeg45 | Q175X4 | Aaeg41 |

| ORs | | | |
| --- | --- | --- | --- |
| Accession number | Name | Accession number | Name |
| P81909 | DmelOR22a | P82982 | DmelOR 65a |
| P81910 | DmelOR 22b | P82983 | DmelOR 65b |
| P81912 | DmelOR 23a | P82984 | DmelOR 65c |
| P81917 | DmelOR 43a | P82985 | DmelOR 69a |
| Q9VHQ7 | DmelOR 85b | P82986 | DmelOR 82a |
| Q9VNB5 | DmelOR Orco | Q8IRZ5 | DmelOR 19b |
| Q9VT92 | DmelOR 67d | Q9I816 | DmelOR 19a |
| O46077 | DmelOR 2a | Q9V568 | DmelOR 45a |
| P81914 | DmelOR 33a | Q9V589 | DmelOR 45b |
| P81915 | DmelOR 33b | Q9V6A9 | DmelOR 49a |
| P81916 | DmelOR 33c | Q9V6H2 | DmelOR 49b |
| P81918 | DmelOR 43b | Q9V8Y7 | DmelOR 56a |
| P81921 | DmelOR 47a | Q9V9I4 | DmelOR 42b |
| P81923 | DmelOR 59a | Q9VAW0 | DmelOR 98b |
| Q9V3N2 | DmelOR 46a | Q9VU27 | DmelOR 71a |
| Q9V3Q2 | DmelOR 35a | Q9VVF3 | DmelOR 74a |
| Q9V9I2 | DmelOR 42a | Q9VXL0 | DmelOR 13a |
| Q9VNB3 | DmelOR 83a | Q9VYZ1 | DmelOR 10a |
| Q9VT08 | DmelOR 67a | Q9VZW8 | DmelOR 63a |
| Q9W1P8 | DmelOR 59b | Q9W1P7 | DmelOR 59c |
| Q9W5G6 | DmelOR 1a | Q9W2U9 | DmelOR 9a |
| P81911 | DmelOR 22c | Q9W3I5 | DmelOR 7a |
| P81913 | DmelOR 24a | Q9VFN2 | DmelOR 88a |
| P81922 | DmelOR 47b | Q9VHE6 | DmelOR 85f |
| P81924 | DmelOR 85e | Q9VHQ2 | DmelOR 85d |
| Q9VAZ3 | DmelOR 98a | Q9VHQ6 | DmelOR 85c |
| Q9VCS8 | DmelOR 94b | Q9VHS4 | DmelOR 85a |
| Q9VCS9 | DmelOR 94a | Q9VLE5 | DmelOR 30a |
| Q9VDM1 | DmelOR 92a | Q9VNK9 | DmelOR 83c |
| Q9VT90 | DmelOR 67c | Q9VT20 | DmelOR 67b |
| A0A6M9TXZ7 | BcorOR7a | A0A6M9TY59 | BcorOR 10a |
| A0A6M9TY20 | BcorOR 19a | A0A6M9TY71 | BcorOR 46a |
| A0A6M9TY22 | BcorOR 43a | A0A6M9TY89 | BcorOR 94b |
| A0A6M9TY23 | BcorOR 42a | A0A6M9TYC1 | BcorOR 13a |
| A0A6M9TY32 | BcorOR 49b | A0A6M9TYD8 | BcorOR 47b |
| A0A6M9TY36 | BcorOR 63a | A0A6M9TYE0 | BcorOR Orco |
| A0A6M9TY38 | BcorOR 59a | A0A6M9TYG4 | BcorOR 49a |
| A0A6M9TY80 | BcorOR 67d | A0A6M9TYK4 | BcorOR 45a |
| A0A6M9TY48 | BcorOR 69a | A0A6M9TYL8 | BcorOR 67c |
| A0A6M9TY50 | BcorOR 74a | A0A6M9TYM9 | BcorOR 92a |
| A0A6M9TYZ7 | BcorOR 88a | A0A3G2LEL6 | BminOR 85e |
| A0A3G2LEI1 | BminOR7a | A0A3G2LEL8 | BminOR 85b |
| A0A3G2LEI3 | BminOR 22c | A0A3G2LEM1 | BminOR 2 |
| A0A3G2LEI4 | BminOR 2a | A0A3G2LEL4 | BminOR 88a |
| A0A3G2LEI5 | BminOR 10a | A0A3G2LEL9 | BminOR 85d |
| A0A3G2LEI7 | BminOR 33a | A0A3G2LEM0 | BminOR 6 |
| A0A3G2LEI9 | BminOR 24a | A0A3G2LEM2 | BminOR 4 |
| A0A3G2LEJ0 | BminOR 45a | A0A3G2LEM5 | BminOR 7 |
| A0A3G2LEJ2 | BminOR 47b | A0A3G2LEM6 | BminOR 5 |
| A0A3G2LEJ4 | BminOR 35a | A0A3G2LEM8 | BminOR 8 |
| A0A3G2LEJ6 | BminOR 43a | A0A3G2LEM9 | BminOR 3 |
| A0A3G2LEJ7 | BminOR 63a | A0A3G2LEN6 | BminOR 9 |
| A0A3G2LEJ9 | BminOR 49a | A0A6H1V5Y2 | BminOR 12 |
| A0A3G2LEJ8 | BminOR 45a | A0A6N0A5X5 | BminOR 16 |
| A0A3G2LEK3 | BminOR 74a | A0A7D4V5V3 | BminOR 24 |
| A0A3G2LEK4 | BminOR 67d | A0A3G2LEJ5 | BminOR 46a |
| A0A3G2LEK5 | BminOR 67c | A0A3G2LEK6 | BminOR 82a |
| A0A3G2LEK7 | BminOR 71a | A0A3G2LEL1 | BminOR 1 |
| A0A3G2LEL0 | BminOR 94a | A0A3G2LEL3 | BminOR ORCO |
| A0A0G2UES8 | BdorOR59a | A0A6M9TYV4 | BdorOR 19a |
| A0A0G2UEX4 | BdorOR 49b | A0A6M9TYX2 | BdorOR 7a |
| A0A0G2UEY4 | BdorOR 94b | A0A6M9TYY8 | BdorOR 92a |
| A0A0G2UGJ8 | BdorOR 43a | A0A6M9TZ65 | BdorOR 10a |
| A0A0G2UGK6 | BdorOR 67d | A0A6M9TZ97 | BdorOR 94a |
| A0A0G2UKH6 | BdorOR 45a | A0A0G2UMW0 | BdorOR 43b |
| A0A348AZT7 | BdorOR 82a | A0A6M9TZB0 | BdorOR 88a |

>TjapCSP1

MKSTFAMFLLLGIVCMVIAEKYPDKYDQVDIDNVLNNDRVLTNYIKCLLGKGACTREGRELKKLLPDALQTDCSKCSVVQKRNSKKVINFLRTRRPNDWKNLVAKFDPEGLFKQRLDAGLA

>TjapCSP2

MNTKMRQTICGVLLITLLSNTHNYGAHADDKTINRLLNNQAIVSRQIMCVLEKSPCDQLGRQLKAALPEVILRNCRNCSPQQAQNAQKLTNFLQTRYPDVWAMLIRKYRGV

>DmelCSP1

MKASLALVFCVCVGLAAAAPEKTYTNKYDSVNVDEVLGNNRVLGNYLKCLMDKGPCTAEGRELKRLLPDALHSDCSKCTEVQRKNSQKVINYLRANKAGEWKLLLNKYDPQGIYRAKHEGH

>DmelCSP2

MKMILALVVLGLVLVAAEDKYTTKYDNIDVDEILKSDRLFGNYFKCLVDNGKCTPEGRELKKSLPDALKTECSKCSEKQRQNTDKVIRYIIENKPEEWKQLQAKYDPDEIYIKRYRATAEASGIKV

>DmelCSP3

MGQPGFRRAIGHVSLVVALMCTTCFQVEGLPHPPATSPSPMMERMVEQAYDDKFDNVDLDEILNQERLLINYIKCLEGTGPCTPDAKMLKEILPDAIQTDCTKCTEKQRYGAEKVTRHLIDNRPTDWERLEKIYDPEGTYRIKYQEMKSKANEEP

>DmelCSP4

MLLLNKNRVISLVVNFIFLIILISSSVQADERNINKLLNNQVVVSRQIMCILGKSECDQLGLQLKAALPEVITRKCRNCSPQQAQKAQKLTTFLQTRYPDVWAMLLRKYDSA

>AgamCSP1

MKLFVVVALALVAAVAAQDKYTSKYDNINVDEILKSDRLFGNYYKCLLDQGRCTPDGNELKRILPDALQTNCEKCSEKQRDGAIKVINYLIQNRKDQWDVLQKKFDPENKYLEKYRGQAQKEGIKLD

>AgamCSP2

MKLFVAIAFALLALAAAQEQYTTKYDGIDLDEILKSDRLFNNYFKCLMDEGRCTPDGNELKKILPEALQTNCEKCSEKQRSGAIKVINYVIENRKEQWDALQKKYDPENLYVEKYREEAKKEGIKLE

>AgamCSP3

MKFFVVVALALVAAVAAQDKYTTKYDGVDLDEILKSDRLFNNYYKCLMDTGRCTPDGNELKRILPDALKTDCAKCSEKQKSGTEKVINYLIDNRKDQWENLQKKYDPENIYVNKYREDAKKKGINL

>AgamCSP4

MERFLLLLLFVAIVLGETANETYVTKYDNIDLEEIFSSKRLMDNYMNCLKNVGPCTPDGRELKDNLPDALMSDCVKCSEKQRIGSDKVIKFIVANRPDDFAILEQLYDPTGEYRRKYMQSDALAEHVKQEDRDLSSSGDGDADTETEAHATEHNSQDHDHREGQSDAE

>AgamCSP5

MRKVWLLASVVLAFLDFVKSQEVARTLYSTRYDNLDIDTILASNRLVTNYVDCLLSRKPCPPEGKDLKRILPEALRTKCARCSPIQKENALKIITRLYYDYPDQYRALRERWDPSGEYHRRFEEYLRGLQFNQIGGSNGGSGVGNTVLSNL

>AgamCSP6

MKHLTMVAIFAMVVVLASAQKYTDKFDNIDVDRVLSNDRILNNYLKCLLDKGPCTQEGRELKKTLPDALKTNCEKCSEKQRTSSRKVIAHLEERKPQEWKKLLDKYDPEGIYKSKFEKINKRS

>AgamCSP7

MLSAAVIVVMAALVIVGPQPAAANDSQNINRLLNNQVIVSRQIMCVLEKSPCDQLGRQLKAALPEVIQRNCRNCSPQQAQNAQKLTNFLQTRYPEVWAMLIRKYGAV

>AgamCSP8

MLHNLFLSLSLYVSVCGDPSGSTCAAEATTARTQVSDEALDKALSDKRYLMRQLKCALGEVACDPVGKRLKSLAPFVLRGACPQCTPAEMNQIKKTLAHLQRNFPSEWNKLVQTYAG

>AsinCSP1

MKLIVFIAFALVATVAAQQYTTKYDNIDVDEILKSDRLFNNYYKCLLDEGRCTPDGNELKRILPDALQTDCAKCSEKQRSGAIRVLNYLIQNRPTQWAVLQKKYDPENQYVEKYREQAKKEGIKLD

>AsinCSP2

MKLFVAIAFALLAIVAAQEQYTTKYDGIDLDEILKSDRLFNNYFKCLMDEGRCTPDGNELKKILPEALQTNCAKCSEKQRAGAIQVINYVIENRKEQWDALQKKYDPENLYIEKYREEAKKEGIKLE

>AsinCSP3

MKFFVVVALAMVAAVAAQEKYTTKYDGVDLDEILKSDRLFNNYYKCLLDQGRCTPDGNELKRILPDALQTDCAKCSEKQKSGTEKVINYLIDNRNDQWQNLQKKYDPENIYVNKYRDEANKKGIKL

>AsinCSP4

MLPLVLVVMAVMVLGSMADNYVTKYDNINLEEIFNSSRLMNNYMNCLKNVGPCTPDGKELKNNLPDALMSDCVKCSEKQRIGSDKVIKFIIANRPDDFATLEQLYDPTGEYRRKYLAPDGTLKPREDGEEDVPPVKETNDGDIEIDSVAHATEHKTAPSQDHDHGEGHTDESKN

>AsinCSP5

MRKVWLLASAVLAFLNFVKSQEVARTLYSARYDNLDIDTILGSNRLVSNYVDCLLSRKPCPPEGKDLKRILPEALRTKCARCSPIQKENALKIITRLYFDYPDQYRALRERWDPSGEYHRRFEDYLRGLQFNQIGGNGATNGGQAPSGGDNGNTVVENGGGNDRPVRNDLDRQPASQSSNVQAVVIDPTLSSGGRQRPNDPGPRHR

>AsinCSP6

MKNLSIVAVLAMLVVLVSAQKYTSKFDDIDVDRVLSNDRILNNYLKCLLDKGPCTQEGRELKKTLPDALKTNCEKCSEKQRTSSRKVIAHLEDRKPQEWKKLLDKYDPEGIYKSKFEKLNKRS

>AsinCSP7

MSSKALPNLFMLSAAVIALMAVLIAGPQPVVANDSQNINRLLNNQVIVSRQIMCVLEKSPCDQLGRQLKAALPEVIQRNCRNCSPQQAQNAQKLTNFLQTRYPEVWAMLIRKYGAV

>AsinCSP8

MSGKVSSRSSSSSICWFIGVGLCALVMVVQLAGLAEADATTTSRTQVSDEALDKALSDKRYLMRQLKCALGEVPCDPVGKRLKSLAPFVLRGACPQCTPAEMNQIKKTLAHLQRNFPSEWNKLVQTYAG

>CquiCSP1

MAVGVALAVLVLVLGGGQVAANDTQNLNRLLNNQVIVSRQIMCVLEKSPCDQLGRQLKAALPEVIQRNCRNCSPQQAQNAQKLTNFLQTRYPEVWAMLIRKYGAV

>CquiCSP2

MKSLVLSVLCLATLLVATTVAQQPRQYTDKFDNINVDQVLSNDRILSNYIRCLLDKGPCTQEGRELKKTLPDALRSNCEKCSEKQRNNSRKVISHLEAKKPADWKKLLDKYDPEGLYKSKFEKLNKRS

>CquiCSP3

MRNVWIVVASGLLAFANFVKSQETGRTLYSSRYDNLDIDTILSSNRLVNNYVDCLLSRKPCPPEGKDLKRILPEALRTKCGRCSSTQKENALKIITTLYYSYPDQYQALRERWDPSGEYHRRFEEYLRGIQFNQIGGNGNGNGGPSERPVRNDFDRDQSQILLQTLILSTTTAQPPVPEQRPTQLASAHSGGVSGDEAGGGSEPPTSIPATDSSPYIPLSGNPHKQ

>CquiCSP4

MFSNIVILGAVLLCCTLQAAVNGAEYDTKYDNVDLDEIFRSTRLLNNYMNCLKKVGPCTPEGKELKENLPDALANDCAKCSDKQKAGASKVIHFIVENRRDDFGALEKLYDPSGEFRRKYLDEQMHFRLHREEGGSAAVEEKSPASESEATTEEAAAAAQDHGQSAEGRR

>CquiCSP5

MKLYIVVALALIAAVAAQDKYTTKYDGIDLDEILKSDRLFNNYFKCLMDQGRCTPDGKELKRLLPDALNTNCSKCSEKQKQGTEKVVNYLIDNRPSQWKTLQEKYDPDNTYRTKYRVEAKKFGITV

>CquiCSP6

MKFFIVALALFALAVAQEDDGDKYTSKYDKIDLDDILGSDRLFKNYYNCLLDQGACTPEGNYLKRVLPEALETNCAKCTEKQDADSTKTIKYLSENRPAEWKVLKAKFDPENKYVEKYVDKAEKEGIKL

>CquiCSP7

MKFFIIALALFALAVAQEDDGDKYTSKYDKIDLDDILGSDRLFNNYYKCLLDQGPCTPEGNYLKRVLPEALETNCVKCTEKQDADSTKTIKYLSENRPAEWKVLKAKFDPENKYVEKYEDKAEKEGIKL

>CquiCSP8

MKFFIVALALVALVAAQEEEGDKYTTRYDNIDLDEILKSDRLFKNYYACLVEEGRCTAEGSYLKRILPEALETNCAKCSDKQRDDGVRAIKYMAENRAEEWKVLKARFDPENKYVEKYLADAEKEGIKL

>CquiCSP9

MKFFIVALALVALVAAQEEEGDKYTTKYDKIDLDDILKSDRLFKNYYACLLDDGPCTPEGSYLKRILPEALETNCAKCSDKQRDDGVRAIKYMAENRAEEWKVLKAKFDPENVYVEKYLADAEKEGIKL

>CquiCSP10

MKFFIVALALVALVAAQEEDGDKYTTRYDNIDLDEILKSDRLFKNYYNCLVDEGRCTAEGSYLKRILPDALETNCAKCSDKQRDDGVRAIKYMADNRAEEWKVLKARFDPENKYVEKYLADAEKEGIKL

>CquiCSP11

MKLFIVGLALFAVAFAQDAEVETLEEIGEKYTTKFDKIDLDDILKSDRLFKNYYNCLMEEGPCTPEGNYLKRVLPEALENSCNKCSEKQQKDSVKAIKYLTENRSEAWKVLKAKYDPENKYVEKYLTDADAEGIKL

>CquiCSP12

MKLIIVLALVALAAAQVPFTNKYDHINVEEILMSDRLFKNYFNCLIDEGACTPEASELKEKLPEALENNCELCTEKQKDTSVKVIRYLIDKRPVEWGVLKTKFDPNNKFVDRYREEAEAAGIKL

>CquiCSP13

MKLLIVFALVALVAAQDSTYTNKYDNIDVDEILKSDRLFKNYFNCLIDQGPCTPDATELKQSLPDALENNCSKCTPKQKEVGYKVVGWLINNRPEEWNVLRAKYDPENKFIEKYRDEAKAAGINL

>CquiCSP14

MKLLIVFALVALVAAQDSTYTNKYDHIDVDEILKSDRLFKNYYNCLIDQGPCTPDAAELKQSLPDALENNCSKCTPKQKETGYKVISSLIENRPAEWAVLQDKYDPERKFVEKYREEAAAAGIKL

>CquiCSP15

MKLLIAFALVALVAAQDSTYTNKYDHIDVDEILKSDRLFKNYYNCLIDQGPCTPDAAELKQSLPDALENNCSKCTPKQKETGYKVISSLIENRPAEWAVLQDKYDPERKFVEKYREEAAAAGIKL

>CquiCSP16

MKLLIAFALVALVAAQDSTYTNKYDHIDVDEILKSDRLFKNYYNCLIDQGPCTPDAAELKQSLPDALENNCSKCTPKQKETGYKVISSLIENRPAEWAVLQDKYDPERKFVEKYREEAAAAGIKL

>CquiCSP17

MKLLIVFALVALVAAQDSTYTNKYDHIDVEEILKSDRLFKNYYNCLIDQGPCTPDAAELKQSLPDALENNCSKCTPKQKETGYKVISSLIENRPAEWAVLQDKYDPERKFVEKYREEAAAAGIKL

>CquiCSP18

MKLFIVFALVALVAAQDNTYSSKYDNVDIDEILKTDRLFKNYYNCLIDQGPCTPDATELKQVLPDALENNCSKCTPKQKDAGYKVVGFLIDNRPEEWAVVRAKYDPENKFVEKYRGDAEAAGVKL

>CquiCSP19

MKTFIVFGLLALVAAQDSTYTNKYDHIDVEEILKSDRLFKNYYNCLIDQGPCTPDATELKQSLPDALENNCSKCTPKQKEVGNKVVAYLIESRPEEWAVLKAKFDPENKFVEKYREEAAAAGIKL

>CquiCSP20

MKFFVAFFALVALVAAQELYTNKFDTVDLDEILKSDRLFKNYYQCLLDEGRCTPDGNELKRVLPEALETNCAKCSEKQKTAGNKAFKYLAANRPTEWKALLAKFDPESKYTAKKLDIKIFIPFNY

>CquiCSP21

MKFFVAIFAALLAVAAAQELYTSKYDSVDVDEILKSDRLFKNYYQCLLEDGRCTPEGNELKRILPEALETNCAKCSEKQRSGAIKAFGYLSENRPEEWKALRARFDPENKYLEQYREEGEKNGIKF

>CquiCSP22

MKCVIVAVLALIALVSAQEAEQYTTKYDNIDLDEILKSDRLFNNYYKCLMDEGPCTPDGNELKRILPEALQTNCAKCSEAQRAGAIKVINHVIDNRPEQWKALQAKYDPENIYVEKYRTEAAEAGIAL

>CquiCSP23

MKYLIALALLIVAVAAQNKYTTKYDGIDLDEILKSDRLFNNYYKCLLEQPGGRCTPDANELKRILPEALQTNCAKCSKKQKDGAVKVINYLIDNRSAQWQVLQKKYDPENVYINQYRTEARAAGVKV

>CquiCSP24

MKCFIAFALLVVAVAAQNKYTTKYDGIDIDEILKSDRLFNNYYKCLLDQGRCTPDANELKRILPEALQTNCAKCTEKQKDGAVRVINYLIDNRSAQWQVLQKKFDPENVYINQYRNEARAAGIKV

>CquiCSP25

MYCKAVTTKMSSHQQQQQRRRHRTPVYCDQLASWLLLAVFAVSTVLLCCCVTSAQAQPQTPPTKSQVSDEALDKALSDKRYLMRQLKCALGEVPCDPVGKRLKSLAPFVLRGACPQCTATEMNQIKKTLAHLQRNFPQEWNKLVQTYAG

>CquiCSP26

MKFFIVALALFALAVAQEDDGDKYTSKYDKIDLDDILGSDRLFKNYYNCLLDQGACTPEGNYLKRVLPEALETNCAKCTEKQDADSTKTIKYLSENRPAEWKVLKAKFDPENKYVEKYVDKAEKEGIKL

>CquiCSP27

MIPSKYDKIDLDDILGSDRLFNNYYKCLLDQGPCTPEGNYLKRVLPEALETNCVKCTEKQDADSTKTIKFLSENRPAEWKVLKAKFDPENKYVEKYEDKAEKEGIKL

>AaegCSP1

MNTTKLVMLSATLIVALMVFNWPQPAAANDSQNLNRLLNNQVIVSRQIMCVLEKSPCDQLGRQLKGRSGVDF

>AaegCSP2

MKSVCLIVFGVVALVATVSAQQKYTDKFDNINVDQVLSNDRILSNYLKCLLEKGPCTQEGRELKKTLPDALRTNCEKCSEKQRTNSRKVISHLESKKPAEWKKLLDKYDPEGIYKSKFEKLNKRS

>AaegCSP3

MERKSTGCNPPQSSPPVLQTRLNRRAPSGEIPSDWDLEWQLFVPRIGIYPRTGKRGIVRCEKKIEREAKKPEENAVLKLNQCANKSGRYECELSSEQKKCFVRVASALLAFANFVKSQDSARNLYSSRYDNLDIDTILGSNRLVNNYVDCLLSRKPCPPEGKDLKRILPEALRTKCARCSVTQKENALKIITTLYYSYPDQYMALRERWDPSGEYHRRFEEYLQGIQFNQIGSNGNDRPVRNDFDRDQSQVLLQTLILSSTTVAPSSPPQPQPQSPNALPNGPEQRPQQLAPSAQLTQPAEATKKLE

>AaegCSP4

MSHKFCWIVVICAISIINVNCYDTKYDNVNLDEIFKSTRLLNNYINCLKNMGPCTPDAKELKELLPDALESECAHCTEKQKVGAERVINFVVDNRPDDFKILESMYDPAGEYRRKYLRDHPNFHDQGAPLTAADATENPPSSNGGDEAPTEESQQNQGQSEDGDDRRR

>AaegCSP5

MKLFAVVALALFAVAAAQEKYTTKYDGVDLDEILKSDRLFNNYYKCLMDQGRCTPDGNELKRVLPDALKTDCAKCSPKQRDGTQKVVNYLIDNRPSQWKNLQAKYDPQNIYVEKYRTEAKKAGIKL

>AaegCSP6

MKYFFVVFLALAATVIAQNEINQYTTKFDSIDVDEILKSDRLFNNYYKCLLDLGRCTPEGVELKRVLPEALETSCAKCSEKQRETSDRAIKYLTENRPEEWKVLKARYDPDNKYSKKNENDA

>AaegCSP7

MKLFIALALLAVAAAQEATYNNRYDNIDVEEILKSDRLFKNYFNCLMDAGPCTPEGTDLKKYLPDALETGCTKCTEKQRDTGNKVIAWLIENRPMEWVMLKSKYDPENKLTERYRELAAKAGIAL

>AaegCSP8

MKIIILCTLLAVVAAQEATYNNRYDNIDVEEILKSDRLFKNYFNCLMDAGPCTPEGTDLKKYLPDALETGCTKCTEKQRDTGNKVIAWLIENRPMEWTMLKNKYDPENKLTERYRELAAKAGIAL

>AaegCSP9

MKFLVAVLSLIAVAAAQDLYTTKFDNIDVDEILKSDRLFKSYYQCLLDEGRCTPEGNELKRSLPDALETGCSKCSEKQRSAGVRAVKYLSENRPTEFKALRNRFDPENKYVEQYVRDAEKEGITLNI

>AaegCSP10

MKIFIAVFTLMAVVAAQEFYTSKFDNIDVDEILKSDRLFKNYYQCLLDQGRCTPEGNELKRVLPDALETACSKCSEKQRSAGVRAVKYLSENRPAEFKALRARFDPENKYVDQYVRDAEKEGITLNIS

>AaegCSP11

MKFFVVALALIAAVAAQDEAMYTSKFDNINLDEILQSDRLFKNYYNCLTDAGPCTPEGNELKRVLPEALETNCAKCSPKQREAGTRAIKHVTENRPEEWKVLRARFDPENKYIERFSAEAEKEGIKL

>AaegCSP12

MDHSSISASKPSAHFKVLNRSFTHKFGATDTIMKIFVVALALIAAVAAQDEAMYTSKFDNINLDEILMSDRLFKNYYNCLTDAGPCTPEGNELKRVLPEALETNCAKCSPKQREAGTRAIKYVTENRAEEWKVLRARFDPEDKYVAQYLAEAEKEGIKL

>AaegCSP13

MKFFIVVLALFAVAAARPQEDKYTTKYDSIDIDEILKSDRLFKNYFNCLMDTGACTPEGNELKRVLPDSLENNCSKCSEKQQTSSTKIIKFLTENKPEEWTMLKAKYDPDNKYVQKYVADADKDGIKL

>AaegCSP14

MKFFIVALALFAAAAARPQEDKYTTKYDSIDIDEILKSDRLFKNYFNCLMDTGACTPEGNELKRVLPDSLENNCSKCSEKQQTSSTKIIKFLTENKPEEWTMLKAKYDPDNKYVQKYVADADKDGIKL

>AaegCSP15

MKFSIVVLALFAVAAAKPQDDKYTTKYDSIDIDEILKSDRLFKNYFNCLMDTGACTPEGNELKRVLPDSLENNCSKCSEKQQTSSTKIIKFLTENKPEEWTMLKAKYDPDNKYVQKYVADADKDGIKL

>AaegCSP16

MKFFIVVLALFAVAAARPQDDKYTTKYDSIDIDEILKSDRLFKNYFNCLMDTGACTPEGNELKRVLPDSLENNCSKCSEKQQTSSTKIIKFLTENKPEEWTMLKAKYDPDNKYVQKYVADADKDGIKL

>AaegCSP17

MKFFIVALVLIAVAAARPQDDKYTTKYDSIDIDEILKSDRLFKNYFNCLMDTGACTPEGNELKRVLPDSLENNCSKCSEKQQTSSTKIIKFLTENKPEEWTMLKAKYDPDNKYVQKYVADADKDGIKL

>AaegCSP18

MKFFIVALVLIAVAAARPQEDKYTTKYDSIDIDEILKSDRLFKNYFNCLMDTGACTPEGNELKRVLPDSLENNCSKCSEKQQTSSTKIIKFLTENKPEEWTMLKAKYDPDNKYVQKYVADADKDGIKL

>AaegCSP19

MKFFIVVLALFAVAAARPQEDKYTTKYDSIDIDEILKSDRLFKNYFNCLMDTGACTPEGNELKRVLPDSLENNCSKCSEKQQTSSTKIIKFLTENKPEEWTMLKAKYDPDNKYVQKYVADADKDGIKL

>AaegCSP20

MKFFIVALALIAVVAAQDDKYTTKYDSIDIDEILKSDRLFKNYFNCLMDTGACTPEGNELKRVLPDALENNCSKCSEKQQTSSTKIIKFLTENKPEAWTMLKAKYDPDNKYVAKYVADADKEGIKL

>AaegCSP21

MKFFIVALALIAVAAARPQDDKYTTKYDSIDIDEILKSDRLFKNYFNCLMDTGACTPEGNELKRVLPDALENNCSKCSEKQQTSSTKIIKFLTENKPEEWTMLKAKYDPDNKYVAKYVADADKKGIKL

>AaegCSP22

MKFFIVALALIAVAAAQDDKYTTKYDSIDIDEILKSDRLFKNYFNCLMDTGACTPEGNELKRVLPDALENNCSKCSEKQQTSSTKIIKFLTENKPEEWTMLKAKYDPDNKYVQKYVAEADKDGIKL

>AaegCSP23

MKFFIVALALFAVAAAQDDKYTTKYDSIDIDEILKSDRLFKNYFNCLMDTGACTPEGNELKRVLPDSLENNCSKCSEKQQTSSTKIIKFLTENKPEEWTMLKAKYDPDNKYVQKYVADADKDGIKL

>AaegCSP24

MKFFIVALALLAVVAAQDDKYTTKYDSVDIDEILKSERLFKNYYACLMDTGACTPDVNELKRVLPDALENNCAKCSEKQQNDSTKTIKYLTENKPEEWKALKAKYDPDNKYVEKYVADADKEGIKL

>AaegCSP25

MKIFIVALALIAVVAAQDDKYTTKYDSVDIDEILKSERLFKNYYACLMDTGACTPDVNELKRVLPDALENNCAKCSEKQQNDSTKTIKYLTENKPEEWKALKAKYDPDNKYVEKYVADADKEGIKL

>AaegCSP26

MKFFIFALALIALAAAKPQDDKYTTKYDSVDIDEILKSERLFKNYYACLMDTGACTPDVNELKRVLPDALENNCAKCSEKQQNDSTKTIKYLTENKPEEWKALKAKYDPDNKYVEKYVADADKEGIKL

>AaegCSP27

MKFFIVALALIALAAAKPQDDKYTTKYDSVDIDEILKSERLFKNYYACLMDTGACTPDVNELKRVLPDALENNCAKCSEKQQNDSTKTIKYLTENKPEEWKALKAKYDPDNKYVEKYVADADKEGIKL

>AaegCSP28

MKIFIVALALIALAAAKPQDDKYTTKYDSVDIDEILKSERLFKNYYACLMDTGACTPDVNELKRVLPEALENNCAKCSEKQQNDSTKTIKFLTENKPEEWKALKAKYDPDNKYVEKYVADADKEGIKL

>AaegCSP29

MKIFIVALALIALAAAKPQDDKYTTKYDSVDIDEILKSERLFKNYYACLMDTGACTPDVNELKRVLPEALENNCAKCSEKQQNDSTKTIKFLTENKPEEWKALKAKYDPDNKYVEKYVADADKEGIKL

>AaegCSP30

MKLFIVALALLAVVAAQDDKYTTKYDSVDIDEILKSERLFKNYYACLMDTGACTPDVNELKRVLPDALENNCAKCSEKQQNDSTKTIKYLTENKPEEWKALKAKYDPDNKYVEKYVADADKEGIKL

>AaegCSP31

MKIFIVALALIALAAAKPQDDKYTTKYDSVDIDEILKSERLFKNYYACLMDTGACTPDVNELKRVLPDALENNCAKCSEKQQNDSTKTIKYLTENKPEEWKALKAKYDPDNKYVEKYVADADKEGIKL

>AaegCSP32

MKIFILCAIMAVVAAQEATYNNRYDNIDVEEILKSDRLFKNYFNCLMDAGPCTPEGTDLKKYLPDALETGCTKCTEKQRDTGNKVIAWLIENRPMEWVMLKSKYDPENKLTERYRELAAKAGIAL

>AaegCSP33

MKLFIALALLAVAAAQEATYNSRYDNIDVEEILKSDRLFKNYFNCLMDAGPCTPEGTDLKKYLPDALETGCTKCTEKQRDTGNKVIAWLIENRPMEWVMLKSKYDPENKLTERYRELAAKAGIAL

>AaegCSP34

MKLFVAVFALIAVVAAQELYTSKFDNIDVDEILKSDRLFKNYYQCLMDEGRCTPEGNELKKILPEALETNCAKCSEKQRDGAIKAFGYLSENRPTEWKTLRDRFDPEGKYIEQYREEAEKNGIKF

>AaegCSP35

MKIMIVIAFALLAVASAQEEQYTTKYDNIDVEEILKSDRLFNNYFKCLMDEGPCTPDGNELKRILPEALQTNCAKCSESQRAGAIKVINYMIENRAEQWKALQEKYDPENIYLEQYRAEAEQSGITL

>AaegCSP36

MKSFIVIALALVVAVAAQNKYTSKYDGVDIDEILKSDRLFNNYYKCLLDQGRCTPDANELKRILPEALKTNCEKCSEKQREGATRVINYLIENRNQQWQTLQAKFDPENIYINQYRNEARAAGIKI

>AaegCSP37

MNSVNRYVLCIALIALFVASFTTAEENCEISANELGKIEQTLTHINQPIYTGDDESEVSDSDQCAQMLRGIHFQLRRLTQKYKLMNKGYVKAEEFAKMARDYEDQLSVLKNDLEQLKIGADSSAKQKMQELKKDIATLEQNVNTLHKDLEGITDELGKVRMDLCLTYMESNQLSNAQDKVKTLAPKYLMELVEQFLNKSEKNWLPVVDLSVAIPDLDDRGQVYKTVHEFLKTKNRDGGEDSILLEAEVLKMNATFHPGSKITEDRKKEIQDLLEKLSLTSTKIFDQWTQDLAKLENSAVYKNSIDRMFLTQMEKFGERVMAKDDYYSLRNFLKLLVVSTNYYKIAAYRKLIQEKIGHTLAVLMFDMMSMERTELQYDPHVPDEVVRMYDESITALPDSLKNIRSCLKLVKIYNHVTNQCILATNEVEDVNNSNPKFKSNVLGRRKLVKTASNDCTPFRLEPSADKASIRIITPKGDALTNINSIQPGLSWFNRVGAPYTNNHNMKLDYSADWILDANYANDSIKIESEFNAYQTMKSVDHLMVTNVGKVPHVVVAQYGLKGMEYAGAGMKDAEWKFKCDN

>AaegCSP38

MKLKVYICQVIFSFLAVSVFCEENCNIPESELSKIDHVLRHMEKPIYSEEQFASDNEECTNLLNGIHAQLRRLTQRYKLMNKGYVKVEEYQRMADDYEKQLKTLNDELVELQQHTSEKASATIAKLKEDIKKLDEEVGTLHEKLKGIKQDFEKVKRDLCVTYLNSNQMSKAKAKLKEMASTYLIEIVQQQLNKSNANIMPMLEFSAAIPDLDDMGEAYKEIYKFLEEQKRLEGEDSVLLEATVLKMNASLKEGSNITDERRTQIEGLLKDLATKSTIVFSTWTKELKKINDAVVIKNALDHMFVSQMKVFGALVGDTSDFGSIRNFVKLTVVCNNYYKVAAYKELIDRKIGNALGTIMFDLLTLEVNEMKFDPHVPDEIPKLFEATLSSLPNSLTELRTCLGKVQIYNKKTNKCVVATGNDFDVHKDKLGDFYRVVVADYGCTSFRLEASGDKASVRIVTPSGNPMSNVNLHLEGNSLHNYVATPKSNKPDRTPSSSDEWILDANYNNDTIKIESQFSDYKTKKTEVDHLLVRDINHLPHVLVARYGFMGLKNSDAKDTIEWNLKCGS

>AaegCSP39

MARGMGCWQPASQWGHKGFGFVTFQSEDVVDKVCEIHFHEINNKMVECKKAQPKEVMLPANLAKTRTAGRGTYDFMWSLGTLPDGFPAAAYAAYAAGRGFSGYPSFGLPYPTGNLNLAALHAHLAAAAATASAGGPHHHHHAHSSSSGNNTTPPPPTRSNPALVQLEATRIDCCLIPGGEHRTTYNSTNMHEMHFNPTSCRMRIVSLVGLVGSDRAYHPPPGTGAGESKLDKGIRHRSSIHSGAYTQLLCIRPNVCEGNYTRVDVAGEFVSTFPARFLDHYIVIVVVRRRSKALSSFDSTPPSTL

>AaegCSP40

MNRQLWIIIFAILCVAQAEEDNPTTEKMEELGIATINNFTREFYSYVEAVSQVLADLELTTTASITQIKHRIKHLLQEKCNLCSAKAEGPALDQGYVTTSNGSVIPVSYEQTRFGGGWIVLMQRYDGTVRFNRSWAEYRDGFGMVGHEFWLGLERIHQMTKDAEYELMIEMQDFEGNYKYAGYDAFAVGPEEERYPLAKVGKFNKTAYVDSFGKHRGYGFSTYDNDDNGCSNQYGRGGWWYYRKSCFGASLTGIWQNKQDWKSISWVWFSTEKKQVPLKFARMMMRLKTAE

>AaegCSP41

MILQFWVVTFSVLFAARADENHSILIKLNDLDHRFTQMFSQQFYRHTQQVTDRVSALKISIDTNLLELDQQIQQALDGIQSNESSSSASATKPPGLTTIPIGSEPRVPALYERERYGGDWLVVMHRYDGSVKFDRTWAEYRDGFGMVGQEFWYGLERLHQLTKEKSYELMVEMEDFNGSLKYAWYDKFVVGPEEQRYALVELGTFNGTTDGDSLKPHKGSGFSTYDNDDFGCSNKYAKGGWWYYSGKCYGSSLTGIWKNELAYSSIVWMKFSDVSNTPLKLVRMMIRPKN

>AaegCSP42

MVIQRVWRSSANSSTSGNTTATNSNSGALTASSLSATSSSELAPQNVPSGEIDGSQVVVPKMQRPKLSGEAMLEVMKMRYSGRSHSSQSAGDAALKSLELLRANIQYLFDKEIEVVVKKFSSLFFIPAIKNIKENLGESAISDDTLKTLYCSLLENSKSQYVGQIASPAESSLSRANTPGMELSDSDSSNDNVVPSGTTSLLQQALKRKLPEPNQHDGFKRQYFLQGSLYSQNHYSILQSLGNVQGSLPYQIRPSVLNPTVYTTTISPETLFIMDFKAGRALGVPDFRDRLANKHPEILRYCPDNQDRDWLLQQKQISPLNRNGRFFLLVLDEVRKLAERNSEYSNNPYMKLSDLQGFKLTEFIYAKVQKLIKDSADSSVKPTTATPSVTTATTAVPNSIQPRPRVSSLSSSHATLTALLSSPQQSQVNCSNSSGTIATIAGSSTTSGMDANTGGTGSGDTKT

>AaegCSP43

MFSKVKSLSTRGAASTCTVRLVLVVVFMLAISQVAAQSSSTTTPEATSSSGNNGSSPSTPNKSQVSDEALDKALNDKRYLMRQLKCALGEVPCDPVGKRLKSLAPFVLRGACPQCTPAELVQIKKTLAHLQRNFPAEWNKLVQTYAG

>DantCSP1

MKLLFAVVVAAFMATIVLADDKYTTKFDNIDVDEILKSDRLFNNYYKCLTDEGKCTPDGRELKKNLPDALQTECSKCSAKQKESSEKILNFIVENKPEEWKVLQAKYDPEGVYITKYREEAKKRGIKV

>DantCSP2

MFRLIWISLFISYLTFIQAVPHPPATTAAPLKQTYDNKFDNIDIDEILGQERLLKNYVKCLEGLGPCTPDGKMLKDIPILETLPDAMATNCAKCTERQKYGSDRVTHFLIDNRPEDWDRLEKIYNPEGSYKKAYLMEKQKLQPTNEDGDAKKD

>AgamOBP1

MKLVTFVFAALLCCSMTLGDTTPRRDAEYPPPELLEALKPLHDICLGKTGVTEEAIKKFSDEEIHEDEKLKCYMNCLFHEAKVVDDNGDVHLEKLHDSLPSSMHDIAMHMGKRCLYPEGETLCDKAFWLHKCWKQSDPKHYFLV

>AgamOBP2

MLAQASPLLLLLLLLVTQCLDGANCSTITTQRPAPRRDGQYPPPETLAFLRPLGKLCLEETGVSPEAIKRFSDADPFDDNRALKCYMDCMFRVTNVTDDRGELHMGKLLEHVPTEFEDIALRMGVRCTRPKGKDVCERAFWFHKCWKTSDPVHYYLV

>AgamOBP3

MGHDSCWSSRWRVLAALVIFQCAILMVRSDEPRRDANYPPPELLEKMKPMHDACVAETGASEDAIKRFSDQEIHEDDKLKCYMNCLFHQAGVVNDKGEFHYVKIQDFLPESMHLITLNWFKRCLYPEGENGCEKAFWLNKCWKTRDPVHYFLP

>AgamOBP4

MSVSVLVSSLVVLFCVQCLIEHIDGAMTMKQLTNSMDMMRQACAPKFKVEEAELHGLRKSIFPANPDKELKCYAMCIAQMAGTMTKKGEISFSKTMAQIEAMLPPEMKTMAKEALTHCKDTQTSYKDPCDKAYFSAKCAADFTPDTFMFP

>AgamOBP5

MAASRSCWWWRWWWDFILGLVAFFFIPFPSVECAMTRKQLINSMDMMRSACAPKFKVSTEMLDNLRGGIFAEDRELKCYTMCIAQMAGTMNKKGEINVPKTLAQMDAMLPPDMRDKAKEAIHSCRDVQGRYKDSCDKTFYSTKCLAEYDRDVFLFP

>AgamOBP6

MTSNAFYSSNTVTWVVAVIGVYCLVFRPALVHAQQSLTQADMDEIAKGMRKVCMSRHKISEEMANYPSQGIFPDDQEFKCYVACLMDLTQTSKKGKLNYDAAVKQIDILPENYRQPFRLGLDSCRTAADDATDRCEVAYILLKCFFKASPKFFFP

>AgamOBP7

MCEYSNTRNKMSNLVVVLVLLTMYIVLSAPFEIPDRYKKPAKMLHEICIAESGASEEQLRTCLDGTVPTAPAAKCYIHCLFDKIDVVDEATGRILLDRLLYIIPDDVKAAVDHLTRECSHIVTPDKCETAYETVKCYFNAHDEVIKFCHLLVLE

>AgamOBP8

MPSRKRLCRLLLLLLLPVDLELISQDADANVFPAYPVLRNSTPFSIFQTHGAYVVRTFADATAYRDECVQQYAGRGSSLIDYMRQVALHTDNADSRWCIVRCILQKADLLDGEGAPHEANVHAQMQHSNAIVEDPDDIRSETSRCLREPPAPDSGGGCLRAYTFFACIQSTEYDLF

>AgamOBP9

MLKFVVALLAFTAVVSAEFVVQTREDLLAYRAECVKSLGVSDELVEKYKSWNFPEDDTTQCYIKCIFNKMQLFDDTNGPIVDNLVVQLAHGRDANEVREEIVKCAGSNTDGNVCHWAFRGFQCFQKNNLSLIKASVKKD

>AgamOBP10

MVRVLIVFVALLTFAGQPFAVRGQQELSDLPEVKGYKLHCIESSGITESSAKKLAAGESIKEPDQPTKCFVQCFFQKLRLMDEKGVVLKDKLEVFLTKLMDADKAKDYVQQCDLRRTNPCDTAYAVYDCYLGKKAKLF

>AgamOBP11

MIKPFVCILIVAAGCANAFMYKHPYNHHQAAVLAHEPVVPVEFVKHTTSPAFRPASFLEVMEVVLDCFNTLRIPLQRFPSYLSGIFPEDPETKCFLRCVAIKLGVYCDEKGADLDRHCVQFGLGECCENFSNRHLVCLQQNSLPCPDRCTAAYKQELCFQEPIAKYLDYHFHDLVGLLHQAKCSHDLKMLHP

>AgamOBP12

MAPVRYHFVLWLLILIGVSSLVPPGECLDISKVTLDAAFYPLFGCARDLVVPEDLIELYKKRIFPDDQLTCCVFRCLGMRLGIYDDVKGFDVDKQYERVKDRLSVDEDTYKRGVKNCIRNVLRGRTLNNCEKAYLILNQCQGNTITNSLNQQLNEIRCN

>AgamOBP13

MKSFQIATLTVLLVLLAGTASAKKASTIFGMPLQQDPVPATSTFIVSDFLQFLQTAVTCFNKLRIPEERFPLYLAGVFPNCPETQCFVRCLSANLNLYCDETGSDIDRHYLQYGLGQDYNCFRQKAEQCLAANTSPCNDPCEAAYKQELCFLDEFRKYVDSNMNSLIAAVAVEKAEQNPVYYNMLAHN

>AgamOBP14

MKLSSAVLYFALLATAMVCRVQAGSAEELEQAKEMLRGLAAECKTKEGATDEDVEGFVNDKMPESRTQKCLAGCMQEQFGVSNGKAFQEDGFIEIAKMLMKGDETKIELAKEIAADCKAVANDDRCELAVDIMNCLKESAEKHGIELKH

>AgamOBP15

MLTIVVATSICLMATASANAPKSLSPELLQQMGQFRSECLRETGTTDEQIEQFNSPQSVQASHELQCYMYCMFRLHNVTRPNGELDLIDVYHAIPKQFNSIALKVLAKCNKSTGPIADACERAYSHHRCWKETEPELRLPVAVCLMF

>AgamOBP18

MKIELFTLSAPTVPRPGGPHTEGGRNADNFKLYSSLFVFPSPLQGARLEAEHVRRIHQNARECVKETGILPKNAFRVLSGDFSVDTMKAKCFVKCFLDKAGFIDDDGVIQQDVIREKLTVGIEAGKVNELIKKCSVEGTDACDTAYQMYKCFFSNHKVPKELFQMRKGIGRRNMQQ

>AgamOBP19

MAAYLISVVNYSNYGMYITQEQLEKTARTFRQVCQPKHKISDEVADAVNRGVFADTKDFKCYVSCLLDIMQVARKGKVNYEKSLKQIDTMLPDHMKPAFRAGLEACKSAAQGVKDHCEAATILLQCFYKNNPKFVFP

>AgamOBP20

MLFVFFTLLSCTKKKKIFPLRKSTVEQMMKSGEMIRSVCLGKTKVAEELVNGLRESKFADVKELKCYVNCVMEMMQTMKKGKLNYDASVKQIDTIMPDELAGPMRAALDICRTVADGIKNNCDAAYVLLQCLSKNNPKFIFP

>AgamOBP21

MQSLQIVFVVLLAAVSTMEQHEIAKSLAEQCRAELGGELPEDFATKMRLGDLTLDSETAKCTIQCMFAKVGFTLESGAANRDVLIAKLSKGNPTAKAEAFADVCENNEGETACDKAFSLYQCYHKNKSIFD

>AgamOBP22

MNSLLLIGGVLVVLNVQFVTAADNNESVIESCSNAVQGAANDELKVHYRANEFPDDPVTHCFVRCIGLELNLYDDKYGVDLQANWENLGNSDDADEEFVAKHRACLEAKNLETIEDLCERAYSAFQCLREDYEMYQNNNNATSE

>AgamOBP23

MKSFFCVASFFLLVASVHAFTLRQQKMVSIFALECMAETGIGAESLTKLRDGDLTANDRTAKCFMKCFFEKENFMDAEGKLQLEAIATALEKDYERAKIDEMLEKCGEQKEDACETAFNAYACYHDHYQNL

>AgamOBP25

MKFLVFAIVLSAICLDALVDGAAAPPPDLEDVSKIANGEAFALECLIESGLKLDSLAALSAKELDTNGSKIKCLVKCFFEKTGFMNKDGQLQEETITEQLSKFMPRERIESLVKNCNFQEADACETAYKVTECYFQNKAGLF

>AgamOBP26

MKTFVAIAVVALIAGTFALTIDQKKKAEGYAAECVKTTGVPPETAAKLKGGDFAGADDKTKCFAKCFLEKAGFMTDKGEIDEKTVIEKLSVDHDRAKVEGLVKKCNHKEANPCETAFKAYQCIYAAKGAVV

>AgamOBP27

MGRLDLVCLLAIVLLVHSCVSIAWSFSWACTMVKPFVFLLYRMDRTSWDHTSGVAMKPSCFGECFVKRAGFMNDNFTFNRDTIMRFTNRFVSKEISEKVYNICTDNVTPTYCVTAFDVYQCIYENVYKSWDSRK

>AgamOBP28

MKLLFATVLLAVCAAAQPLTDDQMKKAEGFALGCLEQHKGLNKEHLVLLRDGDFSKVDADTKCFLRCFLQQANFMDAAGKLQNDYVIERLSLNREKSKVEALVKKCSAGVEVEDSCETAFRAVECYHREKASLL

>AgamOBP29

MDENTPQKRCVSRAVTVGICGAIVLLLLVGTSPAPVEGLRCRTGEGPSADDVKRIVRTCMNKITNAGGGNFSSSSSSSTIERDRACLMQCFFEEMKATNADGFPEKHKVLHVITKDIREHELREFYVDSIQECFHMLGLDNRLKDKCDYSMRFVTCLSDRFETNCDDWESVTSAMF

>AgamOBP30

MVTQLSQPLPLRGQHTMATVNLYYLGLVCLLAVTATAASQCFRDAGQLKRVVQAQEECVRYLRIPCARLAVYNKFIYPNDAETQCMVRCMGLNLGWWNDTHGVQEASMRSFFHPDPNDCDYERRTYRCLHSQRLDRPAPHDEACERAYESFRCYYEHYGNLVVTPQFVRLNALQQLDVLLQCADMLQYPMPDRSFSCAKTHVAGAEGDFDCVLRCYMLRTGLYSEQYGPNLDRIYVQCNNYANETVFRETTDACYQRLRSDCQDECTLIARYVRECFPAGGIIFLNSLW

>AgamOBP31

MKQLVLLTICVLALMPLEVLSNDTKGLTIEKSFLQSVHDCAEYLQVPKHRLVQYLAYEFPPDEETKCLIFCVGTDLRWWNNTCGLQVPAIMNYFQPVLGDRQYEKRTSECLERNVHTAELPNNCCQAYETFQCYFREFGNLVTCPQYVPATKLQATQAALDCLTVLRVPTDLLQCYSKGDLPDVPETRCLYHCIDHRTGLYTTESGIHLSRFYVRDLEVNDLRYLSKETKACRDRVRMSGCDVCSEVYNTHRDCLSGIGVDGYTSGIIAEASRIALTNLATALSALPARSYAQRSPYPSFHRTCKAEHFGRSF

>AgamOBP32

MISIELKYITLACVLAATVTAGSHCHNDYYQLKSVSQAQEECARYQGIPCARLAVYNKYIYPNDTQTQCMVRCMGLNLGWWNDTHGVQEPAMRSFFHPDPDDCDYERRTYHCLNSQRLNHPSPHVDVCERAYESFRCYYEQYGNIVVTPQFVPLSDLQQVDVLLQCANMLPLTVGRSCAGGSKPSERDVDCLARCFLLRSGLYSEQHGPHLDRLYVQCNNYANETRFRETTGTCYRRLKSECQDECVLAGRFLRECFYEGGISIVNSLPASEASVESAGSLGSGQGSAELGESHQEKVLQTWKDLYDRENLQDLWDRQEL

>AgamOBP33

MATIKLKYITLACVLAATVTAGSHCHNDYYQLKSVSQAQEECARYQGIPCARLAVYNKYIYPNDTQTQCMVRCMGLNLGWWNDTHGVQEPAMRSFFHPDPDDCDYERRTYHCLNSQRLNHPSPHVDVCERAYESFRCYYEQYGNIVVTPQFVPLSDLQQVDVLLQCANMLPLTVGRSCAGGSKPSERDVDCLARCFLLRSGLYSEQHGPHLDRLYVQCNNYANETRFRETTGTCYRRLKSECQDECVLAGRFLRECFYEGGLLGSIPVLGGLGGLVGGLTGLTGLVPPVTLQLTSPGLAAVTLTLSAPSVMVGALPVPPVMVGTLGGAANVGIL

>AgamOBP34

MQFQLNCVQQATRATMNSFALSVFVLAVGAVSVSASLQHYVVEKSFNQAQAECAEYQGVHDDDLLRYVKEGYPDVEEVRCLLRCVAFNLRFWNHTTGLQKNMVAGHFVPYPDDFHNVERTEACLAENLYTCDDDLCTQVYKAFQCYYQYYGALSECPQFVVNSYLEDLQVAYDLFGMLAVSQSTLQSLAGGCFPSGEESLCFFYSFVTRSGLYSVEDGAKLERLYYQYKEEVFNPNNAQTVACLQNQKKLACKKSTCQQAYDTFQNCFGESRGLEYLLHTVFVDAAKAFLGQPVCYCNKVKTCPLHKCYGR

>AgamOBP35

MNFFTVSAIALVAIIGSIQAEHSPLPHYFVRKSFPEAQAECAVYLQVPDDRLQRYMREGYPDEPEVHCLVLCVLENLRAWENGTLHENVLANYFVPATEDCDNAKRTERCLVYLPQECNGEPCVQAYRAFQCYYQNYGTLTTCPEYVPSYYGEDLQLAYDLFDMLDVSEDTRRKLAGGCFPSGPESQCFFFAYVTRFGAWSKDAPLLHNLYTQSQEDAFKKDNAETNVCLTNLNKLACHKTRCEHATDVFSQCFGNTDLYKHFLAVFKDAAMTYTRQ

>AgamOBP36

MNFFTVSAIALVAIIGSIQAEHSPLPHYFVRKSFPEAQAECAVYLQVPDDRLQRYMREGYPDEPEVHCLVLCVLENLRAWENGTLHENVLANYFVPATEDCDNAKRTERCLVNLPQECNGEPCVQAYRAFQCYYQNYGTLTTCPEYVPSYYGEDLQLAYDLFDMLDVSEDTRRKLAGGCFPSGPESQCFFFAYVTRFGAWSKDAPLLHNLYTQSQEDAFKKDNAETNVCLTNLNKLACHKTRCEHATDVFSQCFGNTDLYKHFLAVFKDAAMTYTRQ

>AgamOBP37

MQFQLNCVQQATRATMNSFALSVFVLAVGAVSVSASLQHYVVEKSFNQAQAECAEYQGVHDDDLLRYVKEGYPDVEEVRCLLRCVAFNLRFWNHTTGLQKNMVAGHFVPYPDDFHNVERTEACLAENLYTCDDDLCTQVYKAFQCYYQYYGALSECPQFVVNSYLEDLQVAYDLFGMLAVSQSTLQSLAGGCFPSGEESLCFFYSFVTRSGLYSVEDGAKLERLYYQYKEEVFNPNNAQTVACLQNQKKLACKKSTCQQAYDTFQNCFGESRGLEYLLHTVFVDAAKAFLGQPVCYCNKVKTCPLHKCYGR

>AgamOBP38

MLTYRAWLLLALLGAQCALILGAPATGHGYDTKSFAQAYLECLRYLNISRQSLYAYDSAAVPLNCGSNCLLRCIGLNARWWHDETGLSERALVRFFRQAPADSLLQARACVAELPAPPADSCAGAYWSFRCYSDALGELIAHPAYVAPCGQEIRRAVSDCATMLQVEDGQLQTCVRTETFLRQGNGAALLRCVVLRLGLYADSTGVLCDRVRLLMDADTAEQWTVARAEEAKRCEEDLRALGADTCVVAAHAVELCYGWPAFGELWEVLKQEYGSSDDALAEESEQVVVRRSCTPWMRPLKMGRNRQKARPRRMRKSSCLKMLNRPNWTWQTGRSR

>AgamOBP39

MASSGQVVAAAAVLLLMQLQTVTSATFGARDPPPPALREAQAACVKYLGICENRLHQYNNSVYPTDQDTMCMVRCAGIMVGFWDDCQGLKLDGLANLFPALAANDRVRYQIMSCAEKRIATCPPQDTCARAYNGFRCFLDAQKGGFGAKDMQPQQSTPPQPFDAQEFIRSLSICAKLQRIPKDRRDLYVQGVFPNDDKTRSLIRCVGIRTGLYDDEQGPNIALLYSLFGAGQSESEFRRRANLCIDANQPLLEAQDKNAQAYVKLYRCFADQISALVRANANAMA

>AgamOBP40

MERDRSSSYVAAALLLVCISLASAPRGTEANIFGGKLYQKAQQDCILFMGINPLRLDQYKKFVYPPDRDTMCLIRCIGISLDFWDDILGFDVDLAEQEFSPLVDATFKKYLAGNITLKLELLDPLDNCARAYYAFRTFRAQIRQFIGTGTTTMAPSVNFQPLTAVQILDIIVDCAREVNLPPSFLTSLTKGIITDCPEVQCLIRCAAVRTGLYTDKDGALLANLHRQLDPPGEDLASFSLRQGMCLQRNQQPPTADCCTRAFKQFFTCLRPDFEQFFIRNRETVMQHFLYKTDQPAEDRQPPWCRTMCWIRSGAIWV

>AgamOBP41

MGYWALGTGLQLLLLILVLGGSELQVKAKGSLILRSFDEMVLECAELMSIVHSKLARIRSGVMLPDEDTKCLIRCVGISGRFWNDHTGLRKELLARYFVTDPADAYNVNRTETCLQELPALELNAEKCCGLAFESFLCYYYNYGNLRQDSVFVPLDHLQLQHVTSRCMDVHQITTEQLMSLSAEAMDANDKLHCLVRCIGLQTGVYSDREGVSIDRLNAQYGEGHCEKEFKTHAVECITKHRELAYGSPCKRAYHLLYKCFENVRNVISAYELPDSDGN

>AgamOBP42

MFTTRLLVGALVSLGLTACSFAFTEHGAIVQSIVQAQHECVTYLNLPKHRLYQYLMYNYSNDAKTKQMLRCVGLILQWWKSDGTLNEHVLAQYFMPDTSDSDYYNRTYRCIERKAPVDDDLCSRAFETFQCYLQQYGELLNCPKVVPLSDERLTETMHFCLDVLDIPFSDFEQWTSSSELFLHTEPARCLLRCFTIRAGLYSDQHGPFADRFKLQFGAPKPDVFDNELEGDYCVARLRREGHDACSLAARSLYECYYFADTLLPTFERILPLLRLVLHQPEVETAEME

>AgamOBP43

MCSNRSAFGLLLLAWLASVTILGVEAYATPPPTTANCTTVSTFDAALQECVVQLGIAPERLDQEYNLLLYPADRDTMCLVRCIGVLLRFWNDTTGLREATIRQYYEPAPEDQDYQNRTRSCLAALEPSVTDVCERAHRSFLCYHQHYGYLRKTDRYVPKTPLEMKQIQQDCVDVYGLDPARLNHYQDGQFPDDPETQCFVRCVGLRAGLYTDRDGPNIDRMYVQCESCADETLFRAKAGECIAAQRRHKLSKCTAAYRTLYHCFRDDQLDLYASLTTAAATAAAMTTTTTTKKSTPPNAIPALSVRKPSDRAKLSPDAWQLEIILEGLYNQKY

>AgamOBP44

MKQLVCIVVFALVTPNLIVAECDTKGLIVEKSFLQSVHDCTEYLQIPKERLGQYMANEFPPDDETKCLLFCVGVDLGWWNNTCGLQVPAIVSYFQPVQGDKQYEKRTKECLERRVGAIDSPNSCCQAYETFQCYFQEFGNLVTCPQYVRSTKLQATQAALDCLVMLRYPEKLLKVYASGKVEDSPETRCLYHCIDLRTGLYTQNGISLPRFFVRDAAYNDLRYLSKETKACRDRIRQSGCDKCSEVYNTHTECLSGLGEKGYTSGIITAAAKIALTNLCPAVALSYGGRKPSSTCSKASGTGQVYNLSYPGYKSRMSSCSRCGGRGH

>AgamOBP45

MQRRNASGGGVAVLLTAIMALLPTGCDASLDVPHLTLSKSFSRALQDCMEYLQVPGYRYAEYAANSFPDDPETKCLLRCVGLNLRWWNDTTGMQTAVIEGFFHPDPLDELYENRTAECLRKELSHADTTDCCCLAYDSFRCYLQHYGNLVPCARFYPEDETRFVRAAQDCIEFLQIPHKLLKSYSAGSFPDAPETRCLLRCFFLRTGVFHVDTGFDVERLYTRDYEQPDERYLAQETEARLHKLRGSTGDQCTEVYLAYRDVLGELGRAYYEYDVLQAAAAKMTVCEVAVEPPAMTTTTTTTTTTPTTATACPSTTEFNYKELNCQNCGRLFISNNGRVSCCRCMKSSTPFGKFFF

>AgamOBP46

MNPIVGKVFLVLCGSLLVTGAPNTCGKLDLKTDPFTCCTIPKLLDVTIVSSCFEKFPIDKDAADKGAASMPKTEVTDCMSECILNSTGIYNRRGDVDEKKLNSVFTDSLPANSPWLNVVRKAIKECTAKADKKDKEFQKDVADQKKATPKGTQVCNPEASFLVDCIHTTVFSDCPTNLRSTSTECDAIWNFLKNCPFSALRQ

>AgamOBP47

MKHLKAFDEAQNDIKAVQKRLSTSSTILSGIQKNMAHLNLLQIGVLSLIAVGSVFAGNPCLKGPPVPKNAAECCVTPFLVEPSAFMTCHSKWIGQTKRQMAMEGIPRGCCVAECVMNSTSLYSNGKIDREALTKLYLASTKSMAPEWNKITLDAIDGCFKMADTIKDEIEAGAKLTPAFEGEQICHPISGTILACMGMTLFAECPAKLFTVNDDCNKLKSYHSKCPFL

>AgamOBP48

MGQRQRVVVQLALCFLTFGALLQAGVLAGDNPCAAGPPVDTNPAECCPTPMLVDGTIMMDCYKKYGEQTKKQLQMDGIPRGCCIAECAMNATNMYADGMLKRDDLSKMFMDAVKDKPEWMSLVRDATNACFELAEKKMDEIEAGAKLEPSFEGEKICHPISGTILRCMGMMMFAQCPASVFNVNENCNKLREYGSICPMI

>AgamOBP49

MEWNWTFLFRSFLLLTLHLLPQSVADDCIDMDLHSMEVARCCRYEPISTEEVAEKCYQELAPNIPPNSSDFPVCFIDCSYRQMGYITNEANEIDQSKYGQFLAGFDTAYKIAVERAVAACATVQEDIRRDVANVPSKCNAFALLFHVCVTQITLKHCPDDRWTASEICGKVRMGVPPCA

>AgamOBP50

MHVALPFSVVGKLTCLSPFLQSIKVASCCQLEAFLTLPTYGNCLQTIAEKYPDALWQGTVCAFDCTYREMGILTGVDDINVEQISTNQAGYDQAYQEAIAKAVTACMAQKDKIREEADVVQSECSMFAVKFHACVSLETMRNCPAERWDSSVLCEKVRSGVTVCPL

>AgamOBP51

MCHRVLSLCGFLLLGLQCGWQTLAEDCMDIKIFVSETLQLFRLDGASPKFTAFSSFLQTTKVASCCQLEEFLTLKTYGNCLNTMAEKYPNSTLDYLVCGLDCTYREMGILTGVDDINVEQISTNQAVYGEAYQEAIGKAVDACLAQRDEFREQEKFTKSECGMFALKFQGCIMVESMRNCPAERWDSSVLCEKVRSGVAVCPP

>AgamOBP52

MLFKLFTIPFRCPLFFSKHPKQFPPSKKQSELPYCCQTEPLIPEHVSTKCKEREAANHNPGTELFEVCYQQCIYEELEAVDGLEIRVEKLYALAEGFPADYRHAVHLAIDECVKRLRKTRHMFEQMNAQCSLFGFAVDRCVRLLIYENCPTARWSASVACTKSRQGVPFC

>AgamOBP53

MSFRSISALVILLHLFVICTPMPECISQTQKFEVPHCCQMEELIPRPSRTKCQEKAAIDHNPGFQAYFVVNCLAQCQLEELEVIDGEELHLEKLYPLTAKFPADYRHAVRQAIDECDAWLQGKKKERRRPDGKAHCPLIGMEVENCLHRTTFSNCPNSRWKASITCNKVRQGLPFC

>AgamOBP54

MDLKKSVAVVFVSFGWMMLLATAADPDCENLKNRREEMEQCCQVNMIIPLDGAEDCSSSVDETSEPHDKMMCTLECKLKSLGLLNGDDLVEAKVQEYIDRLEGDWKGTAKTIATECITTITEMKKKIQERDHDMKCSPVGAFFMMCLMKHTQAKCPEDKWQNTSFCNKMRSGECFPKRGRQ

>AgamOBP55

MLPTGLERTVLWVTVIVLVKVMVKSDAQVCCMVEHTFPQEPYRVCHEQHATPQMDNGTVMCIHQCYYKAIGMFAADGKVNTDAYIKYRDELDPTLRDAFSYSMVVCAKIIAKRMNNNIAEVNRMRCSPLPYLFNRCLMEVGIGNCPPERWMNCKHG

>AgamOBP56

MLKLALFVGLVGCVVAYDFQDSFYNEVLMEDLLDNADEPIMFGRFRRSASEVQDDKCKRKYKCCNDANTENMEKIHEIKKQCFMEVRNKNKADGAYEPVDFFSCERLNKTKMEVICAMECVGRKKEVVNEDGTLIEPKLMEFVKSNFAADDWQQPLLAGHIETCVKEAKEKAAKMPREAGQCSSETSNFGYCMWRQMALACPKDKQVANKRCDRIREKLANNEPLHYYKAELEDM

>AgamOBP57

MGKVLILFVGALVVASVTAGRFERSVFAPRIKRDATMRCCNDGFEKSEVHAKFAEVRTACMEELGLGETTHEELIKNREHLNCITECIAKKEGIADENGALLHTDLAKVVLEHMSTIEWKVPLAEGFIQQCFDEVELTDGAFVPSDEAKCNPEGFDFVFCLWRQFTLACPEEFRDDSEKCVELRDKLTNKEDVSDLHDDIEAAE

>AgamOBP58

MSLHLFVRTSTTHGINMRSSSVWLIVVCAVTVASANSEELLRGKENCLRHDDFPSPNECCSKPQWINRYAVRRCRYIHAEVDGSRYERGSCEARCGLFKINMTMTDRIQRVRVYRPRLQTRGIDQGWINVVLKALSYCKPKVTQLQGRHVRTDEEMEQCEIAEDIFGDCVQAQMFMHCPRATWIESRSCQTMRELLATGCPYKTLGEVVVLNDEGYVRDDRILEEEYDRPYRGRGRTESPRYDYDDNDGYSRGGQYDQRGGNYPRGTERNRNGNGYGAGDDGGYVV

>AgamOBP59

MPRLLPEQVIETCRARPLPSVIPGVPDPLPENCIAECALNETGILFNGQFRVEQAVKALSTQVPNDTLTWQHVIEVASKKCYIITVGDSFYLRDVAKNLISPQCIPSSFRFLQCTFSIVYRDCPDIYWNYQNDRCGQFVVALNNCHYLFRHIWDI

>AgamOBP60

MLSFVFLASIIVGLVSSQPPAPDASCFQPTAVTAEDCCKIPKPIDNAIMEKCRAENPKPGQMPAPGVPRTEGCCIVQCAMMETGGFVNNALNTDAIKRSMASTLGADSNFGSLVNGAVDTCARQIQNDPAYSVAPISSSPDRAGCSFIPQGFVNCLYTALFKSCPAATWTESSDCQALKTKLDSGCPFFLLMGRGPRN

>AgamOBP61

MNRLVCAFGVIFVVATLELVLAHPGKDVLGCHNGTSITVDECCAIPMLANKTVIEKCKAAHPFKPPQNTDDKGPRGHPGECLAECIMKGMGALKNEKVDGPAFRKAIEPVVKANPAFAKLLDDTVKQCHESINVDSEFTRYVTKPVCKADAKAFINCVYGTLFEQCPTNVWTQKDGCTQLKDKIKKGCAYFALRKHGGRRMRPT

>AgamOBP62

MKQRCALAGCEKLLPAVLLLLFALQATVPEGTVAGCSMLNNDNAEQRGAAMLADPATVKQVPEVTMQDAIAQCNRSFIIQPEYLAELNQTGSFPEETDKIPLCFIRCYLKALGILTEDDKVNKEVALARNWATSGETVDECLEEMAGSACEQAYFFTRCVMTRALVDGKSKDNK

>AgamOBP63

MKTIACLVLASAFIACAVATISEEQREAARQLAGKCMQQTGASEDDVNRLRSGDTEGADRNTRCFVQCFFQGAGFVDQDGSVQTDELTQKLASEYGQEKADELVARCRNNDGPDACERSFRLLQCYMENRASLMF

>AgamOBP64

MGAFESGLGLLGWVAFGMVLLLAGRGCHAQDFKGAIDHCTKDFEMDMDIVVSLKYGDFTERDPLIECFTECLMKKSGFMYDDYTYNKTLIIGFAGRYLEPEGAQAVYDNCIDRFGQTVCVTGFEMYQCIHETAVSEWVSSNF

>AgamOBP65

MQLAICVWTAVCLQRNIIEGFLVELEAFPSSHQQPPKTSPPVRSCGETFNLTDPRTCCSIPYLLPADVVEPCLEIPLSPIDLAGESNVCVFWQCRAECALNRTEMLVDGHFQLETAMQQLTNATSEDSTLTKRIQYAIGACNELFLNCPPQYWTASDECNQLVRTLNNCPHFLVHTDTF

>AgamOBP66

MATTIARIGSANWAKVLVLLWLVQLATAGEPNPACKTMPTVDKDNEDKCCDVPEMFPNETLNACMEEYQKSSKPPLQKSCEITTCVLKKQSLIKSDNTVDKDKIKSYIKEMVKGSDEWKTLVEKAVLEECLPLMDKDPSNVLSKLKSSLGDCDPAPALTIACAAAKFYVNCPAKDRTKSPMCDEWRTFLSKCSNSLEDLNAIFMVLENQKTR

>AgamOBP67

MNPVVCAFGVIFVVVTLELVVAHPGKDVLGCHNGTSITVDECCAIPMLANKTVIEKCKAAHPFKPPQNTDDKGPRGHPGECIAECIMKGMGALKNEKVDGPAFRKAIEPVVKANPAFAKLLDDTVKQCHESINVDSEFTRYVTKPVCKADAKAFINCVYGTLFEQCPTNVWTQKDGCTQLKDKIKKGCAYFALRKHGGRRMRPT

>AgamOBP68

MATTIARIGSANWAKLLVLLWLVQLATAGEPNPACKTLPTVDKDNEDKCCDVPEMFPNETLNACMEEHQQSSKPPLQKSCEITTCVLKKQSLIKSDNTVDKDKIKSYIKEMVKGSDEWKTLVEKAVLEECLPLMDKDPSNVLSKLKSSLGDCDPAPALTIACAAAKFYVNCPAKDRTKSPMCDEWRTFLSKCSNSLEDLNAIFMVLENQKTR

>AgamOBP83

SITQEQLEKTARTFRQVCQPKHKISDEVADAVNRGVFADTKDFKCYVSCLLDIMQVARKGKVNYEKSLKQIDTMLPDHMKPAFRAGLEACKSAAQGVKDHCEAAAILLQCFYKNNPKFVFP

>CquiOBP1

VTPRRDAEYPPPELLEALKPLHDICAKKTGVTDEAIIEFSDGKIHEDEKLKCYMNCLFHEAKVVDDNGDV

HLEKLHDSLPNSMHDIAMHMGKRCLYPEGENLCEKAFWLHKCWKQADPKHYFLV

>CquiOBP2

MFTTTVLGLGLLLLLQVGLISCEEPRRDAEYPPPEFLVKMKPMHDECVAETGASEDAIKRFSDQEIHEDD

NLKCYMNCLFHKAGVVNDKGEFHYVKIQDFLPESMHLITLNWFKRCLYPQGENLCEKAFWLNKCWKERDP

VHYFLP

>CquiOBP3

MIILSMGLLILDLMFTLAGDLPPPRRDADYPPAYALEFMQQPHRECVAETGVSEAAIKRFSDVEIFDDDE

KLKCYMGCLFVKAGVSDGNGDLHLGKVLELIPKEFEDIALKMGSRCLKPKGKTPCERAFWFNKCWKLADP

VLFPNCL

>CquiOBP4

MSYKLLVLAIGLVQLIRSAECGRNLTELRAANYPPDFLMDLFKNESAVCHNETYVSEETIVRFRDDEDFD

GTPELGCYLYCIFREKNFWINSRNELHLTKALEIVPVDFEQQALKMGLKCLKVKGDDNCARALWYHNCWK

KSSPAHYFLI

>CquiOBP5

MTVATWLSSSVLIRAAPQQRAPDFPPASLIELTVASRKVCVDETGVTEDSIARFNNEPEVFDDDERLKCY

MDCMFRQFNVTKPDTGDVDMIELYHAIPKEYNSVTLKVYNKCRDVVEGSTLCERAFSHHKCWKQNDPVHY

YLF

>CquiOBP6

MERFTFVILVLFLKLLGPSDGAMTMKQLKNSLEMMRKACAPKFKVDDASLNELTAGNFRSDPDPELKCYT

VCIAQMAGTLTKKGEISLSKTTAQIDAMLPNEMKAFAKEAMTACKDAQAGYKETCDKIYYSVKCVAEFNK

DAFLFP

>CquiOBP8

MIWRRFAIVVVGMALLSSNLVESAATMEQLAKSSEMMRTVCMGKTKPPMDQVEGLGQGKFAEGKEIMCYS

NCVLEMMGAMRKGKINADGAIKQVDMLIPAEIGEPTKKAFDICRNAADGVKNNCEAAYALVKCLHKNNPK

YFFA

>CquiOBP9

MSVRAFLPLAMLALLQVFFNPAQSKTTMEQLQKTGEMMRTVCLGKSKASLDQVEALSRGEEQLPEGKEIM

CYANCVLEMMQAMRKGKVIVDSAIKQIDMMLPEDIAEPTVKAFNMCRNSADGIKNNCEAAYAFLKCNRDN

NPKFFFA

>CquiOBP10

MSWRSIMLVTVASMVLLLVNSVESYASMEQLTKSGDMMRSVCMNKAKPSLEHVEGLPSGKFPESKEIMCYANCVLELMQGMRRGKIVADSAMKQADLLIPPEYAEPTKRAFDTCRHAGDGVKNNCEVAYALLKCLHKNNP

KFFFP

>CquiOBP11

MATRVELALLVWIAVWSTGKVEGKATVEQMMKTGEMIRSVCIGKAKASEELVNQLKESKFPDAMEVKCYV

NCALEMMQAMKKGKLNYDAMLKQIDTIMPDELAEPMRNAVNVCRNSADGIKNNCEASYAVAKCISKNNPK

FVFP

>CquiOBP12

MKCDSWATLLLTVLIWYAKFCGHDCGVTKEQMEKTARMFRNVCQPKHKMSDDILEEAKKGVFPNEKNFKC

YVSCLLDMMQATKRGKISYEKSLKQIDTLLPDDFKPDFRNGLEACKDVAQGVKDHCESAYVLLNCFYQNN

PKFIFP

>CquiOBP13

MRYLVILAIGSYVSITGLNHVAGTMTVEDMSRVAKVMRNICQPKFGIPEDVASAASKGVFPDTPEFKCYA

SCLMDLTQTSKKGKLNYDAANAQVQLLPEEYKEPFRIGLDSCRYAADGIEDKCEVAYVLLICFFKATPKF

FFP

>CquiOBP14

MGVKTVIFLLGLLLVCSSSGCSLMNAGDDDGASSDGEQRESLLADPATMGAVKLPKDYSVEDINVECNKT

FIITMEYLNELNDTGSFPDETDKTPMCFMRCFLQKGGILTEDDKVNKEQAFAVGWVKNNETIDDCLQEMT

GTNCERAYFLARCVSTRHLVEGRSKDSKRR

>CquiOBP16

MDKSVVLVVLVAVLVKQCLGQNLLAAYNNCRAEYNADQETFNAIKNGDFSIRTPLVECLGECVVKKVGFMNDDLSFNKDIIVKFVSRFIKPEHSEDIYTKCTQDVAPVLCATAYEVYQCIYENAVDKWGTRRRG

>CquiOBP17 MKSFVAIISLALVASSMAVTEQEKEAARQLAGKCMQQTGASEDSVQRLRNGDTSDADSNTRCFVQCFFQGAGFVDADGNVQEEHVIEKMSAEFDRAKAEEVVSRCRNNAGPNACERSFALLQCYIANRAHLV

>CquiOBP20 MKTFVAIAVIALAAGAWSLTIEQQKKAEAYGAECVKSTGVAPESAVKLRKGDFSGTDDKTKCFAKCVLEKAGFMNAAGDVQEKTVVEKLSIDHDKSKVEATLKKCNQKGANPCDTAFKVYECFYNTKAGLV

>CquiOBP21

MKSVKVSIPWLLLIAIVAVTALPPAKKAEVRRNIRQCSAEADVLTRDSIKVLKGDFKNNGENMKQFVACM

FRKVEFLGDDDEFDEDVIREKLSENIEEDEVSTLMDKCAITKDKLTDTCWERFKCFFKNHKVPSDMMDF

>CquiOBP22

MNQYFVAVCIAAILSQIEAFTLQQRQQGDLFALECLRETAANPVSVALLKIGDFSARDEASKCFVRCFFE

KEGFMDARGNVETENIVAALSNDYDKVKVEALIDRCQVEGREACDTAFQMYECFYRNRESL

>CquiOBP23

MKCLVLIVAALAVGTHAFFTPQQVEFAKKLSADCEAEVGDGLPENIGERFRMGDLTLTDDKSKCYMRCIF

GKVNFLDRETGVINKETLALKLAKGSTQEKAERFAVRCGSFEGANACEKAHGLYECYFTKKDEYFA

>CquiOBP24

MTSYKLLCIFAIFAGNFVSNSHQQDVTKLPDVEGYKLHCIEASGITESSAKKLQSGEDIASPDMATKCYV

QCFFQRLRLMNEKGEVQKDTLTKFLTKVVDEDKAKAMVAKCDTRRTNPCDTAYDMFICYQKNKAKLF

>CquiOBP25

MKFPVALGLFVAVVTADMELTPDERNCQMRQRASDKDVEMFHTMQRPVERTTKCYYDCMMQVMGYSNGKR

FIRDRFEDIYLSAAKNDDQRRTIRHLADKCDGTEHDDACELAADIVACIRR

>CquiOBP26

MKIILAFCLFTAVAALELSPEEKKCQEQVDASEEDVEMFRSLQQPEERTTKCYYDCMLQKINYSDGKRFN

REGFLHTMTQVAKNEQQRKAIQHLGDQCNGTENDDPCELAADIVACLFR

>CquiOBP27

MNIYCTVGLLATLALVVSVSADEARCMREEGASDADAQVLRESRPPVTRTQKCFFDCMYRASRFSDGKRF

TKDGFIAYVMRVVGHDGLKFQVLSAMADKCVHLESDDRCQLAADIEKCLFGQHRKD

>CquiOBP28

MNRLQVALFLTVFVLAHVQADEASDKEQAKEQAKQMLRSMTQKCKEAEGASDDDVEAMIDDVMPESQVQK

CFHSCVQQQFGVSDGQKFLQQGFLEIMLMAVGNDEQQQGHAKEVAEECDGVANENRCQLAVDIMTCVKQG

MEKRGMKVDR

>CquiOBP30

MNRWQLAVALVFFLAAYAFAQTDDELLQIRRNLAQSCKRRGGASDGDVEVLITAVEPVTRVQKCLQSCVQ

QQHGVLDGRRFLKEGYLALMRVVVGTDQLRQRIVEEAADECIGVENENRCQLAIDIMTCVKQALQKTGLR

SKE

>CquiOBP31

MKFSLVLCLLPAVILAQDKQISREEETCREQEGASDEDVEMLQSFMTPETRTTKCYYSCVMQKIGYSDGQ

RFDKEGFLSTAVNFSSKDRETMQRIADQCEGTTNEDHCELAADIAECIIPSRQN

>CquiOBP38

MKSLYLIGLLLVLALAYSKADKQMIRNAAQACKASEGASDEDVDALAEGRMPETQTAKCLFSCVQVQFGL

SDGKKFLKEGFLKHSEAIVGPGEENRRKAEEIAAECEKITNEDRCQLGADIAECIKQGMEKCESKDD

>CquiOBP43

MKLFIAIFALIAVAAADFTVKTTDDLQTYRSECVSSLSISDELVAKYRKWDFPEDDTTQCYIKCIFNKME

LFDDNNGPIVDNLVLQLAHGRDADEVRAEILKCVDKNTDDNSCHWAFRGFKCFQTNNLQLIKASIKKD

>CquiOBP45

MKCSPITLLALASIITLSTATSEQPNWGEVSSTCHKLLRVSPEVGAPHGQDHFSPDPKSACITRCVGIIT

GMYDDETGISMEQLRTWWVDEDTDADFQEFKRRYLACAGSIVPEQYGDDYCKKSSKLYECFMQSGMTVA

>CquiOBP46

MVIPQLAVIVLALQAQLLPSSAWAPHSPEQFRRFEELCMDLAEVPVRQRALLREHLYPDEHRTHCFHRCL

GIVSGLYSDREGADLGRVYAQFGGGRNETRFRDGAERCFRWMLATEMGEGGSGIRLGKCERPYRMHQCFA

KVYREQFEGL

>CquiOBP50

MKFLAATLLIFTVSSQIFDASGADQKPYDKQHWERTNQLCSRLLRTPQEARDLISQKKFDDDANEAMHCV

IRCTGIVSGTYDGERGTVMEMMEVQAQGKTGFAEYRSAAEDCYGGFGPEDYGDDWCKKSYLYFKCDWKAW

VQHVKKVE

>CquiOBP52

MLGLSVTIAKQISYKGALQKSSTHLLGNIESNMLSLRLIAVVAALLAPAGANSLEEQKQKYRQSKGICAKLLRVPADIFERYDRSEYAENHDTYCFIRCVAMLHGHYDDEQGLLVDNLYEVVSLGKSREEFTELMNGCQAENGEEDTKCYCRKAFKPMLCFGRHFNEWRKKVASD

> TjapOBP6

MQSICTKYSFVLSLLVLISSINNAHGVEMNEFQHTLKIVHDTCRTVTKIPEETIQQVRDDNFVDDPVMKCYVHCTMEMLHLMEGSVGQFHVAGKHTDYMIPQELFETTIKAFGHCEGLTNGITDNCEAAYTVLKCFREGNKEFWLP*

> TjapOBP20

MKCLIALLIVCCVIWASKAMTKEEMVAAFKKIASDCAQSEGATDQDMDEIFARKVPSTRAGKCVHACLGEMTGVMKNNKVDVDRAVELAEMAFNGDTTKVNTSRELANECSGVTDTDRCDAAVKIFECGHMAVKSRGITFENV*

> TjapOBP14

MRVILVLLSVLGVSFAALDIPDHLRAPTRVLRKACMAESGIEERHIEASKNGHLENVAEMGCYINCFLEHAGMIDNDGTIHFKDVWHFLTPSMKETVKIVVEECETKHGANKCETAYQTVECYFKTEPEGAELP*

> TjapOBP11

MRPILGYLVFVSFFYVSLGALTEEQMANSAALIRNTCQPKSKLSDDVINRMSDKIFPETKEAKCFVHCVAENMQMMRKAKLNYDSTIKSFDTMLPDTIKDDYKNAITACKDAANGEKNSCEAAYKIMTCFADKNPSFKFV*

> TjapOBP9

MRSILILTVLLSCFNGIYSNLSLDEVMDLMKAFRQQCETKVNVSEELVDGINLGKFPRDNALMCYTHCVLESFNMIRKGKLKADLAIKNVNTFLPPNLREQWVKGITACKDIGDAIAEPCERTFAIIECFYNNNDHFLFP*

>TjapOBP2

MFKHSFLLATVILSVAFAVELRRDDKWPPPEVVAIVQPMRIKCEAKTGVTDEAIRQFSDGEIHEDPALKCYMDCLFKEAGVVDEHGELHLEKVITHIEKLDEEIQMIAINMGRKCLKPQGENQCERAFWYHKCWKTADPKHYFLI*

>TjapOBP3

MNKILLFAIINFILIEYVWCEVRRDDQYPPPQLLAKWKPAHDTCVGKTGVSEEIIKRFSDGDEIFEDDNLKCYMDCLLHEKGFILPDGKIDLVGWHESFNENREIHFTFIHMIRKCLYPKGEGCERAYNLNVCFKTSDLKHYFLV*

> TjapOBP7

MKFIAISLFVFIALLQLSDINARVLSRDDKWPPPGFMEQFEPLHKKCRDTLGLSDEAIMEYDTGDNLEANRTMKCYMSCLFREARAIGENGDPDFKKILPLFDEVNLELKTTVEKIFNACPNISGEDDCERAFSLHKCWKKTGPKYYFLP*

> TjapOBP23

MKYIVAIALMAVAVFASPEWKVQTVDNLNAYRPECATSLSIPEDKVNEYKKFNFPNDEKTQCYIKCIFGKMGLFDEKDGFNIDRLVKQLGQGKNETVIRPEVIKCADKNPQKTNGCQWAYRGFDCFKKAHLELVQNSVKKN*

> TjapOBP13

MKYFVVALCFAVICSISAVTDEQRQIVDRVAKDCASENSLSAEEVQKIRANPKSTVNDAKSQKFGKCFLSKVGFVDSKGDFQENVAIEKLSKGDDKAKIEEIVKSCRSVIGDNKDENPIKLYACYLEKKALA*

> TjapOBP18

MDSLKIVGLVCVLILAVQGEMTEEDRKRMMETGIKCKEECEASDEDLGKLMKKEPVNEKMKCVVGCVVDNSGMMKKNGDNKYEMSCDDMKKKGEDRDLDPSKLGEFHVACKKCNTENKEGDRKNASVALGACIYRETMNQ*

> TjapOBP10

MKTFFCFLFFLSLFYAASAAVSEAQMAETRAMLRNVCKPKSKLSDDVIDKMSDKVFPNDKPSKCFVQCILESMGMMRKSKIQYDAAMKQFDLLLPDSMKESWKDALSACKDSNQGEKNPCEAAYKFVLCFSDKNDSFTFV*

> TjapOBP16

MSIVSDCKEKENASDVDVDVVINHEIPNSPAQNCLSACFYETLGVIQDGKMSIDSLKTVILAKFSADRRAITIINELIDECATVNGNDRCELAHNILECLNNGADKHGINKKQL*

> TjapOBP24

MKLFVIFGVVFAVCMATATVEEHNEFRQKLVDECKGPSHANEDEIKSLQNHEVPQTENGKCLLACIQEKLGILVDGKFSVEGAKALGAKKYEGNPKNIAISHEMVDECTNVADPERCEVAPKIMGCLLNAAVKRGIDPKEGLKN*

> TjapOBP17

MQAVFTFMIVMGILIAFTIAAAPSEEKMKVLNECKAQEKATDSDVQQLLSNKVPQSMNGKCLSACYLEKTGVIKNGKVVADGLKADVMKEYPGGNANGFVSECQGVNNPQRCELGALLTECVNKAAVKYGPK

> TjapOBP22

VFGAILLFVSLVQCEPPKQNIQPNVDPKCLVPPPNDVDPMQCCKIPELLDPKLIETCATKVVSNPSNNQNEPPFAPHIRCVTECILNETGIMKDRMFQPQIASELAKKALGPKSVWMPVIEESIETCNENASKAKFVDHKACGHMGAFILACLNTQIFKNCPESAWNNAGKGCAELRETTIKCPVIAPIPYQD*

> TjapOBP23

MSSTKFFFGMVIVLFSGCAKCYFAADTEQQPASPAVKRVDNIAIDQLDQMAIMQNCNESFRTTMEYLEELNSTGSFPDETDKTPMCYIRCYLDAVGILKDDELNLERAIELQWSTSDETLPECQNEVADRTNPCEKAYFLTRCVMMRNIVDIRTTNE*

> TjapOBP15

NSIFLFILKQFEAEVCSENNRPEDDQKPVIDECKSIENASNEDVENLLYELQTQMPLQTASEKCLLACLYEKLGVIKNGEIQSDGLKIAIMQDSNKETAPIDKIIQECSLISHPERCEQAAQLKNCLDNIITLPTSN

> TjapOBP21

MKSFVVFVFLCCTIGLSESLSKEQRREMFLKMANECAKKEGATSADVDELAAHKPASRKGGKCIKACIAENVGIMKNNKVDVEGTVNVATMAFEGDSSKAQIARDLANACINETDSDRCEAADNIFACGRSNAKGRGISFDDL*

> TjapOBP12

MQMRQSADMVRAVCQPKHKVTDDVVIGAREGKFPDEKSFKCYAQCVLEMMGIMKKSKVSYESALRQFDVLLPEDYKEPYKNALEKCKDSTGGTKNACDAAFNFLNCFYPVNPKFTFA*

> TjapOBP8

MLSKYYSLIYLIVITYHFDWANAAMTMKQIEQSLAMFRKTCSEKVKVDSALIDGLHRGEWPDDNKNLKCFALCIAQTAGTLTKKNELSEQKVKKQVETQLPVNLREFALGAFEACKDIQKSYKDPCDRLYYATKCMYDYSPDNFLYP*

> TjapOBP4

MLLIRFAVILSICFDDFIDAFGIRNLETKPSQKMVDLTKPMHDLCVKMTHVPEEVIQDFVRADISQIEDVDNRLKCYMYCFLHEIEHFDFSNQVDRVADLLTDEENYILISMIDKCESLEFSEDPKFDACDYAFELEKCWKKVDPKHYYIL*

>TjapOBP1

MVRTLCLSLFQYHGLDSNRFFIQFEEAIKEFSDGEIHDDPALKCYMQCVFVEAKAVDQHGEVHLELLQVHIDDLDEEIQNIAIHMGKKCLYPVGETLCDRAFWFHKCWKSTDPKHYFLL*

> TjapOBP19

MVGRCQRKELATESDMSEFSQQKKPTTKTGKCLHACLMETVGLVSNGKFSVEKSVEFMSRMAHGNNELVKIVREISENCSTITDSDRCELAVKIMECTEEGIQKRQEEIKKFMPRRQQEFNHQ*

> TjapOBP5

MFIILLQLSELNGREVRRDDKWPPPVLMKQLEPVRVKCQADIGISDEAIAEFDIGENHEGHREIQCYMSCMFREFNVVHENGDPHLEKIASHFDDVSGELKIAADNLFSACRNIHGVDDCERAFSLHKCWRTTDPKVQKTFDENKTEFTHQRWGGVKIHKAECTECRM*

>DmelOBP18a

MKVVCSIAVLWICLITMWQSAGRVNAEGCLKHHNLTSAQVQAVAPSTPVADVPVAVKCYSRCLIQDYFGDDGKIDLQKVGKRGSQEDHVILSQCKQQFDGVTNLDTCDYPYLILQCYFKGKQSGTIAS

>DmelOBP19a

MKFHLLLVCVAISLGPIPQSEAGVTEEQMWSAGKLMRDVCLPKYPKVSVEVADNIRNGDIPNSKDTNCYINCILEMMQAIKKGKFQLESTLKQMDIMLPDSYKDEYRKGINLCKDSTVGLKNAPNCDPAHALLSCLKNNIKVFVFP

>DmelOBP19b

MTNLLLAVACAAVLMGSATADEEEGSMTVDEVVELIEPFGDACTPKPSRENIVEMVLNKEDAKHETKCFRHCMLEQFELMPEDQLQYNEDKTVDMINMMFPDREDDGRRIVKTCNEELKAEQDKCEAAHGIAMCMLREMRSSGFKIPEIKE

>DmelOBP19c

MKPSTPVAAIPLMTIVVAVLLQTHCVRGQTQAFDLAKLLPKTGTEPIWAVIDRNLPQVQELVTAARMECIQKLQLPRDQRPLGKVTNPSEKEKCLVECVLKKIKLMDADNKLNVGQVEKLTSLVTQDNKMAIAVSSSMAQACSRGISSKNPCEVAHLFNQCISRQLERNNVKLVW

>DmelOBP19d

MSHLVHLTVLLLVGILCLGATSAKPHEEINRDHLLELANECKAETGATDEDVEQLMSHDLPERHEAKCLRACVMKKLQIMDESGKLNKEHAIELVKVMSKHDAEKEDAPAEVVAKCEAIETPEDHCDAAFAYEECIYEQMREHGLELEEH

>DmelOBP28a

MQSTPIILVAIVLLGAALVRAFDEKEALAKLMESAESCMPEVGATDADLQEMVKKQPASTYAGKCLRACVMKNIGILDANGKLDTEAGHEKAKQYTGNDPAKLKIALDIGETCAAITVPDDHCEAAEAYGTCFRGEAKKHGLL

>DmelOBP46a

MCSQLFAFLLLLLTAFVTGRSTPPALDEDCELNSVDTMHDFCCDLHDESPQFSDCQMEWHEKIPYETDEEEQTYMFCTAECSFNSTNFLGRDRRSLNLNEVKEHLESDLVNDADIKLLYDTYVKCDKHALSLMPHKGVKQLSKRLSRLGCHPYPGLVLECVANEMILHCPTKRFRQTAQCEETRNHLKQCMQYLKYKS

>DmelOBP47a

MNRVLVLLLVLKMFALSESRFAKININLGLTVADESPKTITEEMIRLCGDQTDISLRELNKLQREDFSDPSESVQCFTHCLYEQMGLMHDGVFVERDLFGLLSDVSNTDYWPERQCHAIRGNNKCETAYRIHQCQQQLKQQQQNLLATKEVEVTTTPAGSDETKP

>DmelOBP47b

MSPSQLLVIFASLALNTRLVFGQATIDCQRPPQLVDPALCCKDGGRDQVAEQCAQRILGTANGQKAGGPPSLDTAACLAECILTSSKYIDEPQKLNLANIRSDLSAKFSNDTLYVETMTMAFSKCEPQSQRRLAMIMQQQQQVQQQKTQQQQPRCSPFSAIVLGCTYMEYFKNCPDHRWTPNAQCTLAKAYVTQCGLGA

>DmelOBP49a

MLSKSQLLLLVVGFCLNAAVSADVDCSKRPSFVNPKTCCPMPDFVTAELKQKCIKFDMTPPPPPDGEASGSFESKRRHHHPHPPPCFFSCIFNETGIYQNRKLDEAKLNAYLQEVFEDSSDLQTTATQAFTTCATKVADFEANLPPRPAPSPPPGFPMCPHDAGHLMGCVFRNMMKNCPDSIRNDSQQCTDMKEFFTKCKPPRGPPPSAEDM

>DmelOBP50a

MRTGRILVALIFLGLIIPFRAAKCRAAPKSVQNVHVCCSAPLPNWGVFNRECHKSAIQASVSINRISKSKVNLANFLIKCRLDCDFNASSVLQGNRLIQAKVRPMLERAFSNEPTIDAYESNFAKCSTVVRSKYQELSPLSRQSDACDRHALFYSLCAYARLIFTCPDKMWQRNNRMCQEAKAYAKKCPWPALKMFMRNT

>DmelOBP50b

MSSVLHLLGFLWLPLLVYSVSNDMGGLQKCTELLNTHKLVYCCGKSFLDKFPFVGSNCTPFWDDYGPCRYECLYRHWDLLDQDNKIKKPELYLMITSLYSPLNGYDKYGAAFKAAHETCEALGSRHADFLLLYSNQVADKMGMASSTCLPYAMLHAQCTMVYLTANCPRENWIDDPKCNSLQKLLSSCTKKLDEKTNALKGKDEELTDNGCGHIDSEGSNLLMACFLTLMIAKFISDH

>DmelOBP50c

MARHIALLICSLLAMAGCDPIDVDCTRRQDFNIVKDCCVYPTFRFDQFKSQCGKYMPVGAPRISPCLYECIFNKTNTVVDGAIHPDNARLMLEKLFGNQDFEEAYFNGLMGCSDSVQEMISNRRSRPQRKTEQCSPFSLFYGICAQRYVFNHCPSSSWSGTESCEMARLQNMNCSKPSRGSSHRL

>DmelOBP50d

MLHKLTWVLIFIPAFRAADPICSQRPDVTALRNCCKLPNLDFSSFNSKCSQYLVNGVHISPCSFECIFRAANALNGTHLVMENIEKMMKTILGSDEFVHVYLDGFRSCGNQEKVLIKAMKRRRVPITGKCGSMAIMYGLCAHRYVYRNCPESVWSKSATCNEAREYSIRCDDM

>DmelOBP50e

MHKYIICFGFLLIILECSLASFNCSAPPNFNNFDINTCCRTPELDMGDVPQKCHKYVSGLKSANSKYPSYAHLCYPDCIYRETGAMVNGKIKVNRVKQYLEEHVHRRDQEIVSHIVQSFESCLSNVKGHMKSLNIESYKVLPHGCSPFAGIIYSCVNAETFLNCPQQMWKNEKPCNLAKQFAEQCNPLPHVPLPSS

>DmelOBP51a

MKVFIGLVLLLAVTTLSSALFESEANECAKKLGITPDYFENFPHSSRVKCFYHCQMEKLEIIANGVVTPFDLKVLNISPESYDKYGVKVKPCLKLSHRDKCELGYLVFQCLKREFNL

>DmelOBP56a

MNSYFVIALSALFVTLAVGSSLNLSDEQKDLAKQHREQCAEEVKLTEEEKAKVNAKDFNNPTENIKCFANCFFEKVGTLKDGELQESVVLEKLGALIGEEKTKAALEKCRTIKGENKCDTASKLYDCFESFKPAPEAKA

>DmelOBP56b

MKLIYLLVVFLIFALSELVAGQSAAELAAYKQIQQACIKELNIAASDANLLTTDKEVANPSESVKCYHSCVYKKLGLLGDDGKPNTDKIVKLAQIRFSSLPVDKLKSLLTSCGTTKSAATCDFVYNYEKCVVKGISA

>DmelOBP56d

MKFLIVLSVILAISAAELQLSDEQKAVAHANGALCAQQEGITKDQAIALRNGNFDDSDPKVKCFANCFLEKIGFLINGEVQPDVVLAKLGPLAGEDAVKAVQAKCDATKGADKCDTAYQLFECYYKNRAHI

>DmelOBP56e

MKVFFVFAALAALSLASAVGLTDSQKAEAKQRAKACVKQEGITKEQAIALRSGNFADSDPKVKCFANCFLEQTGLVANGQIKPDVVLAKLGPIAGEANVKEVQAKCDSTKGADKCDTSYLLYKCYYENHAQF

>DmelOBP56f

MKVFLLFIFISAIWLQAFCMKSSEKIKACLKRQLGYTITENTKFDAKEDSLQSKCFYHCLLEVKGVIANDAISSEQPRKVLEKKYGITDTDELEKAEEKCHSIKASGKCELGYEILKCYQSITKH

>DmelOBP56g

MRATFALTLLLGCLSGILAQQANIDSSVSKELVTDCLKENGVTPQDLADLQSGKVKAEDAKDNVKCSSQCILVKSGFMDSTGKLLTDKIKSYYANSNFKDVIEKDLDRCSAVKGANACDTAFKILSCFQAAN

>DmelOBP56h

MKFTLFCIALAAFLSMGQCNPDFRQIMQQCMETNQVTEADLKEFMASGMQSSAKENLKCYTKCLMEKQGHLTNGQFNAQAMLDTLKNVPQIKDKMDEISSGVNACKDIKGTNDCDTAFKVTMCLKEHKAIPGHH

>DmelOBP56i

MHFFTCCALLLVVVTLPTCFVQAGPIKDQCMAAAGITAQDVANRHETDDPGHSVKCFFRCFLENIGIIADNQIIPGAFDRVLGHIVTAEAVERMEATCNMIKSETSHDESCEFAWQISECYEGVRLSDVKKGQRTRNHRG

>DmelOBP57a

MLKLWLICILTVSVVSIQSLSLLEETNYVSDCLASNNISQAEFQELIDRNSSEEDDLENTDRRYKCFIHCLAEKGNLLDTNGYLDVDKIDQIEPVSDELREILYDCKKIYDEEEDHCEYAFKMVTCLTESFEQSDEVTEAGKNTNKLNE

>DmelOBP57b

MFIYRLVFIAPLILLLFSLAKARHPFDIFHWNWQDFQECLQVNNITIGEYEKYARHETLDYLLNEKVDLRYKCNIKCQLERDSTKWLNAQGRMDLDLMNTTDKASKSITKCMEKAPEELCAYSFRLVMCAFKAGHPVIDSE

>DmelOBP57c

MFNTRLAIFLLLIVVSLSQAKESQPFDFFEGTYDDFIDCLRINNITIEEYEKFDDTDNLDNVLKENVELKHKCNIKCQLEREPTKWLNARGEVDLKSMKATSETAVSISKCMEKAPQETCAYVYKLVICAFKSGHSVIKFDSYEQIQEETAGLIAEQQADLFDYDTIDL

>DmelOBP57d

MLDQLTLCLLLNFLCANVLANTSVFNPCVSQNELSEYEAHQVMENWPVPPIDRAYKCFLTCVLLDLGLIDERGNVQIDKYMKSGVVDWQWVAIELVTCRIEFSDERDLCELSYGIFNCFKDVKLAAEKYVSISNAK

>DmelOBP57e

MSLRLVPHLACIIFILEIQFRIADSNDPCPHNQGIDEDIAESILGDWPANVDLTSVKRSHKCYVTCILQYYNIVTASGEIFLDKYYDTGVIDELAVAPKINRCRYEFRMETDYCSRIFAIFNCLRQEILTKS

>DmelOBP58a

MKQLIFLLICLSCGTCSIYALKCRSQEGLSEAELKRTVRNCMHRQDEDEDRGRGGQGRQGNGYEYGYGMDHDQEEQDRNPGNRGGYGNRRQRGLRQSDGRNHTSNDGGQCVAQCFFEEMNMVDGNGMPDRRKVSYLLTKDLRDRELRNFFTDTVQQCFRYLESNGRGRHHKCSAARELVKCMSEYAKAQCEDWEEHGNMLFN

>DmelOBP58b

MLRIGFVICVIISLRLNGLVAVRVHCRHMERIHEENIHHCCKHQDGHDDVTESCAKQTNFRLPSPNEEAIVDVTVDQAMVGTCWAKCVFDHYNLMENNTLDMDKVRSYYKRYHQTDPEYATEMLNAYEKCHTQSEEATEKFLSLPIVRAFSTAKFCKPTSSIIMSCVIYNFFHNCPASRWSNTTECVETLAFARKCKDVLTT

>DmelOBP58d

MVNIVCYWTFLILVAVSKAQDNEETTAVAISSGDLTEDKCNTSRAGCCSELYIGEEEDLVKCFVIHSPKLPVDGDADIGKTLRFLSCFVECLYKQKKYIGKSDTINMKMVKLDAEKTFVDRPKEKDYHIAMFEFCRKDAVGVYNLLKASPGAKVLLKGACRPYLLMVFMCISDYHQKHECPYFRWEGTAKAGTKDMCENAKAECYQIDGITLPTKSPA

>DmelOBP69a

MVARHFSFFLALLILYDLIPSNQGVEINPTIIKQVRKLRMRCLNQTGASVDVIDKSVKNRILPTDPEIKCFLYCMFDMFGLIDSQNIMHLEALLEVLPEEIYKTINGLVSSCGTQKGKDGCDTAYETVKCYIAVNGKFIWEEIIVLLG

>DmelOBP76a

MKHWKRRSSAVFAIVLQVLVLLLPDPAVAMTMEQFLTSLDMIRSGCAPKFKLKTEDLDRLRVGDFNFPPSQDLMCYTKCVSLMAGTVNKKGEFNAPKALAQLPHLVPPEMMEMSRKSVEACRDTHKQFKESCERVYQTAKCFSENADGQFMWP

>DmelOBP83a

MALNGFGRRVSASVLLIALSLLSGALILPPAAAQRDENYPPPGILKMAKPFHDACVEKTGVTEAAIKEFSDGEIHEDEKLKCYMNCFFHEIEVVDDNGDVHLEKLFATVPLSMRDKLMEMSKGCVHPEGDTLCHKAWWFHQCWKKADPKHYFLP

>DmelOBP83b

MVKYPLILLLIGCAAAQEPRRDGEWPPPAILKLGKHFHDICAPKTGVTDEAIKEFSDGQIHEDEALKCYMNCLFHEFEVVDDNGDVHMEKVLNAIPGEKLRNIMMEASKGCIHPEGDTLCHKAWWFHQCWKKADPVHYFLV

>DmelOBP83d

MESHPFTVIDNMPNISPSAKDAMKDCLQDVHQDEWKSFDAFAYYPVNEPIPCFTRCFVDKLHIFEEKTRLWKLEAMKQNLGIPAKGARIRTCHRHRGRDRCATYYKQFTCYAMAV

>DmelOBP83f

MQSQSLLLIVAAVATFLVAQVRAQWLPLLMETTAKFLLKDHADAEKAFEECREDYYVPDDIYEKYLNYEFPAHRRTSCFVKCFLEKLELFSEKKGFDERAMIAQFTSKSSKDLSTVQHGLEKCIDHNEAESDVCTWANRVFSCWLPINRHVVRKVFA

>DmelOBP84a

MYSALVRACAVIAFLILSPNCARALQDHAKDNGDIFIINYDSFDGDVDDISTTTSAPREADYVDFDEVNRNCNASFITSMTNVLQFNNTGDLPDDKDKVTSMCYFHCFFEKSGLMTDYKLNTDLVRKYVWPATGDSVEACEAEGKDETNACMRGYAIVKCVFTRALTDARNKPTV

>DmelOBP93a

MKTSNKIVFLLLQLNIWQLSSCCDVQKNDKAINSCRKSLLGNNSTNSNGEVRNLKSDKVALHACIAECSFRTNGFLLSNGTVNTQALQKSYQQRYKNDPNMSQLMLKSLNSCTDYARKRVQEFQWMPKKGDCDFYPATLLACVMEKVYINCPTSKWKNTSDCTAMWKYLVACDDVASNKKK

>DmelOBP99a

MKVFVAICVLIGLASADYVVKNRHDMLAYRDECVKELAVPVDLVEKYQKWEYPNDAKTQCYIKCVFTKWGLFDVQSGFNVENIHQQLVGNHADHNEAFHASLAACVDKNEQGSNACEWAYRGATCLLKENLAQIQKSLAPKA

>DmelOBP93c

MNHLRLEIICWSCLLIAMAVSTEAASVWKLPTAQMVYEDLEKCRQESQEEDAATLRCLVKKLGLWTDESGYNARRIAKIFAGHNQMEELMLVVEHCNRMEQDTSHLDDWAFLAYRCATSGQFGHWVKDFMSQKEVER
