# Supplementary material for: Identification and expression profile analysis of chemosensory genes in pine needle gall midge, Thecodiplosis japonensis (Diptera: Cecidomyiidae)
Source: Front Physiol. 2023 Feb 16;14:1123479. doi: 10.3389/fphys.2023.1123479 (PMC9978445; doi:10.3389/fphys.2023.1123479)
Supplement: Supplementary file 1 [file DataSheet1.ZIP › Supplementary Tables.docx]

Table S1 Blastx matches for genes encoding candidate OBPs in *Thecodiplosis japonensis*

| Gene  Name | Gene  length  (bp) | ORF  (aa) | Complete  ORF | Signal  Peptide  (aa) | Cysteine  number |  | FPKM | Best blastx match | | | | |
| --- | --- | --- | --- | --- | --- | --- | --- | --- | --- | --- | --- | --- |
|  |  |  |  |  |  |  |  | Gene description | species | Accession  number | Identity  % | E-value |
| TjapOBP1 | 360 | 120 | Yes | No | 6 | Classic | 423.6 | odorant binding protein 15 | *Sitodiplosis mosellana* | AHW83257.1 | 86.3 % | 4.6e-46 |
| TjapOBP2 | 438 | 146 | Yes | 18 | 6 | Classic | 574.12 | odorant binding protein 12 | *Sitodiplosis mosellana* | AXS77544.1 | 79.31 % | 2e-81 |
| TjapOBP3 | 438 | 146 | Yes | 20 | 7 | Classic | 550.37 | odorant binding protein 11 | *Sitodiplosis mosellana* | AXS77545.1 | 83.45 % | 9e-77 |
| TjapOBP4 | 456 | 152 | Yes | 20 | 7 | Classic | 24.4 | odorant binding protein 20 | *Episyrphus balteatus* | QIS77213.1 | 38.17 % | 3e-19 |
| TjapOBP5 | 507 | 169 | Yes | No | 8 | Classic | 40.29 | odorant binding protein 8 | *Bradysia odoriphaga* | AWC08419.1 | 45.52 % | 1e-35 |
| TjapOBP6 | 441 | 147 | Yes | 26 | 7 | Classic | 75423.09 | odorant binding protein 18 | *Sitodiplosis mosellana* | AHW83256.1 | 72.60 % | 4e-70 |
| TjapOBP7 | 453 | 151 | Yes | 22 | 6 | Classic | 387.23 | odorant binding protein 8 | *Bradysia odoriphaga* | AWC08419.1 | 40.69 % | 9e-33 |
| TjapOBP8 | 444 | 148 | Yes | No | 6 | Classic | 600.4 | odorant binding protein 25 | *Propsilocerus akamusi* | QGW50689.1 | 51.56 % | 1e-45 |
| TjapOBP9 | 423 | 141 | Yes | 19 | 7 | Classic | 12417.98 | odorant binding protein 17 | *Sitodiplosis mosellana* | AXS77546.1 | 53.57 % | 2e-55 |
| TjapOBP10 | 423 | 141 | Yes | 19 | 7 | Classic | 6647.6 | odorant binding protein 11 | *Bradysia odoriphaga* | AWC08422.1 | 44.14 % | 2e-29 |
| TjapOBP11 | 423 | 141 | Yes | 19 | 6 | Classic | 14369.89 | odorant binding protein 1 | *Liriomyza sativae* | ALZ41694.1 | 45.53 % | 2e-35 |
| TjapOBP12 | 354 | 118 | Yes | No | 6 | Classic | 16.6 | odorant binding protein 11 | *Bradysia odoriphaga* | AWC08422.1 | 50.00 % | 2e-29 |
| TjapOBP13 | 399 | 133 | Yes | 18 | 6 | Minus-C | 701.27 | odorant binding protein 14 | *Aedes albopictus* | AGI04314.1 | 35.61 % | 3e-17 |
| TjapOBP14 | 405 | 135 | Yes | 16 | 6 | Classic | 19702.29 | odorant binding protein 20 | *Sitodiplosis mosellana* | AHW83247.1 | 61.67 % | 2e-52 |
| TjapOBP15 | 417 | 139 | Yes | 23 | 6 | Classic | 10.3 | odorant binding protein 14 | *Sitodiplosis mosellana* | AHW83245.1 | 34.56 % | 2e-13 |
| TjapOBP16 | 345 | 115 | Yes | No | 6 | Classic | 2842.9 | odorant binding protein 14 | *Sitodiplosis mosellana* | AHW83245.1 | 39.64 % | 1e-21 |
| TjapOBP17 | 399 | 133 | Yes | 20 | 6 | Classic | 8.6 | odorant binding protein 14 | *Sitodiplosis mosellana* | AHW83245.1 | 37.78 % | 1e-20 |
| TjapOBP18 | 423 | 141 | Yes | 19 | 9 | Minus-C | 126.71 | general odorant binding protein 28a | *Drosophila teissieri* | XP_043641299.1 | 30.71 % | 3e-04 |
| TjapOBP19 | 372 | 124 | Yes | No | 6 | Classic | 1.76 | odorant binding protein 6 | *Propsilocerus akamusi* | QGW50670.1 | 38.68 % | 1e-17 |
| TjapOBP20 | 432 | 144 | Yes | 19 | 9 | Classic | 24245.72 | odorant binding protein 13 | *Sitodiplosis mosellana* | AHW83258.1 | 45.45 % | 4e-28 |
| TjapOBP21 | 432 | 144 | Yes | 19 | 9 | Classic | 0.56 | odorant binding protein 13 | *Sitodiplosis mosellana* | AHW83258.1 | 49.55 % | 1e-28 |
| TjapOBP22 | 465 | 155 | Yes | No | 11 | Plus-C | 1.1 | odorant binding protein 34 | *Bradysia odoriphaga* | AWC08445.1 | 34.87 % | 7e-25 |
| TjapOBP23 | 426 | 142 | Yes | 16 | 6 | Classic | 0.15 | odorant binding protein 14 | *Bradysia odoriphaga* | AWC08425.1 | 49.65 % | 2e-43 |
| TjapOBP24 | 435 | 145 | Yes | 18 | 7 | Classic | 25.3 | odorant binding protein 14 | *Sitodiplosis mosellana* | AHW83245.1 | 60.54 % | 2e-56 |
| TjapOBP25 | 474 | 158 | Yes | 24 | 8 | Classic | 3825.4 | odorant binding protein 16 | *Sitodiplosis mosellana* | AHW83254.1 | 68.07 % | 1e-75 |
| TjapOBP26 | 366 | 122 | No | No | 7 | Classic | 5.7 | odorant binding protein 13 | *Sitodiplosis mosellana* | AHW83258.1 | 49.35 % | 2e-15 |

Table S2 Blastx matches for genes encoding candidate CSPs in *Thecodiplosis japonensis*

| Gene  Name | Gene  length  (bp) | ORF  (aa) | Complete  ORF | Signal  Peptide  (aa) | Cysteine  number | FPKM | Best blastx match | | | | |
| --- | --- | --- | --- | --- | --- | --- | --- | --- | --- | --- | --- |
|  |  |  |  |  |  |  | Gene description | species | Accession  number | Identity  % | E-value |
| TjapCSP1 | 366 | 122 | Yes | 19 | 5 | 244.9 | chemosensory protein 4 | *Bradysia odoriphaga* | AWC08464.1 | 66.07 % | 9e-50 |
| TjapCSP2 | 336 | 112 | Yes | 28 | 5 | 17.74 | chemosensory protein 2 | *Bradysia odoriphaga* | AWC08462.1 | 77.48% | 4e-54 |

Table S3 Blastx matches for genes encoding candidate ORs in *Thecodiplosis japonensis*

| Gene Name | Gene  length (bp) | ORF (aa) | Complete ORF | FPKM | TMD  (No.) | Best blastx match | | | | |
| --- | --- | --- | --- | --- | --- | --- | --- | --- | --- | --- |
|  |  |  |  |  |  | Gene description | species | Accession number | Identity% | E-value |
| TjapOR1 | 840 | 280 | No | 5.3 | 5 | odorant receptor 112 | *Mayetiola destructor* | AOT85629.1 | 25.10 % | 3e-13 |
| TjapOR2 | 1221 | 407 | Yes | 12.55 | 6 | odorant receptor Or1 isoform X2 | *Tribolium castaneum* | XP_015833040.1 | 20.8 % | 5.9e-8 |
| TjapOR3 | 1185 | 395 | Yes | 20.34 | 4 | odorant receptor 112 | *Mayetiola destructor* | AOT85629.1 | 36.4 % | 1.5e-57 |
| TjapOR4 | 1143 | 381 | Yes | 14.02 | 5 | Odorant receptor 83c | *Culex quinquefasciatus* | XP_001846628.1 | 26 % | 2.9e-25 |
| TjapOR5 | 1035 | 345 | Yes | 31.43 | 6 | odorant receptor 43 | *Aedes aegypti* | NP_001345114.1 | 21.9 % | 1.9e-7 |
| TjapOR6 | 942 | 314 | No | 3.57 | 4 | putative odorant receptor 85d | *Linepithema humile* | XP_012223367.1 | 23.6 % | 2.1e-11 |
| TjapOR7 | 969 | 323 | Yes | 5.2 | 5 | Odorant receptor 83c | *Culex quinquefasciatus* | XP_001846628.1 | 28.4 % | 3.3e-19 |
| TjapOR8 | 1287 | 429 | Yes | 1.74 | 6 | odorant receptor 13a | *Camponotus floridanus* | XP_025266890.1 | 26.8 % | 0.0000021 |
| TjapOR9 | 915 | 305 | No | 4.57 | 4 | odorant receptor 85b-like | *Halyomorpha halys* | XP_024214259.1 | 21 % | 0.0000013 |
| TjapOR10 | 951 | 317 | Yes | 29.27 | 4 | odorant receptor 13a | *Ceratitis capitata* | XP_004529819.1 | 43.5 % | 2.9e-50 |
| TjapOR11 | 795 | 265 | No | 3.13 | 3 | odorant receptor, partial | *Calliphora stygia* | AID61235.1 | 24.6 % | 8.7e-7 |
| TjapOR12 | 1185 | 395 | Yes | 21.32 | 6 | odorant receptor 22c | *Bactrocera dorsalis* | XP_011210110.1 | 27.3 % | 1.3e-7 |
| TjapOR13 | 1158 | 386 | Yes | 1.65 | 7 | odorant receptor | *Campoletis chlorideae* | AXM05145.1 | 25.9 % | 3e-8 |
| TjapOR14 | 1146 | 382 | Yes | 6.69 | 8 | odorant receptor 40 | *Bradysia odoriphaga* | QGW45413.1 | 27.27 % | 3e-31 |
| TjapOR15 | 1176 | 392 | Yes | 388.78 | 6 | odorant receptor 46a | *Pieris rapae* | XP_022124400.2 | 23.94 % | 6e-12 |
| TjapOR16 | 1215 | 405 | Yes | 7 | 7 | odorant receptor 67a | *Drosophila subobscura* | XP_034663430.1 | 24.35 % | 6e-06 |
| TjapORCO | 1401 | 467 | Yes | 106.96 | 6 | odorant receptor co-receptor Orco | *Mayetiola destructor* | AOT85634.1 | 90.99 % | 0 |

Table S4 Blastx matches for genes encoding candidate GRs in *Thecodiplosis japonensis*

| Gene Name | Gene  length (bp) | ORF (aa) | Complete ORF | FPKM | TMD  (No.) | Best blastx match | | | | |
| --- | --- | --- | --- | --- | --- | --- | --- | --- | --- | --- |
|  |  |  |  |  |  | Gene description | species | Accession number | Identity% | E-value |
| TjapGR1 | 1128 | 376 | Yes | 401 | 7 | gustatory and odorant receptor 22 | *Anopheles funestus* | XP_049301598.1 | 70.80 % | 7e-178 |
| TjapGR2 | 1383 | 461 | Yes | 9.75 | 7 | gustatory receptor 24 | *Culex quinquefasciatus* | XP_001848689.1 | 65.9 % | 7.2e-148 |
| TjapGR3 | 357 | 119 | No | 0.39 | 2 | gustatory receptor 61 isoform Gr61b | *Aedes aegypti* | NP_001345536.1 | 31.93 % | 7e-13 |
| TjapGR4 | 789 | 263 | No | 0.63 | 4 | putative gustatory receptor 2a | *Drosophila novamexicana* | XP_030566982.1 | 25.4 % | 0.031 |
| TjapGR5 | 204 | 68 | No | 0 | 1 | gustatory receptor for sugar taste 64e | *Bactrocera dorsalis* | XP_049315202.1 | 66.67 % | 3e-10 |
| TjapGR6 | 582 | 194 | No | 0.47 | 3 | gustatory receptor for sugar taste 43a | *Hermetia illucens* | XP_037917322.1 | 62.66 % | 8e-61 |

Table S5 Blastx matches for genes encoding candidate IRs in *Thecodiplosis japonensis*

| Gene Name | Gene  length (bp) | ORF (aa) | Complete ORF | FPKM | TMD  (No.) | Best blastx match | | | | |
| --- | --- | --- | --- | --- | --- | --- | --- | --- | --- | --- |
|  |  |  |  |  |  | Gene description | species | Accession number | Identity% | E-value |
| TjapIR21a | 1641 | 547 | Yes | 12.17 | 4 | ionotropic receptor 21a | *Bradysia coprophila* | XP_037033092.1 | 63.32 % | 4e-152 |
| TjapIR47a | 492 | 164 | No | 1.04 | 0 | ionotropic receptor 93a | *Heliconius melpomene* | AMM70658.1 | 45.2 % | 2.4e-39 |
| TjapIR64a | 1812 | 604 | Yes | 11.2 | 1 | putative ionotropic receptor IR8 | *Scaeva pyrastri* | AOE48112.1 | 47.3 % | 2.5e-61 |
| TjapIR8a | 2274 | 758 | Yes | 24.9 | 2 | putative ionotropic receptor IR10 | *Scaeva pyrastri* | AOE48114.1 | 49.6 % | 5.2e-187 |
| TjapIR25a | 2805 | 935 | Yes | 6.84 | 3 | ionotropic receptor 25a | *Aedes aegypti* | NP_001345261.1 | 76.8 % | 0 |
| TjapIR93a | 564 | 188 | No | 5.07 | 1 | ionotropic receptor 93a | *Thrips palmi* | XP_034244940.1 | 49.20 % | 2e-52 |
| TjapIR75d3 | 1620 | 540 | No | 7.03 | 5 | putative ionotropic receptor IR3 | *Scaeva pyrastri* | AOE48107.1 | 47.6 % | 2.4e-122 |
| TjapIR75d5 | 1716 | 572 | Yes | 46.44 | 2 | ionotropic receptor 24 | *Locusta migratoria* | ALD51351.1 | 34.6 % | 1.2e-86 |
| TjapIR75d6 | 1371 | 457 | Yes | 4.4 | 3 | putative ionotropic receptor IR7 | *Scaeva pyrastri* | AOE48111.1 | 37 % | 1.9e-76 |
| TjapIR75d2 | 759 | 253 | No | 78.31 | 4 | ionotropic receptor | *Anoplophora chinensis* | AUF73086.1 | 41.8 % | 4.5e-44 |
| TjapIR76b | 1935 | 645 | No | 4.8 | 3 | putative ionotropic receptor IR12 | *Scaeva pyrastri* | AOE48116.1 | 48.4 % | 6.1e-155 |
| TjapIR75d4 | 846 | 282 | No | 25.11 | 1 | ionotropic receptor 75d | *Heortia vitessoides* | XP_023934974.1 | 26.8 % | 1.1e-12 |
| TjapIR75d1 | 939 | 313 | No | 11.9 | 3 | ionotropic receptor | *Ostrinia furnacalis* | BAR64808.1 | 43.14 % | 3e-59 |

Table S6 Blastx matches for genes encoding candidate SNMPs in *Thecodiplosis japonensis*

| Gene Name | Gene  length (bp) | ORF (aa) | Complete ORF | FPKM | TMD  (No.) | Best blastx match | | | | |
| --- | --- | --- | --- | --- | --- | --- | --- | --- | --- | --- |
|  |  |  |  |  |  | Gene description | species | Accession number | Identity% | E-value |
| TjapSNMP2 | 684 | 228 | No | 1.4 | 1 | sensory neuron membrane protein 2 | *Bactrocera dorsalis* | AKI29052.1 | 45.8 % | 1.8e-58 |
| TjapSNMP1a | 1374 | 458 | Yes | 1.48 | 1 | sensory neuron membrane protein 1f | *Mayetiola destructor* | AOT85639.1 | 52.4 % | 1.7e-119 |
| TjapSNMP1b | 1479 | 493 | Yes | 30.72 | 3 | sensory neuron membrane protein 1a | *Mayetiola destructor* | AOT85635.1 | 44 % | 3.9e-141 |
